# Supplementary material for: Understanding and maximising the community impact of seasonal malaria chemoprevention in Burkina Faso (INDIE-SMC): study protocol for a cluster randomised evaluation trial
Source: BMJ Open. 2024 Mar 12;14(3):e081682. doi: 10.1136/bmjopen-2023-081682 (PMC10936478; doi:10.1136/bmjopen-2023-081682)
Supplement: Supplementary data [file bmjopen-2023-081682supp001.pdf]

Head of Compound information and Informed Consent Documents

TITLE OF STUDY: INDIE_SMC Understanding and maximizing the community impact of antimalarial treatment

INSTITUTIONS: Groupe de Recherche Action en Santé (GRAS); London School of Hygiene & Tropical Medicine, London, UK (LSHTM); Radboud university medical center, Nijmegen, the Netherlands (RUMC)

PRINCIPAL INVESTIGATORS: Dr. B. Alfred Tiono (GRAS), Prof. Teun Bousema (LSHTM; RUMC), Prof. Chris Drakeley (LSHTM).

SPONSOR DETAILS: London School of Hygiene and Tropical Medicine, London, UK.

Compound ID: ______________

What is the purpose of this study?

Malaria is caused by parasites that are transmitted by mosquito bites and can be cured by early treatment with malaria drugs. When a human has had an infection for some time, the parasites in their body may be picked up by other mosquitoes when they bite, making that mosquito infectious to other humans. This is known as the cycle of transmission – malaria has to be transmitted from human to mosquito to human and so on, to survive. When a person has been infected with malaria a number of times they may be protected against some of the symptoms of the disease. When this protection has developed, the infected individual may not show signs of disease, but the parasite will still be present in the body, and may be capable of infecting mosquitoes.

Seasonal Malaria Chemoprevention (SMC) is one of the tools currently used to combat malaria in children. It involves repeated administrations of sulfadoxine-pyrimethamine plus amodiaquine (SPAQ) to children below the age of 5 years during the rainy season which is the peak transmission period in areas of seasonal malaria transmission. Whilst highly impactful in controlled research settings, the impact of SMC in terms of reducing infection prevalence is limited in real-life settings. We therefore want to do this research to understand what are the factors that are reducing the SMC effects in our population. We would also want to know whether extending SMC to all children below 10 years of age will be beneficial to them and moreover to the wider people in the village.

For this during this research we will compare three groups of population:

1) First group will include children under the age of 5 years receiving the SMC delivered by the local health district as per national guidelines

Moreno M, *et al. BMJ Open* 2024; 14:e081682. doi: 10.1136/bmjopen-2023-081682

2) In the second group children under age of 5 years will receive directly observed treatment for the full (three day) course of SMC.
3) Children in the third group will be under the age of 10 years. They will receive directly observed treatment for the full (three day) course of SMC.

What are the study procedures?
We aim to include 360 compounds from district of Saponé. From each compound, we will select up to 6 individuals including all the children aged below 10 years and over. This selection will be done at random. These selected study participants will be examined for signs of severe disease and other health conditions that may influence the ability to complete the study.
For all people who were selected and want to participate in the study, we will divide compounds in three arms. This division will be done at random (by chance). We will then provide to each child under 10 years an identification card and take pictures that will be attached to this card to help us easily identify them when they come to the health facility. With these cards, study participants have the right to free malaria-related medical care at the health facility. This free care is provided 24 hours per day, 7 days per week for a period of 6-7 months.
The adults will take part to cross sectional surveys on three occasions during which we will ask questions and collect blood sample by finger prick.
The compounds will be visited at least twice every month during the SMC round and after the round to administer study procedures to participants. We will explain in detail the procedures each participant will follow when we discuss the individual informed consent with potential participants.

Voluntary participation
You are free to decide whether you want your compound to be in the study or not. Your decision not to allow your compound to participate to the study will not affect the care your compound members receive at the local health facility any way. Even if you do agree for your compound to enter in the study, you can withdraw your compound from the study at any time without giving any reason.

What discomforts and risks is my compound exposed to?
There is no direct discomfort or risk for your compound to be in the study. You will receive regular home visits over the study period (6-7 months). These visits may disturb your family privacy. However, we will do our best to minimise this and our community health workers will be trained to use discretion to ensure your privacy.

What benefits can I expect from my compound's participation?
You will not receive any payment for your compound being part of the study.

What about the confidentiality of compound data?
The records concerning your compound's participation are to be used only for the purpose of this research project. The names of your compound members will not be used on any report resulting from this study. At the beginning of the study, we will give your compound a study identification number and this number will be used on the study documents. Any information obtained in connection with this study will be kept strictly confidential and under lock and key. Only senior research team members and authorized people such as ethics committee members will have access to your compound's records.

What if I have questions?

If you have any question concerning this study, do not hesitate to contact the Principal Investigator, Dr Alfred B. Tiono at [redacted] Results from the study will be communicated to your community. In case you want to contact an independent person, not related to the study, about the research study itself, you can contact the Chairman of Ethics Committee, Prof. Fla KOUETA at [redacted]

Moreno M, et al. BMJ Open 2024; 14:e081682. doi: 10.1136/bmjopen-2023-081682

Informed consent agreement

I, ____ (NAME OF HOUSEHOLD HEAD), having full capacity to consent to the participation of my compound in the study entitled **" INDIE_SMC Understanding and maximizing the community impact of antimalarial treatment "** conducted by principal investigator Dr. Alfred B. Tiono, acknowledge the implications of voluntary participation, the nature, duration and purpose, methods and means by which it is to be conducted. Harms and risks that can reasonably be expected to be encountered have been explained to me by ____ and are set out in the information document. I have had the opportunity to ask questions about this research and all questions have been answered to my satisfaction. If I have any further questions, I can contact Dr. Alfred B. Tiono (GRAS) at [redacted] I understand that I may at any time during the course of this study revoke my consent and withdraw my compound from the study without prejudice; however, compound members may be asked to undergo further examinations if, in the opinion of the physician, such examinations are necessary for their well-being.

I understand/do not understand the practical implications of this study (circle one)

I approve / disapprove of members of my compound being visited at home during the 6-7 months period of the study. (circle one)

I agree/disagree that my compound could take part in this study (circle one)

Name of Compound Head : ____

Signature : ______________________

Date : ______________________

Fingerprint if unable to sign

****

Name of the Independent Witness: ____

Signature : ___________________________ Date : _____________________

****

Name of person obtaining the consent: ____

Signature : ______________________________ Date : ____________________

Moreno M, *et al. BMJ Open* 2024; 14:e081682. doi: 10.1136/bmjopen-2023-081682

Participant parent/guardian of children in the study area information and Informed Consent Documents

TITLE OF STUDY: INDIE_SMC Understanding and maximizing the community impact of antimalarial treatment

INSTITUTIONS: Groupe de Recherche Action en Santé (GRAS); London School of Hygiene & Tropical Medicine, London, UK (LSHTM); Radboud university medical center, Nijmegen, the Netherlands (RUMC)

PRINCIPAL INVESTIGATORS: Dr. B. Alfred Tiono (GRAS), Prof. Teun Bousema (LSHTM; RUMC), Prof. Chris Drakeley (LSHTM).

SPONSOR DETAILS: London School of Hygiene and Tropical Medicine, London, UK.

Participant ID: ______________

What is the purpose of this study?

Malaria is caused by parasites that are transmitted by mosquito bites and can be cured by early treatment with malaria drugs. When a human has had an infection for some time, the parasites in their body may be picked up by other mosquitoes when they bite, making that mosquito infectious to other humans. This is known as the cycle of transmission – malaria has to be transmitted from human to mosquito to human and so on, to survive. When a person has been infected with malaria a number of times, they may be protected againts some of the symptoms of the disease. When this protection has developed, the infected individual may not show signs of disease, but the parasite will still be present in the body, and may be capable of infecting mosquitoes.

Seasonal Malaria Chemoprevention (SMC) is one of the tools currently used to combat malaria in children. It involves repeated administrations of sulfadoxine-pyrimethamine plus amodiaquine (SPAQ) to children below the age of 5 years during the rainy season which is the peak transmission period in areas of seasonal malaria transmission. Whilst highly impactful in controlled research settings, the impact of SMC in terms of reducing infection prevalence is limited in real-life settings. We therefore want to do this research to understand what are the factors that are hindering the SMC effects in our population. We would also want to know whether extending SMC to all children below 10 years of age will be beneficial to them and moreover to the wider people in the village.

For this during this research we will compare three groups of population:

1) First group will include children under the age of 5 years receiving the SMC delivered by the local health district as per national guidelines.
2) In the second group children under age of 5 years will receive directly observed treatment for the full (three day) course of SMC.

3) Children in the third group will be under the age of 10 years. They will receive directly observed treatment for the full (three day) course of SMC.

What are the study procedures?
We aim to include 2160 participants (1440 children and 720 adults) in this study.
If you allow your child/ward to be in the study, we will collect a blood sample by finger to assess whether he/she has the germs that cause malaria in his/her blood.
If your child/ward is in the first group, we will again visit him/her during the SMC rounds at four occasions to collect finger prick samples to check whether he/she has germs that cause malaria. During these visits, we will also ask you questions about his/her health status and how the SMC treatment was given. If your child/ward is in the second or third group, we will visit him/her at four occasions during the SMC round to make sure the treatment is correctly taken under supervision and to collect finger prick samples to check whether he/she has germs that cause malaria.

Whatever group your child/ward is in, after the last round of SMC we will collect finger prick samples one week later to assess the level of SMC drugs he/she has taken; this will be repeated at either three, four or five weeks after the round then once more at the study conclusion to check for the presence of germs that cause malaria.
In case your child/ward is found after the second SMC round to have parasites that could be transmitted to mosquitoes, we will collect a venous blood sample. The blood sample is used for further examination of malaria parasites and to determine whether the blood can infect mosquitoes. During this infectivity assessment, your child will not have direct contact with the mosquitoes. A small volume of blood is taken from the venous blood sample and offered to the mosquitoes in a glass dispenser.
During the participation of your child/ward to the study, the total volume of blood we will collect will not exceed 16 mL (approximately 3 teaspoons).

Voluntary participation
You are free to decide whether you want to allow your child/ward to be in the study or not. Your decision not to enrol your child/ward in the study will not affect the care you or your child/ward receive at the local health facility any way. Even if you do agree for your child/ward to be in the study, you can withdraw him/her from the study at any time without giving any reason.

What discomforts and risks is my child/ward exposed to?
Your child/ward may feel a little discomfort when the blood is taken and there may be a small bruise or bleeding on the finger or arm where the blood sample is taken. This is considered benign. We will use sterile equipment to take the blood sample and the small wound that may result from the procedure will be treated appropriately. The volume of blood is too small to affect the health of your child/ward and the blood will be quickly replaced by his/her body.

What benefits can I expect from my child/ward participation?
Your child/ward will receive standard antimalarial treatment and medical care for malaria and other non-chronic diseases free of charge during the study period (6 to 7 months).
Participants have free access to the study health facility during the period of their participation, at the clinician's discretion, antimalarials, antibiotics, and treatment for acute illnesses will be provided free of charge. If necessary, hospital care will be provided in the well-equipped wards in Saponé or in a more specialised hospital.

You will not get paid for your child/ward to be in the study. We will also not pay you when we visit your child/ward at home. However, you will get compensated whenever we ask you to bring your child to the local health facility for us to collect a blood sample for the mosquito feeding assay we have described to you before. You will receive two thousand CFA francs (2,000 FCFA) to compensate the time you lose for working. You will receive the amount at the end of the visit at the health facility.

What about the confidentiality of my child/ward personal data?
The records concerning your child/ward are to be used only for the purpose of this research project. Your child/ward name will not be used on any report resulting from this study. At the beginning of the study, we will give your child/ward a study identification number and this number will be used on the study documents. Any information obtained in connection with this study will be kept strictly confidential and under lock and key. Only senior members of the research team and authorized people such as ethical committee members will have access to your child/ward's records.

What happens to the samples?
The blood samples will be used to test for the germs that cause malaria and the SMC drug levels. Some of the samples will be stored in the freezer in our laboratories before they can be tested. We request your permission to ship some of your child/ward samples to laboratories outside the country for further tests. These countries include the United Kingdom and the Netherlands and possibly other countries. We will store your child/ward samples in the laboratory with a unique number and not with your child/ward name. Therefore, people not involved in the study will not be able to link the sample to your child/ward. We may share your child/ward test results with researchers at other organisations, but we will not give them your child/ward name, address, or any information that could identify your child/ward.

Most of these tests are conducted for research purposes only and cannot be reported back to you or your child/ward's doctor to guide your care.

We will also ask your permission to use the stored samples for future studies that may not be related to this study. These future studies may help find new ways to prevent malaria. If you agree, your child/ward samples may be stored for up to 10 years in Europe (UK and the Netherlands) and in Burkina Faso. After this study has ended, we will remove any means to link the sample to your child/ward's name. If you do not wish to have your child/ward's sample stored for future tests, your child/ward may still participate in our study. You can also change your mind at any time while the study is ongoing.

What about the study results?
This study is being done to help with scientific research and is not for medical diagnosis; therefore we will not provide individual results to you. If, during the course of the study, the study team learns information about your child/ward's health from the study procedures, the study team may discuss this information and your options with you.
A description of this clinical study will be available on https://clinicaltrials.gov/ registry. This web site will not include information that can identify your child/ward.

What if I have questions?
If you have any question concerning this study or concerning your child/ward's rights as a research study participant, do not hesitate to contact the Principal Investigator, Dr

Alfred B. Tiono [redacted] Results from the study will be communicated to your community when available.
In case you want to contact an independent person, not related to the study, about the research study itself, you can contact the Chairman of Ethics Committee, Prof. Fla KOUETA at [redacted].

Informed consent agreement

I, ____ (NAME PARENT/GUARDIAN), having full capacity to consent to the participation of my child/ward ___________________________________ (child/ward name) in the study entitled **" INDIE_SMC Understanding and maximizing the community impact of antimalarial treatment "** conducted by principal investigator Dr. Alfred B. Tiono, acknowledge the implications of voluntary participation, the nature, duration and purpose, methods and means by which it is to be conducted. The harms and risks that can reasonably be expected to be encountered have been explained to me by ____ and are set out in the informed consent form. I have had the opportunity to ask questions about this research and all questions have been answered to my satisfaction. If I have any further questions, I can contact Dr. Alfred B. Tiono (GRAS) at [redacted] I understand that I may at any time during this study revoke my consent and withdraw my child/ward from the study without prejudice; however, my child/ward may be asked to undergo further examinations if, in the opinion of the physician, such examinations are necessary for his/her well-being.

I understand/do not understand the practical implications of this study (circle one)

I agree/disagree that my child/ward should donate blood during the study for the mosquito feeding experiment (circle one)

I agree/disagree that my child/ward's samples be shipped out of the country for analysis related to the study (circle one)

I agree/disagree that my child/ward's samples be stored for future studies (circle one)

I agree/disagree that my child/ward should take part in this study (circle one)

Name of Parent/Guardian : ____

Signature : ______________________

Date : ______________________

Fingerprint if unable to sign

****

Name of the Independent Witness: ____

Signature : ___________________________ Date : _____________________

****

Name of person obtaining the consent: ____

Signature : ______________________________ Date : ____________________

Adult participant to the cross-sectional surveys information and Informed Consent Documents

TITLE OF STUDY: INDIE_SMC Understanding and maximizing the community impact of antimalarial treatment

INSTITUTIONS: Groupe de Recherche Action en Santé (GRAS); London School of Hygiene & Tropical Medicine, London, UK (LSHTM); Radboud university medical center, Nijmegen, the Netherlands (RUMC)

PRINCIPAL INVESTIGATORS: Dr. B. Alfred Tiono (GRAS), Prof. Teun Bousema (LSHTM; RUMC), Prof. Chris Drakeley (LSHTM).

SPONSOR DETAILS: London School of Hygiene and Tropical Medicine, London, UK.

Participant ID: ______________

What is the purpose of this study?
Malaria is caused by parasites that are transmitted by mosquito bites and can be cured by early treatment with malaria drugs. When a human has had an infection for some time, the parasites in their body may be picked up by other mosquitoes when they bite, making that mosquito infectious to other humans. This is known as the cycle of transmission – malaria has to be transmitted from human to mosquito to human and so on, to survive. When a person has been infected with malaria a number of times, they may be protected against some of the symptoms of the disease. When this protection has developed, the infected individual may not show signs of disease, but the parasite will still be present in the body, and may be capable of infecting mosquitoes.
Seasonal Malaria Chemoprevention (SMC) is one of the tools currently used to combat malaria in children. It involves repeated administrations of sulfadoxine-pyrimethamine plus amodiaquine (SPAQ) to children below the age of 5 years during the rainy season which is the peak transmission period in areas of seasonal malaria transmission. Whilst highly impactful in controlled research settings, the impact of SMC in terms of reducing infection prevalence is limited in real-life settings. We therefore want to do this research to understand what are the factors that are hindering the SMC effects in our population. We would also want to know whether extending SMC to all children below 10 years of age will be beneficial to them and moreover to the wider people in the village.
This is why although you are not taking SMC we want to do assessments on you to see if you indirectly benefited from the intervention.

What are the study procedures?
We aim to include at least 2160 participants (1440 children and 720 adults) in this study.
If you accept to be in the study, we will collect a blood sample by finger prick to assess whether you have the germs that cause malaria in your blood. The total volume will not exceed 4 mL (1 teaspoon).
Your participation will last for one day.

Voluntary participation
You are free to decide whether you want to be in the study or not. Your decision not to participate to the study will not affect the care you receive at the local health facility any way. Even if you do agree to be in the study, you can withdraw from the study at any time without giving any reason.

What discomforts and risks am I exposed to?
You may feel a little discomfort when the blood is taken and there may be a small bruise or bleeding on the finger or arm where the blood sample is taken. This is considered benign. We will use sterile equipment to take the blood sample and the small wound that may result from the procedure will be treated appropriately. The volume of blood is too small to affect your health and the blood will be quickly replaced by your body.

What benefits can I expect from my participation?
You will receive standard antimalarial treatment and medical care for malaria and other non-chronic diseases free of charge during your participation.
Participants have free access to the study health facility during the period of their participation (one day), at the clinician's discretion, antimalarials, antibiotics, and treatment for acute illnesses will be provided free of charge. If necessary, hospital care will be provided in the well-equipped wards in Saponé or in a more specialised hospital. You will not get paid to be in the study.

What about the confidentiality of my personal data?
The records concerning you are to be used only for the purpose of this research project. Your name will not be used on any report resulting from this study. At enrolment, we will give you a study identification number and this number will be used on the study documents. Any information obtained in connection with this study will be kept strictly confidential and under lock and key. Only senior members of the research team and authorized people such as ethical committee members will have access to your records.

What happens to the samples?
The blood samples will be used to test for the germs that cause malaria and the SMC drug levels. Some of the samples will be stored in the freezer in our laboratories before they can be tested. We request your permission to ship some of your samples to laboratories outside the country for further tests. These countries include the United Kingdom and the Netherlands and possibly other countries. We will store your samples in the laboratory with a unique number and not with your name. Therefore, people not involved in the study will not be able to link the sample to you. We may share your test results with researchers at other organisations, but we will not give them your name, address, or any information that could identify you.

Most of these tests are conducted for research purposes only and cannot be reported back to you or your doctor to guide your care.

We will also ask your permission to use the stored samples for future studies that may not be related to this study. These future studies may help find new ways to prevent malaria. If you agree, your samples may be stored for up to 10 years in Europe (UK and the Netherlands) and in Burkina Faso. After this study has ended, we will remove any means to link the sample to your name. If you do not wish to have your sample stored for future tests, you may still participate in our study. You can also change your mind at any time while the study is ongoing.

What about the study Results?
This study is being done to help with scientific research and is not for medical diagnosis; therefore we will not provide individual results to you. If, during the course of the study, the study team learns information about your health from the study procedures, the study team may discuss this information and your options with you.
A description of this clinical study will be available on https://clinicaltrials.gov/ registry. This web site will not include information that can identify you.

What if I have questions?
If you have any question concerning this study or concerning your rights as a research study participant, do not hesitate to contact the Principal Investigator, Dr Alfred B. Tiono at [redacted] Results from the study will be communicated to your community when available.
In case you want to contact an independent person, not related to the study, about the research study itself, you can contact the Chairman of Ethics Committee, Prof. Fla KOUETA at [redacted]

Moreno M, *et al. BMJ Open* 2024; 14:e081682. doi: 10.1136/bmjopen-2023-081682

Informed consent agreement

I, ____ (NAME OF THE PARTICIPANT), having full capacity to consent for my participation in the study entitled **" INDIE_SMC Understanding and maximizing the community impact of antimalarial treatment** " conducted by principal investigator Dr. Alfred B. Tiono, acknowledge the implications of voluntary participation, the nature, duration and purpose; methods and means by which it is to be conducted. The harms and risks that can reasonably be expected to be encountered have been explained to me by ____ and are set out in the informed consent form. I have had the opportunity to ask questions about this research and all questions have been answered to my satisfaction. If I have any further questions, I can contact Dr. Alfred B. Tiono (GRAS) at [redacted]. I understand that I may at any time during this study revoke my consent and withdraw from the study without prejudice; however, I may be asked to undergo further examinations if, in the opinion of the physician, such examinations are necessary for my well-being.

I understand/do not understand the practical implications of this study (circle one)

I agree/disagree to donate blood sample during the study (circle one)

I agree/disagree that my samples be shipped out of the country for analysis related to the study (circle one)

I agree/disagree that my samples be stored for future studies (circle one)

I agree/disagree to take part in this study (circle one)

Name of Participant: ____

Signature : ______________________

Date : ______________________

Fingerprint if unable to sign

****

Name of the Independent Witness: ____

Signature : ___________________________ Date : _____________________

****

Name of person obtaining the consent: ____

Signature : ______________________________ Date : ____________________

Moreno M, *et al. BMJ Open* 2024; 14:e081682. doi: 10.1136/bmjopen-2023-081682

Parent/Guardian of Minor participant to the cross-sectional surveys information and Informed Consent Documents

TITLE OF STUDY: INDIE_SMC Understanding and maximizing the community impact of antimalarial treatment

INSTITUTIONS: Groupe de Recherche Action en Santé (GRAS); London School of Hygiene & Tropical Medicine, London, UK (LSHTM); Radboud university medical center, Nijmegen, the Netherlands (RUMC)

PRINCIPAL INVESTIGATORS: Dr. B. Alfred Tiono (GRAS), Prof. Teun Bousema (LSHTM; RUMC), Prof. Chris Drakeley (LSHTM).

SPONSOR DETAILS: London School of Hygiene and Tropical Medicine, London, UK.

Participant ID: _______________

What is the purpose of this study?

Malaria is caused by parasites that are transmitted by mosquito bites and can be cured by early treatment with malaria drugs. When a human has had an infection for some time, the parasites in their body may be picked up by other mosquitoes when they bite, making that mosquito infectious to other humans. This is known as the cycle of transmission – malaria has to be transmitted from human to mosquito to human and so on, to survive. When a person has been infected with malaria a number of times, they may be protected against some of the symptoms of the disease. When this protection has developed, the infected individual may not show signs of disease, but the parasite will still be present in the body, and may be capable of infecting mosquitoes.

Seasonal Malaria Chemoprevention (SMC) is one of the tools currently used to combat malaria in children. It involves repeated administrations of sulfadoxine-pyrimethamine plus amodiaquine (SPAQ) to children below the age of 5 years during the rainy season which is the peak transmission period in areas of seasonal malaria transmission. Whilst highly impactful in controlled research settings, the impact of SMC in terms of reducing infection prevalence is limited in real-life settings. We therefore want to do this research to understand what are the factors that are hindering the SMC effects in our population. We would also want to know whether extending SMC to all children below 10 years of age will be beneficial to them and moreover to the wider people in the village.

This is why although you are not taking SMC we want to do assessments on you to see if you indirectly benefited from the intervention.

What are the study procedures?
We aim to include at least 2160 participants (1440 children and 720 adults) in this study.
If you accept your child/ward to be in the study, we will collect a blood sample by finger prick to assess whether your child/ward has the germs that cause malaria in your blood. The total volume will not exceed 4 mL (1 teaspoon).
Your child/ward's participation will last for one day.

Voluntary participation
You are free to decide whether you want your child/ward to be in the study or not. Your decision not to allow your child/ward to participate to the study will not affect the care you/ your child/ward receive at the local health facility any way. Even if you do agree for your child/ward to be in the study, you can withdraw him/her from the study at any time without giving any reason.

What discomforts and risks your child/ward is exposed to?
You child/ward may feel a little discomfort when the blood is taken and there may be a small bruise or bleeding on the finger or arm where the blood sample is taken. This is considered benign. We will use sterile equipment to take the blood sample and the small wound that may result from the procedure will be treated appropriately. The volume of blood is too small to affect your child/ward health and the blood will be quickly replaced by his/her body.

What benefits can I expect from my child/ward participation?
Your child/ward will receive standard antimalarial treatment and medical care for malaria and other non-chronic diseases free of charge during your participation.
Participants have free access to the study health facility during the period of their participation (one day), at the clinician's discretion, antimalarials, antibiotics, and treatment for acute illnesses will be provided free of charge. If necessary, hospital care will be provided in the well-equipped wards in Saponé or in a more specialised hospital. You will not get paid for your child/ward to be in the study.

What about the confidentiality of my child/ward personal data?
The records concerning your child/ward are to be used only for the purpose of this research project. Your child/ward name will not be used on any report resulting from this study. At enrolment, we will give your child/ward a study identification number and this number will be used on the study documents. Any information obtained in connection with this study will be kept strictly confidential and under lock and key. Only senior members of the research team and authorized people such as ethical committee members will have access to your records.

What happens to the samples?
The blood samples will be used to test for the germs that cause malaria and the SMC drug levels. Some of the samples will be stored in the freezer in our laboratories before they can be tested. We request your permission to ship some of your samples to laboratories outside the country for further tests. These countries include the United Kingdom and the Netherlands and possibly other countries. We will store your child/ward samples in the laboratory with a unique number and not with your child/ward name. Therefore, people not involved in the study will not be able to link the sample to your

child/ward. We may share your child/ward test results with researchers at other organisations, but we will not give them your child/ward name, address, or any information that could identify your child/ward.

Most of these tests are conducted for research purposes only and cannot be reported back to you or your child/ward doctor to guide your care.

We will also ask your permission to use the stored samples for future studies that may not be related to this study. These future studies may help find new ways to prevent malaria. If you agree, your samples may be stored for up to 10 years in Europe (UK and the Netherlands) and in Burkina Faso. After this study has ended, we will remove any means to link the sample to your child/ward name. If you do not wish to have your child/ward sample stored for future tests, you may still allow your child/ward to participate in our study. You can also change your mind at any time while the study is ongoing.

What about the study Results?

This study is being done to help with scientific research and is not for medical diagnosis; therefore we will not provide individual results to you. If, during the course of the study, the study team learns information about your child/ward health from the study procedures, the study team may discuss this information and your options with you.
A description of this clinical study will be available on https://clinicaltrials.gov/ registry. This web site will not include information that can identify your child/ward.

What if I have questions?

If you have any question concerning this study or concerning your child/ward rights as a research study participant, do not hesitate to contact the Principal Investigator, Dr Alfred B. Tiono at [redacted] Results from the study will be communicated to your community when available.
In case you want to contact an independent person, not related to the study, about the research study itself, you can contact the Chairman of Ethics Committee, Prof. Fla KOUETA [redacted]

Moreno M, et al. BMJ Open 2024; 14:e081682. doi: 10.1136/bmjopen-2023-081682

Informed consent agreement

I, ____ (NAME OF THE PARENT/GUARDIAN), having full capacity to consent for my child/ward's____ participation in the study entitled " **INDIE_SMC Understanding and maximizing the community impact of antimalarial treatment** " conducted by principal investigator Dr. Alfred B. Tiono, acknowledge the implications of voluntary participation, the nature, duration and purpose; methods and means by which it is to be conducted. The harms and risks that can reasonably be expected to be encountered have been explained to me by ____ and are set out in the informed consent form. I have had the opportunity to ask questions about this research and all questions have been answered to my satisfaction. If I have any further questions, I can contact Dr. Alfred B. Tiono (GRAS) at [redacted] I understand that I may at any time during this study revoke my consent and withdraw my child/ward from the study without prejudice; however, I may be asked that my child/ward undergoes further examinations if, in the opinion of the physician, such examinations are necessary for his/her well-being.

I understand/do not understand the practical implications of this study (circle one)

I agree/disagree for my child/ward to donate blood sample during the study (circle one)

I agree/disagree that my child/ward samples be shipped out of the country for analysis related to the study (circle one)

I agree/disagree that my child/ward samples be stored for future studies (circle one)

I agree/disagree for my child/ward to take part in this study (circle one)

Name of Parent/Guardian: ____

Signature : _____________________

Date : _____________________

Fingerprint if unable to sign

****

Name of the Independent Witness: ____

Signature : __________________________ Date : ____________________

****

Name of person obtaining the consent: ____

Signature : _____________________________ Date : ___________________

Moreno M, *et al. BMJ Open* 2024; 14:e081682. doi: 10.1136/bmjopen-2023-081682
