# Supplementary material for: Understanding and maximising the community impact of seasonal malaria chemoprevention in Burkina Faso (INDIE-SMC): study protocol for a cluster randomised evaluation trial
Source: BMJ Open. 2024 Mar 12;14(3):e081682. doi: 10.1136/bmjopen-2023-081682 (PMC10936478; doi:10.1136/bmjopen-2023-081682)
Supplement: Supplementary data [file bmjopen-2023-081682supp002.pdf]

Supplementary data

Title: Understanding and maximizing the community impact of seasonal malaria chemoprevention in Burkina Faso (INDIE-SMC): study protocol for a cluster randomized evaluation trial

Authors: Moreno M[1*], Barry A[2,3*], Gmeiner M[4*], Yaro JB[2], Sermé SS[2], Byrne I[1], Ramjith J[4], Ouedraogo A[2], Soulama I[2], Grignard L[1], Soremekun S[1], Koele S[4], ter Heine R[4], Ouedraogo AZ[2], Sawadogo J[2], Sanogo E[2], Ouedraogo IN[2], Hien D[2], Sirima SB[2], Bradley J[1], Bousema T[4], Drakeley C[1*], Tiono AB[2*§]

1 Department of Infection Biology, London School of Hygiene and Tropical Medicine, London, UK

2 Groupe de Recherche Action Santé, Ouagadougou, Burkina Faso

3 Secretariat Permanent pour l'élimination du Paludisme, Ministère de la Santé et de l'hygiène publique, Ouagadougou, Burkina Faso

4 Department of Medical Microbiology, Radboud University Medical Centre, Nijmegen, The Netherlands

Table S1. SPAQ dosing according to Burkina Faso MoH. *NA: not applicable.*

Age	SMC Drugs	Day 1	Day 2	Day 3
**3-11 months**	SP : 262.5 mg S, fixed dose fixe with P	1 Tablet	*NA*	*NA*
	AQ : 76.5 mg	1 Tablet	1 Tablet	1 Tablet
**12-59 months**	SP : 525 mg S	1 Tablet	*NA*	*NA*
	AQ : 153 mg	1 Tablet	1 Tablet	1 Tablet
**5-9 years**	SP : 525 mg S	1 Tablet	*NA*	*NA*
	SP : 262.5 mg S	1 Tablet	*NA*	*NA*
	AQ : 153 mg	1 Tablet	1 Tablet	1 Tablet
	AQ : 76.5 mg	1 Tablet	1 Tablet	1 Tablet
