# Supplementary material for: Understanding and maximising the community impact of seasonal malaria chemoprevention in Burkina Faso (INDIE-SMC): study protocol for a cluster randomised evaluation trial
Source: BMJ Open. 2024 Mar 12;14(3):e081682. doi: 10.1136/bmjopen-2023-081682 (PMC10936478; doi:10.1136/bmjopen-2023-081682)
Supplement: Supplementary data [file bmjopen-2023-081682supp003.pdf]

Statistical Analysis Plan

Understanding and maximizing the community impact of antimalarial treatment (INDIE-SMC)

Version 1.0

Date: 30 October 2023

Moreno M, *et al. BMJ Open* 2024; 14:e081682. doi: 10.1136/bmjopen-2023-081682

Table of Contents

1 Study details 3
2 Study design 4
2.1 Introduction 4
2.2 Study rationale 4
2.3 Design & intervention 5
3 Randomization 6
4 Study objectives 6
5 Study endpoints 7
5.1 Primary endpoint 7
5.2 Secondary endpoint 7
5.3 Exploratory endpoints 8
6 Study population 8
7 Sample size considerations 8
8 Data entry 10
9. Coding 10
10 Statistical methods 11
10.1 Demographics 11
10.2 Study flow 11
10.3 Effectiveness of SMC and SMC efficacy decay 11
10.4 Human infectious reservoir 12
10.5 Drug resistance 12
10.6 Social barriers and enablers 12
10.7 Analysing data and reporting results 13
11 Conduct of analyses 16
11.1 Programming 16
12 Reporting 16
13 Document history 16
14 Signature page 17
15 References 18

Moreno M, *et al. BMJ Open* 2024; 14:e081682. doi: 10.1136/bmjopen-2023-081682

1 Study details

Protocol Title
Understanding and maximizing the community impact of antimalarial treatment (INDIE-SMC)

Study code
INDIE-SMC

Trial Register Identifier
NCT05878366

SAP Authors
Markus Gmeiner, Marta Moreno, Teun Bousema, John Bradley, Chris Drakeley

Investigator
Alfred B. Tiono

Groupe de Recherche Action Santé (GRAS), Burkina Faso

Sponsor (representative)
Chris Drakeley

London School of Hygiene and Tropical Medicine (LSHTM), United Kingdom

Moreno M, *et al. BMJ Open* 2024; 14:e081682. doi: 10.1136/bmjopen-2023-081682

Table 1: Abbreviations

Abbreviation	Full form
AQ	Amodiaquine
CRF	Case Report Form
DEAQ	Desethylamodiaquine
DOT	Directly Observed Treatment
FGD	Focus Group Discussion
GRAS	Groupe de Recherche Action Santé
IDI	In-Depth Interview
IQR	Interquartile Range
KII	Key Informant Interview
LLIN	Long Lasting Insecticide Net
LSHTM	London School of Hygiene and Tropical Medicine
MoH	Ministry of Health
ODK	Open Data Kit
qRT PCR	Quantitative Real-Time Polymerase Chain Reaction
SAP	Statistical Analysis Plan
SMC	Seasonal Malaria Chemoprevention
SPAQ	Sulfadoxine-Pyrimethamine plus Amodiaquine

2 Study design

2.1 Introduction

The Statistical Analysis Plan (SAP) describes the statistical analyses of data obtained in the INDIE-SMC study. It is a controlled document and may be updated until the final analysis is performed.

2.2 Study rationale

Seasonal Malaria Chemoprevention (SMC) currently involves repeated administrations of sulfadoxine-pyrimethamine plus amodiaquine (SPAQ) to children below the age of 5 years during the peak transmission season in areas of seasonal malaria transmission. Whilst highly impactful in reducing the burden of *P. falciparum* malaria in controlled research

Moreno M, *et al. BMJ Open* 2024; 14:e081682. doi: 10.1136/bmjopen-2023-081682

settings, the impact of SMC in terms of reducing infection prevalence is limited in real-life settings.

It is currently unclear whether failure of SMC with SPAQ to prevent malaria parasitaemia is associated with

i) imperfect adherence;

ii) parasite resistance to the drug components;

iii) active drug concentrations that are achieved in SMC participants;

iv) consistent exposure to reinfection.

It is also unclear whether extending the age-range targeted by SMC will reduce the infectious reservoir for transmission to a level where community-benefits may be expected. It is further currently unclear what factors determine (cascades in) efficacy decay and whether SMC coverage and uptake is equitable or affected by, for example, gender of children in the target age group. Quantifying each of these elements should contribute to understanding the effectiveness of SMC in programmatic settings.

2.3 Design & intervention

The study is designed to evaluate SMC effectiveness and consists of three interventional study arms, with the first arm receiving an intervention that is implemented as part of routine control.

Study arm 1:	SMC in children under the age of 5 years, implemented by the Ministry of Health (MoH) without directly observed treatment for the full course of SMC
Study arm 2:	SMC in children under the age of 5 years, with directly observed treatment for the full course of SMC
Study arm 3:	SMC in children under the age of 10 years, with directly observed treatment for the full course of SMC

SMC consists of SP and AQ administered daily for three days. SP is given on the first SMC day only while AQ is given daily over three days. In total 4 rounds of SMC will be conducted during the peak malaria transmission season from July to October, separated by approx. 30 days.

Each study arm contains 62 clusters; each cluster consists of 3 compounds where each compound has at least one child aged 3-59 months and one child aged 5-9 years. (186 compounds per arm; 558 compounds in total). Differences in SMC coverage and efficacy will be determined in the SMC-targeted age groups. In contrast, all age groups will take part in the evaluation of the study to assess the impact of the intervention on the infectious reservoir in the community and compare parasite carriage in targeted and untargeted populations. To detect possible behavioural contamination and differences in SMC uptake or adherence in study arm 1 compared to what would be observed if there would be no

study team present, a single cross-sectional survey 7 days after the last (4th) round of SMC coverage is assessed outside the study area (2-5 kilometres from the nearest enrolled study compound) by questionnaire and samples to determine plasma levels of SMC drugs and parasite carriage by qPCR. Plasma levels of SMCR drugs are used as the most definitive evidence of differences in SMC uptake/adherence.

Scheduled surveys and scheduled or unscheduled sampling timepoints will take place in each arm; these surveys are designed and timed for several outcome measures and include:

i) a survey prior to first SMC roll-out (performed in all compounds).

ii) mid-study surveys 3, 4 and 5 weeks after the last SMC roll out (each compound will participate in one of these surveys).

iii) end-of-study survey up to 10 weeks after last SMC roll out (performed in all compounds).

iv) sampling for drug metabolite assessment shortly after the last SMC round (sample collection in all compounds in the study arms and outside-study control area).

v) sampling for transmission assay on first day of SMC round 2 to 4 (sampling in all compounds; selection from individuals aged 5-9 years).

vi) passive case detection in all four health facilities that are accessed by the study population throughout the study period (from enrolment until the end of follow-up).

3 Randomization

The unit of randomization will be the cluster; each cluster comprises of 3 compounds. Clusters are randomly allocated to one of the three study arms by a computer generated algorithm and stratified by village. Stratification by village was to account for spatial differences in malaria exposure, distance from health facilities, and treatment seeking behavior. In total, 185 clusters were randomized.

4 Study objectives

The specific study objectives are to:

i) compare SMC effectiveness in children aged 3-59 months as implemented by the national malaria control program and SMC implemented in a research context where all doses are directly observed.

ii) Evaluate the effectiveness of SMC to reduce malaria infection prevalence in 5-9 year olds

Moreno M, *et al. BMJ Open* 2024; 14:e081682. doi: 10.1136/bmjopen-2023-081682

iii) quantify the infectious reservoir and the contribution of different age groups to transmission with conventional SMC (<5 years) and extended SMC (<10 years)

iv) determine the impact of drug resistance (by molecular typing of *dhps*, *dhfr, pfcrt* and *pfmdr1*) and drug absorption on SMC efficacy

v) understand social barriers and enablers interfering with SMC efficacy and how SMC uptake is related to health equity with special attention to gender inequalities.

vi) quantify SMC efficacy decay under programmatic conditions and key drivers of this decay.

5 Study endpoints

5.1 Primary endpoint

- Parasite prevalence by quantitative PCR (qPCR) at the end of the transmission season in age groups targeted by seasonal malaria chemoprevention. [end-of-study survey].

This endpoint will be compared between arms 1 and 2 (in children aged 3-59 months) and arms 2 and arm 3 (in children aged 5 years-9 years). [end-of-study survey].

5.2 Secondary endpoint

- Parasite prevalence by qPCR at the end of the transmission season in all age groups (between arm comparison). [end-of-study survey].
- Parasite prevalence by qPCR at the end of the transmission season compared between arms 1 and combined arms 2 + 3. [end-of-study survey].
- Parasite prevalence by microscopy prior to SMC rounds 2, 3 and 4 in SMC-targeted age groups (between arm comparison). [at SMC-delivery].
- Rate of re-infection with *P. falciparum* at weeks 3, 4 and 5 after the last round of SMC, assessed in SMC-targeted age groups (between arm comparison). [mid-study surveys].
- Gametocyte prevalence by qRT-PCR at weeks 3, 4 and 5 after the last round of SMC, assessed in SMC-targeted age groups (between arm comparison). [mid-study surveys].
- Gametocyte prevalence by qRT-PCR at the end of the transmission season in all age groups (between arm comparison). [end-of-study survey].
- Gametocyte prevalence by qRT-PCR at the end of the transmission season in age groups targeted by SMC (comparison between arm1 and 2 (in children aged 3-59 months) and arms 2 and arm 3 (in children aged 5 years-9 years). [end-of-study survey].
- Plasma levels of AQ and desethylamodiaquine (DEAQ) after the 4th round of SMC in children aged 3 months-9 years (between arm comparison) [post-SMC round 4].
- Clinical malaria incidence captured during passive-case detection (between arm comparison) [[post-SMC round 4]

5.3 Exploratory endpoints

- Infectivity to mosquitoes, defined as the percentage of infected mosquitoes, in relation to gametocyte density and plasma drug levels of AQ and DEAQ (across arms assessment) [during and shortly after SMC rounds 2-4].
- Size and age-distribution of the infectious reservoir for malaria, defined as the likelihood that a mosquito becomes infected with malaria parasites after feeding on a population member (between arm comparison) [during the study].
- Prevalence of drug resistance markers in infected children aged 3 months-9 years assessed post each round of SMC (between arm comparison) [during the entire study].
- Description of perceived social barriers to SMC uptake [during the entire study].
- Quantification of SMC efficacy decay under programmatic conditions [during the entire study].

6 Study population

The study population will be all members of 555 compounds (185 clusters of 3 compounds) in Saponé Health District (Burkina Faso). Clusters are randomized over 3 study arms. Compound members of all ages will be included in study procedures (e.g. evaluation of the intervention) but only children will be targeted with the intervention. Based on census data, and recent studies in the same study area, an average of 1.5 children aged <5 years and 1.5 child 5-9 years per compound is expected (4.5 and 4.5 per cluster, respectively).

In addition, 120 randomly selected compounds outside of the study area are included for a single survey after the last round of SMC.

7 Sample size considerations

Evaluable study clusters will comprise 3 compounds and have between 4-5 children below 5 years of age (eligible for standard SMC) and similar numbers of children aged 5-10 years (eligible for SMC in study arm 3). We assume ~40% parasite prevalence detected by PCR at the end of the transmission season in children aged 5-10 without SMC and 16% parasite prevalence in children aged <5 years under programmatic SMC (based on recently completed work in the same area Collins, Ouedraogo et al. in prep). When assuming a reduction to 25% parasite prevalence in children aged 5-10 years when included in SMC, a sample size of 55 clusters per arm (x3 compounds per cluster and an estimated total 6 children in this age group per cluster) will achieve 87% power to detect this difference at an alpha of 0.05 and a coefficient of variation of 0.3 (data from INDIE 1a; LSHTM EC #14724; Centre National de Recherche et de Formation sur le Paludisme institutional review board (Deliberation N° 2018/000002/MS/SG/CNRFP/CIB; Burkina Faso national medical ethics committee (Deliberation N° 2018-01-010)). This sample size will also allow the detection of a reduction of parasite prevalence in directly observed SMC to 7% as

compared to 16% under programmatic SMC, in children under 5 years of age. The total number of compounds per arm has been increased to 62 to mitigate the effects of non-compliance and drop-outs and also for logistical reasons, such whole villages are included. We will thus enrol a total of 186 clusters over 3 arms in a ratio of 1:1:1 with 558 compounds in total. We anticipate that this will include a minimum of 720 children below 5 years of age (2 per compound, based on recent census data) and 720 children aged 5-9 years (1 per compound as per inclusion criterion).

For assessments of the human infectious reservoir, the intervention study is too large in scope (i.e. 558 compounds, compared to 180 in the previous INDIE project) and the time-window for transmission assays is too narrow (i.e. transmission assays are ideally done within weeks after SCM administration) to assess a direct impact of SMC on gametocyte infectivity with a conventionally powered comparison between arms. Thus a combination of methodologies (quantification of the association between gametocyte density and infectivity in the absence of treatment; imputed mosquito transmission from gametocyte density) are used (i, ii) It was further shown that direct comparisons of the association between gametocyte density and mosquito infection rates in mosquito feeding assays can demonstrate whether imputation is indeed acceptable across populations (i*)* or whether there are subpopulations with a different association (for example due to gametocyte-sterilizing effects of antimalarials (iii)). These insights will be used to perform mosquito feeding assays on gametocyte positive individuals with and without prior SMC and compare mosquito infection rates for a given gametocyte density (iii). Since the hypothesis is that SPAQ will permanently sterilize gametocytes, any reduced infectivity of gametocytes will remain apparent even after drug levels have waned (i.e. the gametocytes are permanently damaged by drug exposure) (iv). A possible sterilizing effect of SPAQ can be assessed by preferentially recruiting children who are gametocyte positive at the start of SMC and invite them for mosquito feeding post SMC. This will be done in children aged 5-9 years only who carry the highest parasite densities and highest gametocyte prevalence/density (i); this also avoids phlebotomy from the youngest children. A median gametocyte density of ~14 gametocytes/µL (IQR 1.8-44.2) as previously observed post-SMC (v, vi) is anticipated. Whilst the exact number of experiments that can be conducted will be dictated by mosquito husbandry, simulations indicate that when recruiting 100 children who received SPAQ (arm 3) and 100 children who did not (arms 1 & 2) and dissecting 40 mosquitoes per experiment, we would have >90% power to detect a 70% reduction in infectivity (vii).

An exact sample size justification for the single cross-sectional survey in the area outside the study-targeted population is complicated; this survey is performed to give a broad indication of the potential influence the presence of our study team may have on the operational delivery of SMC in arm 1. It is not part of any main comparisons. Estimates of adherence are based on questionnaire data, supported by measurements of DEAQ drug levels in plasma. In arm 2 and 3 (Directly Observed Treatment (DOT); by design full adherence), the median level of DEAQ at day 4 following the last dose of AQ is expected to be 550 ng/mL (IQR 350-850)[14]. Based on a large assessment of reported adherence and measured AQ/DEAQ plasma levels following SMC in Niger (viii), we anticipate that <50% of children show complete adherence in arms 1 and the control village and >20% of the children in these populations may fail to take any dose. If we conservatively assume that

this results in a reduction of median DEAQ levels to <400ng/mL (IQR 200-700), we can estimate the power to detect this difference when enrolling 120 children in the SMC-targeted age range in the control area. For this, we assume that these DEAQ levels stem from right-skewed (log-normal) distributions; giving median log DEAQ concentrations of 6.31 (IQR 5.86-6.75) for DOT and 5.99 (IQR 5.30-6.55) for non-DOT. If it is further assumed that the distribution of the logged values is normally distributed, we can estimate the standard deviation through the IQR as IQR/1.349, giving standard deviations of 0.93 and 0.66, respectively. Using a sample size of 120 per group, and a Welch's two sample t-test for testing the differences between the log DEAQ concentrations, we estimate a power of 86.3% to detect these differences. Based on these assumptions, we are confident that sampling 120 children <5 years of age will allow meaningful assessments. As indicated above, this sample size estimation is not relevant for any of the main comparisons in the study but merely used to ensure informative sampling and avoid exposing an unnecessarily large population to the small discomfort of a single finger prick sample. We will thus aim to determine plasma levels of at least 120 children <5 years in arm 1;120 children <5 years from arms 2 and 3 combined and 120 children from the control area.

Drug plasma levels are also relevant for other comparisons (e.g. the impact on transmission efficiency in mosquito feeding assays, the role in explaining clinical malaria episodes and asymptomatic parasite carriage after SMC. For these comparisons, samples may be selected in a case-control manner.

8 Data entry

All study data from the baseline survey up until the end-of-study survey are entered into an electronic data capture system (ODK) by the investigators and delegated personnel, either directly (in which case these are themselves considered Source data) and/or from a designated primary Source, e.g. paper Case Report Forms (CRF), Blood Smear read out results.

9. Coding

Data coding for not standardized data is not applied.

All data in the database is pseudonymized. A study participant identification link log is available at the investigators' site.

10 Statistical methods

10.1 Demographics

Baseline characteristics (age, gender) will be tabulated. Categorical variables will be presented as count and percentage. Numerical variables will be summarized as median, minimum and maximum. GPS data and maps will be used to present location of compounds and clusters.

10.2 Study flow

A study flow chart (e.g. CONSORT flow chart) will be used to present number of volunteers approached for enrolment, enrolled, withdrawn and followed until completion of the study.

10.3 Effectiveness of SMC and SMC efficacy decay

Assessing effectiveness of SMC requires collection of data on:

- *Coverage (proportion of children reached by the four SMC rounds)*
- *Health Care worker's compliance (proportion of children receiving first dose of SMC under DOT)*
- *Caregiver's compliance (proportion of children receiving correct SMC regimen)*
- *Drug levels (proportion of children with adequate drug level)*

<u>SMC treatment coverage</u>

SMC coverage will be assessed through a combination of direct observation of treatment and pharmacological assessments. In arm 1 Directly Observed Treatment (DOT) is applied only on the first day of each SMC round. In arm 2 and 3 DOT is applied for the full three-day course of SMC. DOT includes observation of drug administration and a 15 minutes post-administration observation period. In arms 2 and 3, coverage will be expressed as percentage of the target population receiving at least one dose of SMC, at least two doses of SMC and the full 3-day course of SMC.

The adherence for all study participants will be evaluated based on observed amodiaquine (AQ) and desethylamodiayiune (DEAQ) plasma concentrations 4 days after the last dose of SMC should have been taken (day 7 since the start of the SMC round), as previously described by Ding J. et al. (2020 (viii)).

Moreno M, *et al. BMJ Open* 2024; 14:e081682. doi: 10.1136/bmjopen-2023-081682

Qualitative data on compliance

See section 10.6

Biomarkers:

Serological analysis of blood samples will be conducted using bead based assays to assess presence of biomarkers for correlates of infection (i.e. proteins associated with inflammation or parasite density (HRP2)). These biomarkers, assessed at individual level will be related to parasite positivity, transmission efficiency etc. as part of exploratory analyses.

10.4 Human infectious reservoir

The human infectious reservoir will be assessed by a combination of direct measurements of transmissibility (assessed in a selection of parasite-positive children aged 5-9 years) and modeling/transmission imputation as described in detail in section 10.7 endpoint 12.

10.5 Drug resistance

Nucleic acids will be extracted with commercial kits [Qiagen] from blood samples collected on filter paper. Three dual-labelled probes were designed to detect three crt genotypes at codons 72–76 (encoding Cys-Val-Met-Asn-Lys [CVMNK], CVIET, and SVMNT).

10.6 Social barriers and enablers

The qualitative component of the study is designed to understand potential factors that influence SMC uptake and effectiveness.
Throughout the study, data on compliance to SMC treatment guidelines from caregiver and healthcare worker perspectives are collected. Whether first dose was directly observed and whether the full treatment course was administered to the child after each round on SMC is documented. After SMC round 4 a cross-sectional survey will be carried out. For this, individual interviews of caregivers of children targeted or not by the current SMC strategy will be conducted.

Further, within each of the study arms, between 6-8 individuals will be recruited for focus group discussions (FGDs). Community-based field workers will be asked to purposively select individuals for the FGD. Preference will be given for eligible participants that are active in community life and events, and therefore would have had greater exposure to views and experiences of other community members. The selection will pay much attention to gender balance. The FGD will be closed to outside observers, limited only to consenting participants and the fieldworkers conducting the session. Two field workers will conduct the FGD. One field worker will lead the discussion and the other will take notes. The discussion will be recorded for transcription. A semi-structured discussion guide will be used to guide the

discussion. The discussion will last approximately 60 minutes. The recording of the discussion will be used to create a discussion transcript for data analysis.

Key informant interviews (KIIs) and in-depth interviews (IDIs) will be carried out. For the KIIs, participants will be recruited through purposive sampling to include stakeholders within the health system involved in SMC implementation.
The IDIs will be conducted among 5-10 key informants per each of the study arms. These individuals will also be purposively selected using community-based field workers. The aim is to include community leaders, professionals, or residents who have first-hand knowledge about the community. The interviewer will use a semi-structured interview guide to guide the discussion that will last approximately 60 minutes. The interview will be recorded for transcription. The recording of the interview will be used to create a discussion transcript for data analysis.

10.7 Analysing data and reporting results

When analysing data based on age, participants' age at time of the first round of SMC is used. If a child received SMC because he/she was <5 years at the time of SMC but turns 5 prior to the last survey, the age at the moment of SMC round 1 is used since this dictated the intervention.

Primary endpoint

1. *Parasite prevalence by quantitative PCR (qPCR) at the end of the transmission season in age groups targeted by seasonal malaria chemoprevention. [end-of-study survey].*

This endpoint will be compared between arms 1 and 2 (in children aged 3-59 months) and arms 2 and arm 3 (in children aged 5 years--9 years). Parasite prevalence by qPCR will be used as binary variable with all parasite densities above 100 parasites/mL (0.1 parasite/μL) being classified as parasite positive. Parasite prevalence is compared between arms by logistic regression; models will account for clustering by including cluster as a random intercept and village as a fixed effect.

Secondary endpoints

2. *Parasite prevalence by qPCR at the end of the transmission season in all age groups (between arm comparison). [end-of-study survey].*

This outcome will be compared between arms as described above for outcome 1.

3. *Parasite prevalence by qPCR at the end of the transmission season compared between arms 1 and combined arms 2 + 3. [end-of-study survey].*

This outcome will be compared between arms as described above for outcome 1.

Moreno M, *et al. BMJ Open* 2024; 14:e081682. doi: 10.1136/bmjopen-2023-081682

4. *Parasite prevalence by microscopy prior to SMC rounds 2, 3 and 4 in SMC-targeted age groups (between arm comparison). [at SMC-delivery].*

This outcome will be compared between arms as described above for outcome 1.

5. *Rate of re-infection with P. falciparum at weeks 3, 4 and 5 after the last round of SMC, assessed in SMC-targeted age groups (between arm comparison). [mid-study surveys].*

This outcome will be presented as prevalence estimates with 95% confidence intervals for each time-point. It will be compared between arms as described above for outcome 1.

6. *Gametocyte prevalence by qRT-PCR at weeks 3, 4 and 5 after the last round of SMC, assessed in SMC-targeted age groups (between arm comparison). [mid-study surveys].*

This outcome will be presented as prevalence estimates with 95% confidence intervals for each time-point and study arm separately. It will be compared between arms as described above for outcome 1.

7. *Gametocyte prevalence by qRT-PCR at the end of the transmission season in all age groups (between arm comparison). [end-of-study survey].*

This outcome will be presented as prevalence estimate with 95% confidence interval for each study arm separately. It will be compared between arms as described above for outcome 1.

8. *Gametocyte prevalence by qRT-PCR at the end of the transmission season in age groups targeted by SMC (comparison between arm1 and 2 (in children aged 3-59 months) and arms 2 and arm 3 (in children aged 5 years-9 years). [end-of-study survey].*

This outcome will be presented as prevalence estimates with 95% confidence intervals for each time-point and study arm separately. It will be compared between arms as described above for outcome 1.

9. *Plasma levels of AQ and desethylamodiaquine (DEAQ) after the 4th round of SMC in children aged 3 months-9 years (between arm comparison) [post-SMC round 4].*

A published non-linear mixed-effects pharmacokinetic model will be evaluated for the ability to describe AQ and DEAQ pharmacokinetics in the study population (viii). All quantifiable plasma concentrations available will be used for model evaluation using gold-standard methods. Visual predictive checks and goodness-of-fit plots will be made to assess model performance for participants in which dosing history is observed through DOT. For each study participant 2000 full concentration-time profiles will be simulated, considering participant age and weight, assuming full treatment adherence. Model-predicted concentrations at the PK sampling time will be determined for each simulation. The optimal cut-off percentile discriminating between adherence and non-adherence was previously determined to be the 20th percentile of the individual simulated concentration-time profiles (71% sensitivity, 80% sensitivity) (viii).

Participants with observed plasma concentrations below the individual cut-off percentile will be scored as non-adherent. Coverage will be expressed as percentage of participants scored as non-adherent per treatment arm. Additional analyses will include the percentage of participants with/without any measurable AQ or DEAQ plasma concentrations.

10. *Incidence of clinical malaria captured during passive-case detection (between arm comparison) [[post-SMC round 4]*

Passive case detection data will be analysed by Poisson regression. The outcome will be the number of cases in each compound. Comparisons will be made between arms 1 and 2 in children aged 3-59 months and between arms 2 and 3 in children aged 5-9 years. There will be a random effect for compound and an offset of the number of children.

Exploratory endpoints

11. *Infectivity to mosquitoes, defined as the percentage of infected mosquitoes, in relation to gametocyte density and plasma drug levels of AQ and DEAQ (across arms assessment) [during and shortly after SMC rounds 2-4].*

The association between proportion of infected mosquitoes and gametocyte density will determined using generalized linear models assuming a binomial distribution with a log-link for participants with and without detectable AQ/DEAQ plasma concentrations. In addition, infectivity will be compared between individuals with/without AQ/DEAQ plasma levels using a previously published statistical model (ix). In case of low infectivity, the nonparametric van Elteren's test, an extension of the Wilcoxon rank sum test that compares groups in a stratified manner will be used (iii)where we compare infectivity between groups after stratifying in gametocyte density categories. Infectivity is then compared within different bins to test for differences between arms, accounting for gametocyte density. Additional analyses will take into consideration AQ/DEAQ concentrations at continuous scale (rather than simple detectability).

12. *Size and age-distribution of the infectious reservoir for malaria, defined as the likelihood that a mosquito becomes infected with malaria parasites after feeding on a population member (between arm comparison) [during the study].*

The size and shape of the infectious reservoir will be estimated using a combination of directly observed mosquito infection events and imputed transmissibility (i, ii). This imputed transmissibility will use gametocyte density (measured for all relevant timepoints) as starting point. The association between proportion of infected mosquitoes and gametocyte density will determined using generalized linear models assuming a binomial distribution with a log-link. If drug levels do not influence infectivity (see 8), a single modelled association will be used. If drug levels do have a statistically significant impact on transmission potential, all estimates of the infectious reservoir within one month of SMC will take study arm into account. For the infectious reservoir at the final end of study survey, no impact of SMC drugs on transmission efficiency is assumed. The

contribution of different age groups (younger than 5 years, 5–15 years, 16 years and older) to the infectious reservoir will be estimated using all data (including parasite-negative observations where zero transmission is assumed)

13. *Prevalence of drug resistance markers in infected children aged 3 months-9 years assessed post each round of SMC (between arm comparison) [during the entire study].*
14. *Description of perceived social barriers to SMC uptake [during the entire study].*
15. *Quantification of SMC efficacy decay under programmatic conditions [during the entire study].*

11 Conduct of analyses

11.1 Programming

Analysis will be performed using STATA software and R. Pharmacokinetic model evaluation and adherence simulations will be performed using NONMEM 7.5 with Pirana 2.9.9 as interface.

12 Reporting

Ethics committees at LSHTM and in Burkina Faso will receive annual reports on study progress. Final results will be uploaded to clinicaltrials.gov upon publishing of these results in a peer-reviewed journal.

13 Document history

Version	Author	Description of change	Date
1.0	Markus Gmeiner	New document	30 OCT 2023

Moreno M, *et al. BMJ Open* 2024; 14:e081682. doi: 10.1136/bmjopen-2023-081682

14 Signature page

Date:	
Name (*Principal Investigator)*:	*Alfred B. Tiono*
Signature:	

Date:	
Name (*Sponsor Representative)*:	*Chris Drakeley*
Signature:	

Moreno M, *et al. BMJ Open* 2024; 14:e081682. doi: 10.1136/bmjopen-2023-081682

15 References

[i] Andolina, C., Rek, J. C., Briggs, J., Okoth, J., Musiime, A., Ramjith, J., Teyssier, N., Conrad, M., Nankabirwa, J. I., Lanke, K., Rodriguez-Barraquer, I., Meerstein-Kessel, L., Arinaitwe, E., Olwoch, P., Rosenthal, P. J., Kamya, M. R., Dorsey, G., Greenhouse, B., Drakeley, C., Staedke, S. G., … Bousema, T. (2021). Sources of persistent malaria transmission in a setting with effective malaria control in eastern Uganda: a longitudinal, observational cohort study. *The Lancet. Infectious diseases*, *21*(11), 1568–1578. https://doi.org/10.1016/S1473-3099(21)00072-4

[ii] Rek, J., Blanken, S. L., Okoth, J., Ayo, D., Onyige, I., Musasizi, E., Ramjith, J., Andolina, C., Lanke, K., Arinaitwe, E., Olwoch, P., Collins, K. A., Kamya, M. R., Dorsey, G., Drakeley, C., Staedke, S. G., Bousema, T., & Conrad, M. D. (2022). Asymptomatic School-Aged Children Are Important Drivers of Malaria Transmission in a High Endemicity Setting in Uganda. *The Journal of infectious diseases*, *226*(4), 708–713. https://doi.org/10.1093/infdis/jiac169

[iii] Bradley, J., Soumaré, H. M., Mahamar, A., Diawara, H., Roh, M., Delves, M., Drakeley, C., Churcher, T. S., Dicko, A., Gosling, R., & Bousema, T. (2019). Transmission-blocking Effects of Primaquine and Methylene Blue Suggest Plasmodium falciparum Gametocyte Sterilization Rather Than Effects on Sex Ratio. *Clinical infectious diseases : an official publication of the Infectious Diseases Society of America*, *69*(8), 1436–1439. https://doi.org/10.1093/cid/ciz134

[iv] Alkema, M., Reuling, I. J., de Jong, G. M., Lanke, K., Coffeng, L. E., van Gemert, G. J., van de Vegte-Bolmer, M., de Mast, Q., van Crevel, R., Ivinson, K., Ockenhouse, C. F., McCarthy, J. S., Sauerwein, R., Collins, K. A., & Bousema, T. (2021). A Randomized Clinical Trial to Compare Plasmodium falciparum Gametocytemia and Infectivity After Blood-Stage or Mosquito Bite-Induced Controlled Malaria Infection. *The Journal of infectious diseases*, *224*(7), 1257–1265. https://doi.org/10.1093/infdis/jiaa157

[v] Dicko, A., Roh, M. E., Diawara, H., Mahamar, A., Soumare, H. M., Lanke, K., Bradley, J., Sanogo, K., Kone, D. T., Diarra, K., Keita, S., Issiaka, D., Traore, S. F., McCulloch, C., Stone, W. J. R., Hwang, J., Müller, O., Brown, J. M., Srinivasan, V., Drakeley, C., … Bousema, T. (2018). Efficacy and safety of primaquine and methylene blue for prevention of Plasmodium falciparum transmission in Mali: a phase 2, single-blind, randomised controlled trial. *The Lancet. Infectious diseases*, *18*(6), 627–639. https://doi.org/10.1016/S1473-3099(18)30044-6

[vi] Stone, W., Mahamar, A., Smit, M. J., Sanogo, K., Sinaba, Y., Niambele, S. M., Sacko, A., Keita, S., Dicko, O. M., Diallo, M., Maguiraga, S. O., Samake, S., Attaher, O., Lanke, K., Ter Heine, R., Bradley, J., McCall, M. B. B., Issiaka, D., Traore, S. F., Bousema, T., … Dicko, A. (2022). Single low-dose tafenoquine combined with dihydroartemisinin-piperaquine to reduce Plasmodium falciparum transmission in Ouelessebougou, Mali: a phase 2, single-blind, randomised clinical trial. *The Lancet. Microbe*, *3*(5), e336–e347. https://doi.org/10.1016/S2666-5247(21)00356-6

[vii] Ramjith, J., Alkema, M., Bradley, J., Dicko, A., Drakeley, C., Stone, W., & Bousema, T. (2022). Quantifying Reductions in *Plasmodium falciparum* Infectivity to Mosquitos: A Sample Size Calculator to Inform Clinical Trials on Transmission-Reducing Interventions. *Frontiers in immunology*, *13*, 899615. https://doi.org/10.3389/fimmu.2022.899615

[viii] Ding, J., Coldiron, M. E., Assao, B., Guindo, O., Blessborn, D., Winterberg, M., Grais, R. F., Koscalova, A., Langendorf, C., & Tarning, J. (2020). Adherence and Population Pharmacokinetic Properties of

Amodiaquine When Used for Seasonal Malaria Chemoprevention in African Children. *Clinical pharmacology and therapeutics*, *107*(5), 1179–1188. https://doi.org/10.1002/cpt.1707

[ix] Bradley, J., Stone, W., Da, D. F., Morlais, I., Dicko, A., Cohuet, A., Guelbeogo, W. M., Mahamar, A., Nsango, S., Soumaré, H. M., Diawara, H., Lanke, K., Graumans, W., Siebelink-Stoter, R., van de Vegte-Bolmer, M., Chen, I., Tiono, A., Gonçalves, B. P., Gosling, R., Sauerwein, R. W., … Bousema, T. (2018). Predicting the likelihood and intensity of mosquito infection from sex specific *Plasmodium falciparum* gametocyte density. *eLife*, *7*, e34463. https://doi.org/10.7554/eLife.34463
