# Supplementary material for: Understanding and maximising the community impact of seasonal malaria chemoprevention in Burkina Faso (INDIE-SMC): study protocol for a cluster randomised evaluation trial
Source: BMJ Open. 2024 Mar 12;14(3):e081682. doi: 10.1136/bmjopen-2023-081682 (PMC10936478; doi:10.1136/bmjopen-2023-081682)
Supplement: Author's manuscript [file bmjopen-2023-081682.draft_revisions.pdf]

BMJ Open is committed to open peer review. As part of this commitment we make the peer review history of every article we publish publicly available.

When an article is published we post the peer reviewers' comments and the authors' responses online. We also post the versions of the paper that were used during peer review. These are the versions that the peer review comments apply to.

The versions of the paper that follow are the versions that were submitted during the peer review process. They are not the versions of record or the final published versions. They should not be cited or distributed as the published version of this manuscript.

BMJ Open is an open access journal and the full, final, typeset and author-corrected version of record of the manuscript is available on our site with no access controls, subscription charges or pay-per-view fees (http://bmjopen.bmj.com).

If you have any questions on BMJ Open's open peer review process please email info.bmjopen@bmj.com

BMJ Open

Understanding and maximizing the community impact of seasonal malaria chemoprevention in Burkina Faso (INDIE-SMC): study protocol for a cluster randomized evaluation trial

Journal:	*BMJ Open*
Manuscript ID	bmjopen-2023-081682
Article Type:	Protocol
Date Submitted by the Author:	03-Nov-2023
Complete List of Authors:	Moreno, Marta; London School of Hygiene & Tropical Medicine, Department of Infection Biology<br>Barry, Aissata; Groupe de Recherche Action en Santé<br>Gmeiner, Markus; Radboud University Nijmegen Department of Medical Microbiology<br>Yaro, Jean ; Groupe de Recherche Action en Santé<br>Sermé, Samuel ; Groupe de Recherche Action en Santé<br>Byrne, Isabel; London School of Hygiene & Tropical Medicine, Department of Infection Biology<br>Ramjith, Jordache; Radboud University Nijmegen Department of Medical Microbiology<br>Ouedraogo, Alphonse; Groupe de Recherche Action en Santé<br>Soulama, Issiaka; Groupe de Recherche Action en Santé<br>Grignard, Lynn; London School of Hygiene & Tropical Medicine, Department of Infection Biology<br>Soremekun, Seyi; London School of Hygiene & Tropical Medicine, Department of Infection Biology<br>Koele, S; Radboud University Nijmegen Department of Medical Microbiology<br>ter Heine, R; Radboudumc, Pharmacy<br>Ouedraogo, Amidou; Groupe de Recherche Action en Santé<br>Sawadogo, Jean; Groupe de Recherche Action en Santé<br>Sanogo, Edith; Groupe de Recherche Action en Santé<br>Ouedraogo, Issa; Groupe de Recherche Action en Santé<br>Hien, Denise; Groupe de Recherche Action en Santé<br>Sirima, Sodiomon Bienvenu; Groupe de Recherche Action en Santé<br>Bradley, John; London School of Hygiene & Tropical Medicine, Department of Infection Biology<br>Bousema, Teun; Radboud University Nijmegen Department of Medical Microbiology<br>Drakeley, Chris; London School of Hygiene & Tropical Medicine, Department of Infection Biology<br>Tiono, Alfred B. ; Groupe de Recherche Action en Santé
Keywords:	Malaria, EPIDEMIOLOGIC STUDIES, Tropical medicine < INFECTIOUS DISEASES

BMJ Open

Title: Understanding and maximizing the community impact of seasonal malaria chemoprevention in Burkina Faso (INDIE-SMC): study protocol for a cluster randomized evaluation trial

Authors: Moreno M[1*], Barry A[2,3*], Gmeiner M[4*], Yaro JB[2], Sermé SS[2], Byrne I[1], Ramjith J[4], Ouedraogo A[2], Soulama I[2], Grignard L[1], Soremekun S[1], Koele S[4], ter Heine R[4], Ouedraogo AZ[2], Sawadogo J[2], Sanogo E[2], Ouedraogo IN[2], Hien D[2], Sirima SB[2], Bradley J[1], Bousema T[4], Drakeley C[1*], Tiono AB[2*§]

1 Department of Infection Biology, London School of Hygiene and Tropical Medicine, London, UK

2 Groupe de Recherche Action Santé, Ouagadougou, Burkina Faso

3 Secretariat Permanent pour l'élimination du Paludisme, Ministère de la Santé et de l'hygiène publique, Ouagadougou, Burkina Faso

4 Department of Medical Microbiology, Radboud University Medical Centre, Nijmegen, The Netherlands

* Contributed equally

[§]Corresponding author:

Alfred B Tiono

Email: a.tiono@gras.bf

Address: Groupe de Recherche Action en Santé (GRAS)

06 BP 10248 Ouagadougou 06, Burkina Faso

Tél: +226 25 35 56 90/73

Email addresses:

MM: Marta.moreno@lshtm.ac.uk

AB: a.barry@gras.bf

MG: markus.gmeiner@radboudumc.nl

JBY: j.yaro@gras.bf

SSS: s.serme@gras.bf

IB: isabel.byrne@lshtm.ac.uk

JR: jordache.ramjith@radboudumc.nl

AO: a.ouedraogo@gras.bf

IS: i.soulama@gras.bf

LG: lynn.grignard@lshtm.ac.uk

For peer review only - http://bmjopen.bmj.com/site/about/guidelines.xhtml

SS: seyi.soremekun@lshtm.ac.uk

SK: simon.koele@radboudumc.nl

RH: r.terheine@radboudumc.nl

AZO: oz.amidou@gras.bf

JS: j.sawadogo@gras.bf

ES: e.sanogo@gras.bf

INO: i.ouedraogo@gras.bf

DH: d.hien@gras.bf

SBS: s.sirima@gras.bf

JB: John.Bradley@lshtm.ac.uk

TB: Teun.Bousema@radboudumc.nl

CD: Chris.drakeley@lshtm.ac.uk

ABT: a.tiono@gras.bf

For peer review only

Abstract

Introduction Seasonal Malaria Chemoprevention (SMC) involves repeated administrations of sulfadoxine-pyrimethamine plus amodiaquine (SPAQ) to children below the age of 5 years during the peak transmission season in areas of seasonal malaria transmission. Whilst highly impactful in reducing *Plasmodium falciparum* malaria burden in controlled research settings, the impact of SMC on infection prevalence is moderate in real-life settings. Remains unclear what drivers of SMC coverage are involved and the importance of parasite drug resistance, imperfect adherence, poor drug absorption and re-infections remain unexplored. Recently, the World Health Organization (WHO) widened the scope for SMC to target all vulnerable populations. The Ministry of Health (MoH) in Burkina Faso is considering extending SMC to children below 10 years old; the impact of SMC on clinical incidence and parasite prevalence in this population is unknown. Due to the relevance of this age group for onward malaria transmission, it is important to quantify the impact of SMC on the human infectious reservoir for malaria.

Methods and analysis We will perform a cluster-randomized trial in Saponé Health District, Burkina Faso, with three study arms comprising 62 clusters of 3 households/compounds (185 compounds per arm): 1) SMC in children under 5 years old, implemented by the MoH without directly observed treatment (DOT) for the full course of SMC; 2) SMC in children under 5 years old, with DOT for the full course of SMC; 3) SMC in children under 10 years old, with DOT for the full course of SMC. The primary endpoint is parasite prevalence at the end of the malaria transmission season, secondary endpoints include the impact of SMC on clinical incidence, gametocyte carriage and potential for onward parasite transmission to mosquitoes. Factors affecting SMC uptake and treatment adherence, drug concentrations, parasite resistance markers and transmission of parasites to mosquitoes will be determined.

Trial registration number

NCT05878366

Keywords: malaria, Seasonal malaria chemoprevention, sulfadoxine-pyrimethamine, amodiaquine,

Plasmodium falciparum

For peer review only - http://bmjopen.bmj.com/site/about/guidelines.xhtml

Article summary

Strengths and limitations of this study

- Engagement with National Malaria Control Program (NMCP) in Burkina Faso. This study is a direct response to NMCP's strategic plan 2021-2025. Outcomes of this study shall guide SMC implementation in 2024/2025.
- First time quantification of the effect of SMC on the human infectious reservoir for malaria.
- Measurements of drug plasma concentrations as direct measurements of the effective doses of SMC drugs that are achieved.
- The study is conducted in a single setting; whilst the implementation of SMC in the control arm is not directly observed, the presence of a study team might influence SMC uptake and this risk is mitigated by including an additional control area.

For peer review only

For peer review only - http://bmjopen.bmj.com/site/about/guidelines.xhtml

Introduction

a. Background

Efforts to reduce the global incidence of malaria are failing; there were an estimated 247 million cases of malaria worldwide in 2021, up from 230 million in 2015(1). With the threat that control efforts are further compromised by insecticide resistance and parasite resistance to first-line drugs, there is a clear need for interventions that reduce the clinical burden of malaria and onward parasite transmission(2).

One recent intervention that has the potential to profoundly reduce the incidence of (severe) clinical disease is seasonal malaria chemoprevention (SMC)(3). SMC provides antimalarials to the most vulnerable populations during the peak of malaria transmission season with the aim to reduce disease burden in this high-risk population. To date, SMC has been administered to millions of African children(4, 5). Although most commonly given to children below the age of 5 years, SMC is recommended for all children in age groups that are at high risk of severe malaria(6). The recent 2022 WHO guidelines offer increased flexibility in defining the specific number of monthly cycles and the target age group. In Burkina Faso, SMC is currently administered over 4 rounds to all children aged 3-59 months in July, August, September, and October. These SMC rounds cover the peak of the malaria transmission season that follows seasonal rains that typically peak in July-September. Several countries in the Sahel region, including Burkina Faso, are considering extending the target age range of SMC to all children under the age of 10 years.

Whilst SMC is highly efficacious in research settings, reducing morbidity by up to 60%(5, 7), effect sizes may be lower when SMC is rolled out programmatically (5, 8, 9). A cascade in efficacy decay is regularly observed for efficacious interventions; for SMC this efficacy decay may be related to coverage, compliance (at multiple levels), drug absorption and parasite resistance to SMC drugs (**Figure 1).** A large study that assessed plasma levels of SMC drugs in children in Niger reported evidence for incomplete adherence or drug absorption in >80% of children[9]. The choice of drug regimen and parasite resistance to drug components may also play important roles. SMC with the conventional drug combination of sulfadoxine-pyrimethamine plus amodiaquine (SPAQ) is not always effective at clearing parasites: up to 46% of children under the age of 5 years may carry parasites at the end of the transmission season(10) (Collins *et al*. *submitted*). Recently longitudinal studies in Burkina Faso and The Gambia observed that a large proportion of SMC-targeted children either remained *P. falciparum* infected or became re-infected shortly after receiving SMC with SPAQ (Collins *et al*. *submitted*; (11)).

Parasite resistance to the SPAQ antimalarial combination will almost certainly play a role in defining its anti-infection effects. Resistance to SP is strongly associated with the presence and number of point mutations in dihydropteroate synthase (dhps; e.g. mutations 437Gly and 540Glu) and dihydrofolate reductase (dhfr; e.g. 51Ile, 59Arg, and 108Asn) genes (12, 13). The prevalence of these core mutations and the number of additional mutations in dhps and dhfr are rising across Africa. In East-Africa, quintuple mutations in dhfr-dhps are common although a single study suggests that SMC with SPAQ retains protective efficacy (14). Compared to resistance markers for SP, the molecular mechanisms underlying AQ resistance are less well understood and molecular markers for AQ have limited predictive value.

AQ is metabolically activated, primarily by Cytochrome P450 isoenzyme 2C8 (CYP2C8), into desethylamodiaquine (DEAQ). The T_{max} for AQ is around 2 days (Interquartile Range (IQR) 1-3 days)

For peer review only - http://bmjopen.bmj.com/site/about/guidelines.xhtml

and drug concentrations wane rapidly between days 3 and 7. DEAQ concentrations peak ~2 days after AQ and the terminal half-life of DEAQ (~196.4 h) is approximately four-fold longer than that for AQ (15). AQ resistance associates with variant haplotypes in chloroquine transporter (crt; e.g. 72Cys-73Val-74Ile-75Glu-76Thr) and multidrug resistance gene 1 (mdr1; e.g. encoding Tyr at codons 86, 184, and 1246) (16) but better markers may become available.

In addition to imperfect efficacy against (incident) asexual-stage parasites, SPAQ may also be unable to prevent parasite transmission shortly after treatment. Gametocytes typically persist for several weeks after SPAQ (17)[,16] with conflicting findings on gametocyte infectivity after SP treatment. *In vitro* and *in vivo* studies have demonstrated considerable gametocyte sterilization by SP where gametocytes are detectable but no longer (as) infectious[(18),(19)]. However, clinical trials in naturally infected gametocyte carriers demonstrated that SPAQ is ineffective in clearing gametocytes and individuals may remain infectious to mosquitoes for several weeks after treatment ([(20)] Mahamar *et al. submitted*). Adding single low-dose primaquine rapidly prevented transmission(20).

With SMC being deployed at community level, this intervention has the potential to reduce community transmission and thereby reduce the force of infection experienced by the general population. Recent work directly assessing the infectivity of parasite carriers to mosquitoes identified school-age children (aged 5-15 years) as important reservoirs of infection(21-24). This population is less likely to become symptomatic upon infection and supports high parasite densities and transmissible gametocytes (21-24). Younger children are comparatively less important because their infections are more likely to receive treatment that prevents chronic parasite carriage and due to their small body size and high bednet coverage, mosquito exposure can be relatively low (25). In contrast, school-age children are heavily exposed, currently under-targeted with conventional interventions (e.g. have lowest bednet coverage) and carry the highest densities of gametocytes (21)[,](26). Whilst less at risk of severe clinical disease, also school-age children may suffer from consequences of parasite carriage that are often overlooked, including haemolysis, increased susceptibility to bacterial infections (27) and reduced cognitive function (26); also this age group would thus likely benefit from interventions that reduce this parasite carriage. Extending the target age range for SMC from all children below 5 years of age to all children below 10 years of age will thus increase the population experiencing direct personal benefits from treatment and may also potentially reduce community-wide transmission by covering a large proportion of the human infectious reservoir for malaria.

Recent observations lead to questions related to the reasons for the continued high infection rates in the SMC-targeted population and the potential impact of SMC when the age range is extended to populations that are important to fuelling transmission to the wider community.

b. Rationale

It is currently unclear whether failure of SMC with SPAQ to prevent parasitaemia is associated with i) imperfect adherence; ii) parasite resistance to the drug components, iii) active drug concentrations that are achieved in SMC participants, and/or iv) consistent exposure to reinfection. It is also unclear whether extending the targeted age-range will reduce the infectious reservoir for transmission to a level where community-benefits may be expected. Lastly, factors that determine (cascades in) efficacy decay are currently poorly understood. Quantifying each of these elements should contribute to understanding the effectiveness of SMC in programmatic settings.

For peer review only

For peer review only - http://bmjopen.bmj.com/site/about/guidelines.xhtml

c. Objectives

This study aims to collect detailed information on parasite carriage, gametocyte carriage and infectivity in children targeted and untargeted by SMC in Burkina Faso. The impact of expanding SMC to include children up to 10 years of age on reducing the human infectious reservoir and factors driving imperfect anti-parasite or anti-gametocyte effects of SMC will be assessed. The infectious reservoir will be examined using a combination of gametocyte density estimates in the entire population and mosquito membrane feeding assays in a selection of participants.

Our specific aims are to:

i. Compare SMC effectiveness in children aged 3-59 months in preventing clinical malaria and asymptomatic parasite carriage when implemented by the NMCP or when implemented in a research context where all doses are directly observed.
ii. Evaluate the effectiveness of SMC to reduce malaria infection prevalence in 5–9-year-olds.
iii. Quantify the infectious reservoir and the contribution of different age groups to transmission with conventional SMC (<5 years) and extended SMC (<10 years).
iv. Determine the impact of drug resistance (by molecular typing of *dhps*, *dhfr, pfcrt* and *pfmdr1*) and drug absorption on SMC efficacy.
v. Understand social barriers and enablers interfering with SMC efficacy and how SMC uptake is related to health equity with special attention to gender inequalities.
vi. Quantify SMC efficacy decay under programmatic conditions and key drivers of this decay.

Methods and analyses

a. Trial Design:

The study is designed to evaluate SMC effectiveness and consists of three study arms, with the first arm receiving an intervention that is implemented as part of routine control. This study involves an operational evaluation of a modified existing intervention delivered in independent clusters. The project and its implementation are prepared in direct interaction with the Burkina Faso MoH and NMCP to tailor data collection to local needs. There will be three study arms:

Study arm 1 (control): SMC in children under the age of 5 years, implemented by the MoH; the first dose is given under supervision but there is no directly observed treatment (DOT) for the full course of SMC.
Study arm 2 (intervention): SMC in children under the age of 5 years, with DOT for the full course of SMC.
Study arm 3 (intervention): SMC in children under the age of 10 years, with DOT for the full course of SMC.

SMC consists of SP and AQ administered daily for three days(28). SP is given on the first SMC day only while AQ is given daily over three days. In total 4 rounds of SMC are conducted during the peak malaria transmission season from July to October, separated by approximately 30 days, concurrently with programmatic interventions in study arms 2 and 3.

Each study arm contains 61 to 62 clusters; each cluster consists of 3 compounds where each compound has at least one child aged 3-59 months and one child aged 5-9 years (185 compounds per

For peer review only - http://bmjopen.bmj.com/site/about/guidelines.xhtml

arm; 555 compounds in total) (**Figure 2).** All age groups will take part in the evaluation of the study to assess the impact of the intervention on the infectious reservoir in the community and compare parasite carriage in targeted and untargeted populations. To detect possible behavioural contamination and differences in SMC uptake or adherence in study arm 1 compared to what would be observed if there would be no study team present, a single cross-sectional survey will take place 7 days after the last (4th) round of SMC outside the study area (2-5 kilometres from the nearest enrolled study compound). SMC coverage is assessed by questionnaire and sample collection to determine plasma levels of SMC drugs and parasite carriage.

This protocol was developed following SPIRIT reporting guidelines(29).

b. Endpoints:

The primary endpoint is parasite prevalence by quantitative Polymerase Chain Reaction (qPCR) at the end of the malaria transmission season in age groups targeted by SMC. This endpoint will be compared between study arms 1 and 2 (in children aged 3-59 months) and arms 2 and arm 3 (in children aged 5 years-9 years). Additional secondary and exploratory endpoints will be assessed (**Table 1**).

Table 1. Study primary, secondary and exploratory endpoints.

Primary endpoint

- Parasite prevalence by qPCR at the end of the transmission season in age groups targeted by seasonal malaria chemoprevention.

Secondary endpoints

- Parasite prevalence by qPCR at the end of the transmission season in all age groups (between arm comparison)
- Parasite prevalence by microscopy prior to SMC rounds 2, 3 and 4 in SMC-targeted age groups (between arm comparison)
- Rate of re-infection with *P. falciparum* at weeks 3, 4 and 5 after the last round of SMC, assessed in SMC-targeted age groups *(*between arm comparison*)*
- Gametocyte prevalence by qRT-PCR at weeks 3, 4 and 5 after the last round of SMC, assessed in SMC-targeted age groups (between arm comparison)
- Gametocyte prevalence by qRT-PCR at the end of the transmission season in all age groups (between arm comparison)
- Gametocyte prevalence by qRT-PCR at the end of the transmission season in age groups targeted by SMC (comparison between arms 1 and 2 (in children aged 3-59 months) and arms 2 and 3 (in children aged 5-9 years))
- Plasma levels of AQ and DEAQ after the 4th round of SMC in children aged 3 months-9 years (between arm comparison and comparison with control area)

For peer review only - http://bmjopen.bmj.com/site/about/guidelines.xhtml

- Incidence of clinical malaria captured during passive-case detection (between arm comparison)

Exploratory endpoints

- Infectivity to mosquitoes, defined as the percentage of infected mosquitoes, in relation to gametocyte density and plasma drug levels of AQ and DEAQ (across arms assessment)
- Size and age-distribution of the infectious reservoir for malaria, defined as the likelihood that a mosquito becomes infected with malaria parasites after feeding on a population member (between arm comparison)
- Prevalence of drug resistance markers in infected children aged 3 months-9 years assessed post each round of SMC (between arm comparison)
- Description of perceived social barriers to SMC uptake
- Quantification of SMC efficacy decay under programmatic conditions

c. Study sites:

The study site is in Saponé Health District (45 km south-west of Ouagadougou) and the study will benefit from historical records on malaria burden in this area and research infrastructure that was established in previous joint research projects(22, 30, 31) . Up-to-date census data will be used prior to the study to select eligible compounds for enrolment in the Saponé local health areas.

d. Study population:

The study population will be all members of 555 compounds (61 to 62 clusters per study arm; each cluster consisting of 3 compounds) in Saponé Health District, Burkina Faso. Clusters are randomized over 3 study arms. Compound members of all ages will be included in study procedures (e.g. evaluation of the intervention) but only children will be targeted with the intervention. Based on census data, and recent studies in the same study area, we expect an average of 1.5 children aged <5 years and 1.5 children aged 5-9 years per compound (4.5 children aged <5 years and 4.5 children aged 5-9 years per cluster, respectively).

e. Inclusion and exclusion criteria:

The inclusion and exclusion criteria (**Table 2**) for the evaluation trial are broad to ensure generalizability of the collected data and results.

For peer review only - http://bmjopen.bmj.com/site/about/guidelines.xhtml

Table 2: Inclusion and exclusion criteria for the evaluation trial and for the qualitative study on SMC acceptability and uptake

**Compounds are eligible for participation in the trial if**	there are at least 1 child aged <5 years and 1 child aged 5-9 years permanent residents of the compound and willing to participate.
	residents are willing to provide repeated blood samples up to a total volume of 16mL over an 8-month period (maximum of 4mL for children aged <5 years).
	the eligible individual or caregiver(s) provide informed consent and additional assent is provided from minor participants aged 12-19 years.
**Household members are excluded from participation in the trial in case of**	any (chronic) illness that requires continuous care.
	current participation in malaria vaccine or drug trials or participation in vaccine trials in the last 2 years.
	ineligibility for SPAQ administration according to NMCP guidelines (only for children).
**Community members are eligible for participation in the qualitative study if they are**	a resident of a household participating in the trial.
	caregiver of a child eligible to SMC.
	20 years of age or older.
**Health care workers are eligible for participation in the qualitative study if they are**	serving as a health care worker delivering SMC intervention.
	serving as health staff in the study zone.
**Community members are excluded from participation in the qualitative study if they are**	not residing in the household during SMC preceding the qualitative survey.

For peer review only

f. Recruitment:

Following community meetings where study objectives and procedures are explained study teams visit individual households to explain study objectives and procedures for a second time - this time to the head of household and all household members of assenting or consenting age. Study information is presented orally and print outs in French and/or a local language are provided. The voluntary nature of study participation and the right to withdraw at any time are emphasised. It is explained that refusal to participate will not affect access to SMC or any other medical treatment. All household members are encouraged to ask questions prior to signing informed consent (aged >19 years) or assent (aged 12-19 years).

g. Sample size:

Clusters

Evaluable study clusters will comprise 3 compounds and have between 4-5 children below 5 years of age (eligible for conventional SMC in all study arms) and similar numbers of children aged 5-9 years (eligible for extended SMC in arm 3). We assume ~40% parasite prevalence detected by qPCR at the end of the transmission season in children aged 5-9 without SMC and 16% parasite prevalence in children aged <5 years under programmatic SMC (Collins *et al*. *submitted*). When assuming a reduction to 25% parasite prevalence in children aged 5-9 years when included in SMC, a sample size of 55 clusters per arm (x3 compounds per cluster and an estimated total 6 children in this age group per cluster) will achieve 87% power to detect this difference at an alpha of 0.05 and a coefficient of variation of 0.3 (Collins *et al*. *submitted*). This sample size will give over 80% power to detect a reduction of parasite prevalence in directly observed SMC to 7% as compared to 16% under programmatic SMC, in children aged <5 years. The total number of compounds per arm has been increased to 62 to mitigate the effects of non-compliance and drop-outs and for logistical reasons, such entire villages are included. We will thus enrol a total of 185 clusters over 3 arms in a ratio of 1:1:1 with 555 compounds in total. We anticipate that this will include a minimum of 720 children aged <5 years and 720 children aged 5-9 years (based on recent census data).

Human infectious reservoir

For assessments of the human infectious reservoir, we use a combination of direct measurements of transmission by mosquito feeding assays and imputation of transmissibility based on gametocyte density(23). The reason for a partial reliance on imputation is that the intervention study is too large in scope and the time-window for mosquito feeding experiments is too narrow (i.e. mosquito feeding is ideally done within weeks after SMC) to assess a direct impact of SMC on gametocyte infectivity with a conventionally powered comparison between arms. We will thus use a combination of methodologies where we quantify the association between gametocyte density and infectivity in the absence of treatment, demonstrating consistency across settings(21, 22, 32) and impute mosquito transmission from gametocyte density (21, 23). Direct comparisons of the association between gametocyte density and mosquito infection rates in mosquito feeding assays can demonstrate whether imputation is indeed acceptable across populations(21) or whether there are subpopulations with a different association (for example due to gametocyte-sterilizing effects of antimalarials)(33). Based on these insights direct mosquito feeding assays (DMFAs) will be performed on gametocyte positive individuals with and without prior SMC and mosquito infection rates for a given gametocyte density be compared (33). Since we hypothesize that SPAQ may permanently sterilize gametocytes, any reduced infectivity of gametocytes will remain apparent even after drug levels have waned (i.e. the gametocytes are permanently damaged by drug exposure)(18). We can efficiently assess this possible sterilizing effect of SPAQ by preferentially recruiting children who are gametocyte positive at the start of SMC and invite them for mosquito feeding post SMC. This will be done in children aged 5-9 years only who carry the highest parasite and gametocyte densities; this also avoids phlebotomy from the youngest children. We anticipate a median gametocyte density of ~14 gametocytes/µL (IQR 1.8-44.2) as observed post-treatment(20, 34). Whilst the number of feeding assays will be informed by feasibility (e.g. mosquito husbandry), we provide an indication of study power. When recruiting 100 children who received SPAQ (arm 3) and 100 children who did not (arms 1 and 2) and dissecting

For peer review only - http://bmjopen.bmj.com/site/about/guidelines.xhtml

40 mosquitoes per experiment, simulations show we would have >90% power to detect a 70% reduction in infectivity(35).

Outside study area survey

To give a broad indication of the potential influence the presence of the study team may have on the operational delivery of SMC in arm 1, we will perform a single cross-sectional survey outside the study area. The survey is not part of any main comparisons, and an exact sample size justification is complicated. Estimates of adherence are based on questionnaire data and supported by measurements of drug levels in plasma. In arm 2 and 3 (DOT for the full course of SMC), the median level of DEAQ at day 4 following the last dose of AQ is expected to be 550 ng/mL (IQR 350-850)(15). Based on a large assessment of reported adherence and measured AQ/DEAQ plasma levels following SMC in Niger, we anticipate <50% of children show complete adherence in arm 1 and the control village and >20% of the children in these populations may fail to take any dose(9). If we conservatively assume that this results in a reduction of median DEAQ levels to <400ng/mL (IQR 200-700), we can estimate the power to detect this difference when enrolling 120 children in the SMC-targeted age range in the control area. For this, we assume that these DEAQ levels stem from right-skewed (log-normal) distributions; giving median log DEAQ concentrations of 6.31 (IQR 5.86-6.75) for DOT and 5.99 (IQR 5.30-6.55) for non-DOT. If we further assume that the distribution of the logged values is normally distributed, we can estimate the standard deviation through the IQR as IQR/1.349, giving standard deviations of 0.93 and 0.66, respectively. Using a sample size of 120 per group, and a Welch's two sample t-test for testing the differences between the log DEAQ concentrations, we estimate a power of 86.3% to detect these differences. Based on these assumptions, we are confident that sampling 120 children <5 years of age will allow meaningful assessments. As indicated above, this sample size estimation is not relevant for any of the main comparisons in the study but merely used to ensure informative sampling and avoid exposing an unnecessarily large population to the small discomfort of a single finger prick sample.

h. Study procedures

Seasonal malaria chemoprevention

SMC drug administration uses SPAQ at the conventional dosing (see **Table S1**). This medication is provided by the MoH. SMC is administered by MoH teams in arm 1 (no DOT) and by dedicated research staff in arms 2 and 3 (DOT). Clinical malaria cases will receive conventional first-line treatment with artemether-lumefantrine (AL) according to national treatment guidelines when they attend study clinics.

Scheduled surveys

Scheduled surveys will take place in each study arm and are designed and timed for several outcome measures (**Figure 3**):

For peer review only - http://bmjopen.bmj.com/site/about/guidelines.xhtml

1. A survey prior to SMC roll-out (baseline survey) is used to determine parasite carriage and to collect parasite material for genotyping infections and comparing parasite genotypes with parasites detected following SMC.

2. A mid-study survey to assess parasite carriage at different time-points following SMC and relate this to time since SMC and SMC plasma levels. These surveys are scheduled 3, 4 or 5 weeks after the last round of SMC. Each compound will participate in one of these surveys; each cluster has 3 compounds and one of these compounds is randomly selected to participate in the first, second or third mid-study survey. These surveys will include all compound members (all age groups) to also allow comparisons of gametocyte carriage and transmission potential between arms.

3. The end of study survey (endline survey) will target all age groups in the study households to determine whether parasite carriage and gametocyte carriage are reduced both in the SMC targeted populations and in the wider community. This survey will also provide information on the primary endpoint (parasite carriage by qPCR at the end of the transmission season). During these surveys, all individuals will have their axillary temperature measured and a finger prick blood sample taken onto a dried blood spot and into an EDTA-coated microtainer for collection of plasma and nucleic acids for later RNA- and DNA-work. In case of measured fever, the same finger prick blood sample will also be used for malaria diagnosis by conventional rapid diagnostic test to guide treatment according to national guidelines.

4. In addition to these surveys, there will be assessments of drug metabolites (shortly after the last round of SMC). Screening for parasite carriage by microscopy in children aged 5-9 years is scheduled on the first day of SMC rounds 2 to 4. Microscopy-positive gametocyte carriers will be prioritized for mosquito feeding assays (DMFA) to assess infectivity. Other microscopy-positive non-gametocyte carriers may be included for DMFA if mosquito numbers allow. DMFA will occur within 21 days after the first dose of SMC.

Passive case detection

In addition to these scheduled surveys and throughout the study period there is malaria passive case detection in all four health facilities that are accessed by the selected study population. Participants under 10 years will receive ID cards to facilitate identification. Trained clinical personnel are available 24/7. In case of clinical malaria, defined as microscopy-confirmed malaria infection with measured or reported fever, treatment is provided with AL, according to MoH policy. Before treatment, a finger-prick blood sample is taken for parasitological and drug metabolite assessment. Clinical incidence will be expressed per person-month in the target population (<5 years old in arm 1 and 2 and <10 years old in arms 2 and 3).

Adherence to and perceptions about SMC strategy

Selected community members and health workers will be invited for participation in the qualitative part of the study. The qualitative component of this study is designed to understand potential factors that influence SMC uptake and effectiveness, including:

1) SMC coverage – defined as proportion of eligible children reached by the four rounds of SMC;

2) Health workers compliance to MoH guidelines (proportion of children receiving a directly observed treatment for the first dose of SMC drugs as recommended by the treatment guidelines);

For peer review only - http://bmjopen.bmj.com/site/about/guidelines.xhtml

3) Caregivers' compliance – defined as the proportion of children receiving the correct dosing regimen (number of tablets and duration);

4) perceptions of the SMC program from caregiver and health system perspectives.

Perceptions will include the perceived benefits and ease of participation of SMC, as well as perceived utility of additional interventions (e.g. long lasting nets, indoor residual spraying, personal protective measures) when adhering to SMC.

For participation in the qualitative study on SMC adherence and perceptions, there are distinct inclusion and exclusion criteria for community members and health workers (**Table 2**).

Throughout the study, data on compliance to SMC treatment guidelines from caregiver and healthcare worker perspectives are collected in arm 1. Whether first dose was directly observed and whether the full treatment course was administered to the child after each round on SMC is documented. After SMC round 4 a cross-sectional survey will be carried out. For this, individual interviews of caregivers of children targeted or not by the current SMC strategy will be conducted. Further, within each of the study arms, between 6-8 individuals will be recruited for focus group discussions.

Key informant interviews (KIIs) and in-depth interviews (IDIs) will be carried out. For the KIIs, participants will be recruited through purposive sampling to include stakeholders within the health system involved in SMC implementation. The IDIs will be conducted among 5-10 key informants per each of the study arms. These individuals will also be purposively selected aiming at including community leaders, professionals, or residents who have first-hand knowledge about the community.

i. Laboratory assessments

<u>Parasitology</u>

Thick and/or thin blood films for parasite counts will be obtained at the timepoint of the first SMC dose for rounds 2-4, during clinical malaria episodes and during cross-sectional sampling to select study participants for assessment of infectivity by DMFA.

Further, parasite DNA and RNA will be extracted from whole blood samples in EDTA tubes and tested using qPCR that targets varATS and detects all *P. falciparum* parasites (asexual parasites and gametocytes). Parasite prevalence and density will be assessed as intervention outcome measures, as previously done for community interventions(30).

P. falciparum gametocytes will be quantified by quantitative reverse transcriptase PCR targeting female (Ccp4) and male gametocytes (PfMGET)(36, 37). For a subset of infections, multiplicity of infection and the genetic relatedness of infections that are detected in the same study participant over time will be determined (e.g. AMA-1 amplicon deep sequencing). This amplicon sequencing will be performed in a selection of DNA samples that are parasite positive by varATS qPCR and for whom re-infection or infection persistence is suspected.

Assessments of molecular markers of resistance will follow established protocols(13). Nucleic acids will be extracted with commercial kits [Qiagen] and stored at –20°C until use. Three dual-labelled probes were designed to detect three crt genotypes at codons 72–76 (encoding Cys-Val-Met-Asn-Lys

For peer review only - http://bmjopen.bmj.com/site/about/guidelines.xhtml

[CVMNK], CVIET, and SVMNT). Laboratory isolates *P. falciparum* 3D7, Dd2, and 7G8 will be used as positive controls for the CVMNK, CVIET, and SVMNT haplotypes, respectively. qPCR amplification will be done in a single well to maximise throughput using a thermocycler (BioRad). All samples will further be tested for mdr1, dhfr, and dhps. For this, all three genes will be amplified by nested PCR using previously described methods (Pearce et al, 2003) and polymorphisms identified by direct sequencing of amplified products (BigDye Terminator v3.1 cycle sequencing kits and ABI 3730 sequencer; ThermoFisher Scientific) and data will be analysed using Geneious v10.1.3 (Biomatters, San Diego, CA, USA).

Pharmacology

AQ and DEAQ concentrations will be quantified from plasma samples and filter paper spots. Blood concentrations are measured within 6 months of sample collection using a liquid chromatography tandem mass spectrometry assay(9, 38).

Serology

Serological analysis of these samples will be conducted using bead-based assays to assess:

- presence of biomarkers for correlates of infection and infectivity (i.e. proteins associated with inflammation), or parasite density (HRP2)(39).
- antibody responses to gametocyte and asexual stage proteins in established multiplex platforms(40). Antigens will include but not be restricted too Pfs230, Pfs48/45 for sexual stage proteins to assess the influence on transmission to mosquitoes and PfAMA, PfMSP-1 and Pf ETRAMP 5 to assess the effect of interventions on the development of humoral immunity.

Mosquito feeding assays

For each assessment of infectivity, 2 mL of heparinized venous blood will be drawn from the study participant selected for DMFA. At the insectary, ~70 *Anopheles gambiae s.s.* mosquitoes will be fed on the subjects' blood for 15-20 minutes using a water-jacketed feeder system(30). Mosquitoes will be dissected in 0.5% mercurochrome on the 7th day after the feeding assay for the prevalence of mosquitoes with oocysts and quantification of oocysts.

j. Randomization

The unit of randomization is the cluster; each cluster comprises of 3 compounds. To account for spatial differences in malaria exposure, distance from health facilities, and treatment seeking behaviour, neighbouring clusters are grouped before randomization. Clusters are randomly allocated to one of the three study arms by a computer-generated algorithm and stratified by village. In this way clusters are spatially 'matched' and are thus likely to experience similar malaria exposure. In total, the randomization resulted in 185 compounds (61 to 62 clusters with 3 compounds each) in each arm. Endpoints are evaluated at the level of the individual study participant.

k. Analyses plan

Primary endpoint:

For peer review only - http://bmjopen.bmj.com/site/about/guidelines.xhtml

The primary endpoint on parasite prevalence (by qPCR) at the end of the transmission season in age groups targeted by SMC will be compared in children aged 3-59 months in study arms 1 and 2 and in children aged 5 to 9 years in study arms 2 and 3. Parasite prevalence by qPCR will be used as binary variable with all parasite densities above 100 parasites/mL (0.1 parasite/µL) being classified as parasite positive. Parasite prevalence is compared between arms by logistic regression; models will account for clustering by including compound ID as a random intercept and village as a fixed effect.

Secondary and exploratory endpoints:

A complete statistical analysis plan (SAP) has been developed for this trial. All parasite prevalence related endpoints will be compared as described above for the primary endpoint. Gametocyte prevalence related endpoints will be presented as prevalence estimate with 95% confidence interval.

Ethics and dissemination

The study has been reviewed and approved by the London School of Hygiene and Tropical Medicine (LSHTM) (Review number: 29193; Version 2.0, July 2023) and Burkina Faso National Medical Ethics Committees (Deliberation N° 2023-05-104). Data will be stored on secure servers at GRAS and shared with primary project partners as de-identified. A study participant identification link log is available at the investigators' site. Data will be deposited with publications were required and uploaded to shared data repositories for broader use by the scientific community (e.g ClinEpi DB).

The findings of the study will be shared with the community via the local opinion leaders. The project is followed by a community meeting to present some of the preliminary findings. Disease occurrence data will be shared with the Ministry of Health. The results will also be shared in peer-reviewed publications.

Trial status

This study is registered on ClinicalTrials.gov #NCT05878366.

ACKNOWLEDGMENTS

The authors thank the Ministry of Health and National Malaria Control Program from Burkina Faso and staff at the study sites for support, as well as the research nurses, laboratory and administrative staff involved in the study.

Footnotes

MM, AB and MG are joint first authors. CD and AT are joint senior authors.

AUTHOR CONTRIBUTIONS

ABT, TB, and CD developed and designed the study. MM, AB, MG, SSS, AO and JB contributed to study design. All authors contributed to protocol development. MM, MG, TB, CD and ABT wrote the first draft of the manuscript. All authors reviewed and approved the final draft of the manuscript.

FUNDING STATEMENT

For peer review only - http://bmjopen.bmj.com/site/about/guidelines.xhtml

For peer review only

This work is supported by Bill and Melinda Gates Foundation grant (INV-053846). The funders, Bill and Melinda Gates Foundation, were involved in study design, but had no role in data collection, analysis, interpretation or reporting.

COMPETING INTERESTS

None declared.

REFERENCES

1. Organization WH. World malaria report 2022. 2022. Contract No.: CC BY-NC-SA 3.0 IGO.
2. Das S, Saha B, Hati AK, Roy S. Evidence of Artemisinin-Resistant Plasmodium falciparum Malaria in Eastern India. N Engl J Med. 2018;379(20):1962-4.
3. Partnership A-S. Effectiveness of seasonal malaria chemoprevention at scale in west and central Africa: an observational study. Lancet. 2020;396(10265):1829-40.
4. Ashley EA, Poespoprodjo JR. Treatment and prevention of malaria in children. Lancet Child Adolesc Health. 2020;4(10):775-89.
5. Cairns M, Ceesay SJ, Sagara I, Zongo I, Kessely H, Gamougam K, et al. Effectiveness of seasonal malaria chemoprevention (SMC) treatments when SMC is implemented at scale: Case-control studies in 5 countries. PLoS Med. 2021;18(9):e1003727.
6. Organization WH. Updated WHO recommendations for malaria chemoprevention among children and pregnant women. Geneva, Switzerland; 2022.
7. Cissé B, Ba EH, Sokhna C, JL ND, Gomis JF, Dial Y, et al. Effectiveness of Seasonal Malaria Chemoprevention in Children under Ten Years of Age in Senegal: A Stepped-Wedge Cluster-Randomised Trial. PLoS Med. 2016;13(11):e1002175.
8. malERA. malERA: An updated research agenda for health systems and policy research in malaria elimination and eradication. PLoS Med. 2017;14(11):e1002454.
9. Ding J, Coldiron ME, Assao B, Guindo O, Blessborn D, Winterberg M, et al. Adherence and Population Pharmacokinetic Properties of Amodiaquine When Used for Seasonal Malaria Chemoprevention in African Children. Clin Pharmacol Ther. 2020;107(5):1179-88.
10. Chandramohan D, Zongo I, Sagara I, Cairns M, Yerbanga RS, Diarra M, et al. Seasonal Malaria Vaccination with or without Seasonal Malaria Chemoprevention. N Engl J Med. 2021;385(11):1005-17.
11. S. Soremekun BC, A. Nyassi, H. Soumare, B. Etoketim, M. Ndiath, J. Bradley, U. D'Alessandro, T. Bousema, A. Erhart, M. Moreno, C. Drakeley. Household-level effects of seasonal malaria chemoprevention in the Gambia. Research Square. 2023.
12. Naidoo I, Roper C. Mapping 'partially resistant', 'fully resistant', and 'super resistant' malaria. Trends Parasitol. 2013;29(10):505-15.
13. Beshir KB, Muwanguzi J, Nader J, Mansukhani R, Traore A, Gamougam K, et al. Prevalence of Plasmodium falciparum haplotypes associated with resistance to sulfadoxine-pyrimethamine and amodiaquine before and after upscaling of seasonal malaria chemoprevention in seven African countries: a genomic surveillance study. Lancet Infect Dis. 2023;23(3):361-70.
14. Nuwa A, Baker K, Bonnington C, Odongo M, Kyagulanyi T, Bwanika JB, et al. A non-randomized controlled trial to assess the protective effect of SMC in the context of high parasite resistance in Uganda. Malar J. 2023;22(1):63.
15. Anyorigiya TA, Castel S, Mauff K, Atuguba F, Ogutu B, Oduro A, et al. Pharmacokinetic profile of amodiaquine and its active metabolite desethylamodiaquine in Ghanaian patients with uncomplicated falciparum malaria. Malar J. 2021;20(1):18.

For peer review only - http://bmjopen.bmj.com/site/about/guidelines.xhtml

16. Humphreys GS, Merinopoulos I, Ahmed J, Whitty CJ, Mutabingwa TK, Sutherland CJ, et al. Amodiaquine and artemether-lumefantrine select distinct alleles of the Plasmodium falciparum mdr1 gene in Tanzanian children treated for uncomplicated malaria. Antimicrob Agents Chemother. 2007;51(3):991-7.
17. Bousema JT, Schneider P, Gouagna LC, Drakeley CJ, Tostmann A, Houben R, et al. Moderate Effect of Artemisinin-Based Combination Therapy on Transmission of Plasmodium falciparum. Journal of Infectious Diseases. 2006;193(8):1151-9.
18. Alkema M, Reuling IJ, de Jong GM, Lanke K, Coffeng LE, van Gemert GJ, et al. A Randomized Clinical Trial to Compare Plasmodium falciparum Gametocytemia and Infectivity After Blood-Stage or Mosquito Bite-Induced Controlled Malaria Infection. J Infect Dis. 2021;224(7):1257-65.
19. Kone A, van de Vegte-Bolmer M, Siebelink-Stoter R, van Gemert GJ, Dara A, Niangaly H, et al. Sulfadoxine-pyrimethamine impairs Plasmodium falciparum gametocyte infectivity and Anopheles mosquito survival. Int J Parasitol. 2010;40(10):1221-8.
20. Dicko A, Roh ME, Diawara H, Mahamar A, Soumare HM, Lanke K, et al. Efficacy and safety of primaquine and methylene blue for prevention of Plasmodium falciparum transmission in Mali: a phase 2, single-blind, randomised controlled trial. The Lancet Infectious Diseases. 2018;18(6):627-39.
21. Andolina C, Rek JC, Briggs J, Okoth J, Musiime A, Ramjith J, et al. Sources of persistent malaria transmission in a setting with effective malaria control in eastern Uganda: a longitudinal, observational cohort study. Lancet Infect Dis. 2021;21(11):1568-78.
22. Goncalves BP, Kapulu MC, Sawa P, Guelbeogo WM, Tiono AB, Grignard L, et al. Examining the human infectious reservoir for Plasmodium falciparum malaria in areas of differing transmission intensity. Nat Commun. 2017;8(1):1133.
23. Rek J, Blanken SL, Okoth J, Ayo D, Onyige I, Musasizi E, et al. Asymptomatic school-aged children are important drivers of malaria transmission in a high endemicity setting in Uganda. J Infect Dis. 2022.
24. Staedke SG, Maiteki-Sebuguzi C, Rehman AM, Kigozi SP, Gonahasa S, Okiring J, et al. Assessment of community-level effects of intermittent preventive treatment for malaria in schoolchildren in Jinja, Uganda (START-IPT trial): a cluster-randomised trial. Lancet Glob Health. 2018;6(6):e668-e79.
25. Guelbéogo WM, Gonçalves BP, Grignard L, Bradley J, Serme SS, Hellewell J, et al. Variation in natural exposure to anopheles mosquitoes and its effects on malaria transmission. Elife. 2018;7.
26. Cohee LM, Opondo C, Clarke SE, Halliday KE, Cano J, Shipper AG, et al. Preventive malaria treatment among school-aged children in sub-Saharan Africa: a systematic review and meta-analyses. Lancet Glob Health. 2020;8(12):e1499-e511.
27. Chen I, Clarke SE, Gosling R, Hamainza B, Killeen G, Magill A, et al. "Asymptomatic" Malaria: A Chronic and Debilitating Infection That Should Be Treated. PLoS Med. 2016;13(1):e1001942.
28. World Health Organisation. Updated WHO recommendations for malaria chemoprevention among children and pregnant women. 2022.
29. Chan AW, Tetzlaff JM, Altman DG, Laupacis A, Gotzsche PC, Krleza-Jeric K, et al. SPIRIT 2013 statement: defining standard protocol items for clinical trials. Ann Intern Med. 2013;158(3):200-7.
30. Collins KA, Ouedraogo A, Guelbeogo WM, Awandu SS, Stone W, Soulama I, et al. Investigating the impact of enhanced community case management and monthly screening and treatment on the transmissibility of malaria infections in Burkina Faso: study protocol for a cluster-randomised trial. BMJ Open. 2019;9(9):e030598.
31. Barry A, Bradley J, Stone W, Guelbeogo MW, Lanke K, Ouedraogo A, et al. Higher gametocyte production and mosquito infectivity in chronic compared to incident Plasmodium falciparum infections. Nat Commun. 2021;12(1):2443.
32. Bradley J, Stone W, Da DF, Morlais I, Dicko A, Cohuet A, et al. Predicting the likelihood and intensity of mosquito infection from sex specific Plasmodium falciparum gametocyte density. Elife. 2018;7.

For peer review only - http://bmjopen.bmj.com/site/about/guidelines.xhtml

33. Bradley J, Soumaré HM, Mahamar A, Diawara H, Roh M, Delves M, et al. Transmission-blocking Effects of Primaquine and Methylene Blue Suggest Plasmodium falciparum Gametocyte Sterilization Rather Than Effects on Sex Ratio. Clin Infect Dis. 2019;69(8):1436-9.
34. Stone W, Mahamar A, Smit MJ, Sanogo K, Sinaba Y, Niambele SM, et al. Single low-dose tafenoquine combined with dihydroartemisinin-piperaquine to reduce Plasmodium falciparum transmission in Ouelessebougou, Mali: a phase 2, single-blind, randomised clinical trial. Lancet Microbe. 2022;3(5):e336-e47.
35. Ramjith J, Alkema M, Bradley J, Dicko A, Drakeley C, Stone W, et al. Quantifying Reductions in Plasmodium falciparum Infectivity to Mosquitos: A Sample Size Calculator to Inform Clinical Trials on Transmission-Reducing Interventions. Front Immunol. 2022;13:899615.
36. Schneider P, Schoone G, Schallig H, Verhage D, Telgt D, Eling W, et al. Quantification of Plasmodium falciparum gametocytes in differential stages of development by quantitative nucleic acid sequence-based amplification. MolBiochemParasitol. 2004;137(1):35-41.
37. Meerstein-Kessel L, Andolina C, Carrio E, Mahamar A, Sawa P, Diawara H, et al. A multiplex assay for the sensitive detection and quantification of male and female Plasmodium falciparum gametocytes. Malaria Journal. 2018;17(1):441.
38. Roh ME, Zongo I, Haro A, Huang L, Some AF, Yerbanga RS, et al. Seasonal Malaria Chemoprevention Drug Levels and Drug Resistance Markers in Children With or Without Malaria in Burkina Faso: A Case-Control Study. J Infect Dis. 2023;228(7):926-35.
39. Oulton T, Mahamar A, Sanogo K, Diallo M, Youssouf A, Niambele SM, et al. Persistence of Plasmodium falciparum HRP-2 antigenaemia after artemisinin combination therapy is not associated with gametocytes. Malar J. 2022;21(1):372.
40. de Jong RM, Alkema M, Oulton T, Dumont E, Teelen K, Nakajima R, et al. The acquisition of humoral immune responses targeting Plasmodium falciparum sexual stages in controlled human malaria infections. Front Immunol. 2022;13:930956.

For peer review only

For peer review only - http://bmjopen.bmj.com/site/about/guidelines.xhtml

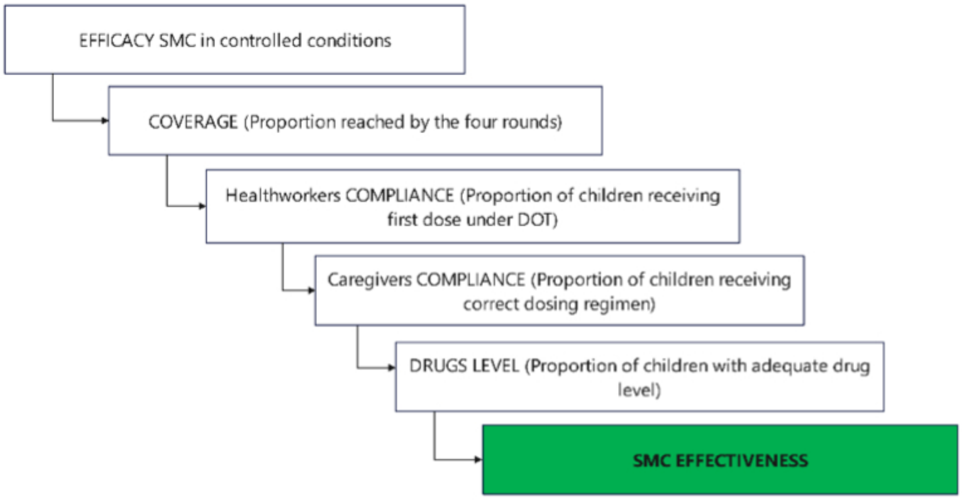

Figure 1. Cascade in efficacy decay in SMC effectiveness. Coverage, compliance, and drug absorption will all influence the actual efficiency that is observed when SMC is implemented under programmatic conditions(28).DOT: Directly observed treatment.

342x185mm (59 x 59 DPI)

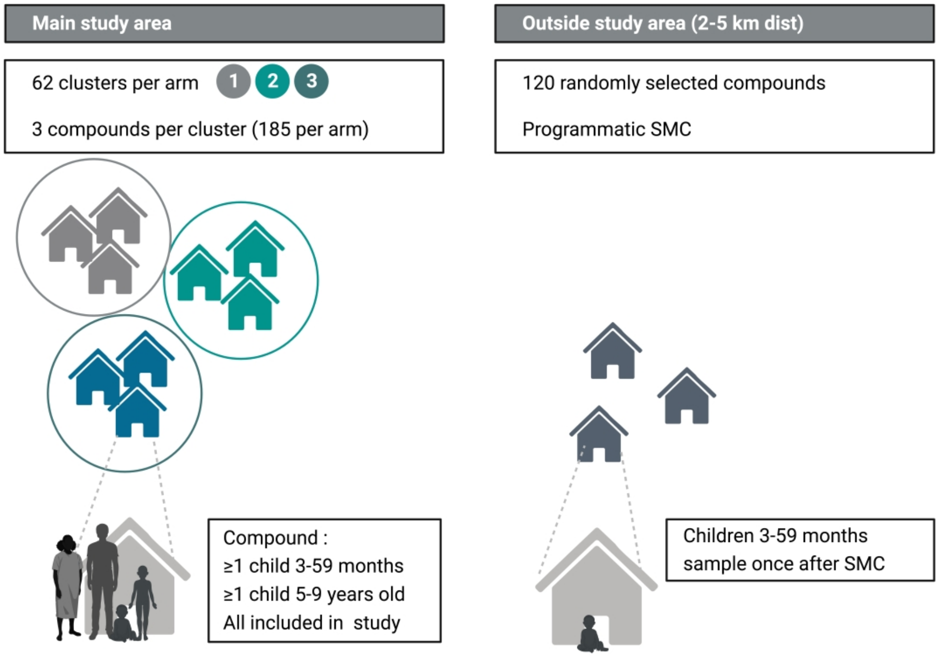

Figure 2. Study design. The study will be implemented in clusters. Each cluster consists of 3 compounds where each compound has at least one child aged 3-59 months and one child aged 5-9 years. All age groups will take part in the evaluation of the study to assess the impact of the intervention on the infectious reservoir in the community and compare parasite carriage in targeted and untargeted populations. Figure was created with BioRender.com.

645x452mm (236 x 236 DPI)

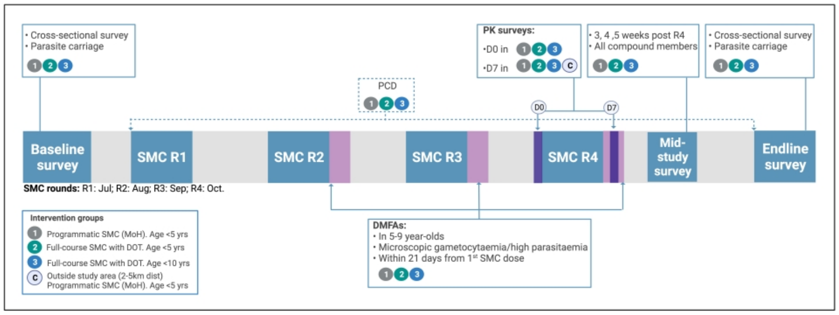

Figure 3. Surveys and sampling schedule. Cluster randomization was performed prior to the baseline study. PCD: Passive case detection; PK surveys: Pharmacokinetics analysis; DMFAs: direct membrane feeding assays, done up to 21 days from 1st SMC dose. Adherence to and perceptions about SMC strategy will be collected throughout the study in the three study arms. Figure was created with BioRender.com.

645x452mm (236 x 236 DPI)

Supplementary data

Title: Understanding and maximizing the community impact of seasonal malaria chemoprevention in Burkina Faso (INDIE-SMC): study protocol for a cluster randomized evaluation trial

Authors: Moreno M[1*], Barry A[2,3*], Gmeiner M[4*], Yaro JB[2], Sermé SS[2], Byrne I[1], Ramjith J[4], Ouedraogo A[2], Soulama I[2], Grignard L[1], Soremekun S[1], Koele S[4], ter Heine R[4], Ouedraogo AZ[2], Sawadogo J[2], Sanogo E[2], Ouedraogo IN[2], Hien D[2], Sirima SB[2], Bradley J[1], Bousema T[4], Drakeley C[1*], Tiono AB[2*§]

1 Department of Infection Biology, London School of Hygiene and Tropical Medicine, London, UK

2 Groupe de Recherche Action Santé, Ouagadougou, Burkina Faso

3 Secretariat Permanent pour l'élimination du Paludisme, Ministère de la Santé et de l'hygiène publique, Ouagadougou, Burkina Faso

4 Department of Medical Microbiology, Radboud University Medical Centre, Nijmegen, The Netherlands

Table S1. SPAQ dosing according to Burkina Faso MoH. *NA: not applicable.*

Age	SMC Drugs	Day 1	Day 2	Day 3
**3-11 months**	SP : 262.5 mg S, fixed dose fixe with P	1 Tablet	*NA*	*NA*
	AQ : 76.5 mg	1 Tablet	1 Tablet	1 Tablet
**12-59 months**	SP : 525 mg S	1 Tablet	*NA*	*NA*
	AQ : 153 mg	1 Tablet	1 Tablet	1 Tablet
**5-9 years**	SP : 525 mg S	1 Tablet	*NA*	*NA*
	SP : 262.5 mg S	1 Tablet	*NA*	*NA*
	AQ : 153 mg	1 Tablet	1 Tablet	1 Tablet
	AQ : 76.5 mg	1 Tablet	1 Tablet	1 Tablet

For peer review only

Reporting checklist for protocol of a clinical trial.

Based on the SPIRIT guidelines.

		Reporting Item	Page Number
**Administrative information**			
Title	#1	Descriptive title identifying the study design, population, interventions, and, if applicable, trial acronym	1
Trial registration	#2a	Trial identifier and registry name. If not yet registered, name of intended registry	2, 16
Trial registration: data set	#2b	All items from the World Health Organization Trial Registration Data Set	16
Protocol version	#3	Date and version identifier	16
Funding	#4	Sources and types of financial, material, and other support	16
Roles and responsibilities: contributorship	#5a	Names, affiliations, and roles of protocol contributors	16
Roles and responsibilities: sponsor contact information	#5b	Name and contact information for the trial sponsor	16
Roles and responsibilities: sponsor and funder	#5c	Role of study sponsor and funders, if any, in study design; collection, management, analysis, and interpretation of data; writing of the report; and the decision to submit the report for publication, including whether they will have ultimate authority over any of these activities	16

For peer review only

Roles and responsibilities: committees	#5d	Composition, roles, and responsibilities of the coordinating centre, steering committee, endpoint adjudication committee, data management team, and other individuals or groups overseeing the trial, if applicable (see Item 21a for data monitoring committee)	*16. An independent DAC review team (Design, Analyze, Communicate) program from Bill & Melinda Gates Foundation (reference #0000000314) oversees the trial.*
**Introduction**			
Background and rationale	#6a	Description of research question and justification for undertaking the trial, including summary of relevant studies (published and unpublished) examining benefits and harms for each intervention	3-5
Background and rationale: choice of comparators	#6b	Explanation for choice of comparators	5
Objectives	#7	Specific objectives or hypotheses	5-6
Trial design	#8	Description of trial design including type of trial (eg, parallel group, crossover, factorial, single group), allocation ratio, and framework (eg, superiority, equivalence, non-inferiority, exploratory)	6
**Methods: Participants, interventions, and outcomes**			
Study setting	#9	Description of study settings (eg, community clinic, academic hospital) and list of countries where data will be collected. Reference to where list of study sites can be obtained	8
Eligibility criteria	#10	Inclusion and exclusion criteria for participants. If applicable, eligibility criteria for study centres and individuals who will	9-10

For peer review only

		perform the interventions (eg, surgeons, psychotherapists)	
Interventions: description	#11a	Interventions for each group with sufficient detail to allow replication, including how and when they will be administered	6
Interventions: modifications	#11b	Criteria for discontinuing or modifying allocated interventions for a given trial participant (eg, drug dose change in response to harms, participant request, or improving / worsening disease)	*NA. This is an evaluation of a programmatic activity of the MoH.*
Interventions: adherance	#11c	Strategies to improve adherence to intervention protocols, and any procedures for monitoring adherence (eg, drug tablet return; laboratory tests)	*13. The MoH records performs surveillance of these parameters.*
Interventions: concomitant care	#11d	Relevant concomitant care and interventions that are permitted or prohibited during the trial	13
Outcomes	#12	Primary, secondary, and other outcomes, including the specific measurement variable (eg, systolic blood pressure), analysis metric (eg, change from baseline, final value, time to event), method of aggregation (eg, median, proportion), and time point for each outcome. Explanation of the clinical relevance of chosen efficacy and harm outcomes is strongly recommended	8
Participant timeline	#13	Time schedule of enrolment, interventions (including any run-ins and washouts), assessments, and visits for participants. A schematic diagram is highly recommended (see Figure)	12, Figure 3
Sample size	#14	Estimated number of participants needed to achieve study objectives and how it was determined, including clinical and	10-11

For peer review only

		statistical assumptions supporting any sample size calculations	
Recruitment	#15	Strategies for achieving adequate participant enrolment to reach target sample size	10
**Methods: Assignment of interventions (for controlled trials)**			
Allocation: sequence generation	#16a	Method of generating the allocation sequence (eg, computer-generated random numbers), and list of any factors for stratification. To reduce predictability of a random sequence, details of any planned restriction (eg, blocking) should be provided in a separate document that is unavailable to those who enrol participants or assign interventions	15
Allocation concealment mechanism	#16b	Mechanism of implementing the allocation sequence (eg, central telephone; sequentially numbered, opaque, sealed envelopes), describing any steps to conceal the sequence until interventions are assigned	15
Allocation: implementation	#16c	Who will generate the allocation sequence, who will enrol participants, and who will assign participants to interventions	15
Blinding (masking)	#17a	Who will be blinded after assignment to interventions (eg, trial participants, care providers, outcome assessors, data analysts), and how	*NA. The nature of the study (programmatic activity from the MoH) prevents from blinding participation.*
Blinding (masking): emergency unblinding	#17b	If blinded, circumstances under which unblinding is permissible, and procedure for revealing a participant's allocated intervention during the trial	NA

For peer review only

**Methods: Data collection, management, and analysis**			
Data collection plan	#18a	Plans for assessment and collection of outcome, baseline, and other trial data, including any related processes to promote data quality (eg, duplicate measurements, training of assessors) and a description of study instruments (eg, questionnaires, laboratory tests) along with their reliability and validity, if known. Reference to where data collection forms can be found, if not in the protocol	12-15
Data collection plan: retention	#18b	Plans to promote participant retention and complete follow-up, including list of any outcome data to be collected for participants who discontinue or deviate from intervention protocols	13
Data management	#19	Plans for data entry, coding, security, and storage, including any related processes to promote data quality (eg, double data entry; range checks for data values). Reference to where details of data management procedures can be found, if not in the protocol	15
Statistics: outcomes	#20a	Statistical methods for analysing primary and secondary outcomes. Reference to where other details of the statistical analysis plan can be found, if not in the protocol	15
Statistics: additional analyses	#20b	Methods for any additional analyses (eg, subgroup and adjusted analyses)	*The team has developed a complete statistical analysis plan (SAP) not included in the published protocol, but it could be shared upon request.*

For peer review only

Statistics: analysis population and missing data	#20c	Definition of analysis population relating to protocol non-adherence (eg, as randomised analysis), and any statistical methods to handle missing data (eg, multiple imputation)	11, 13. *More details in SAP.*
**Methods: Monitoring**			
Data monitoring: formal committee	#21a	Composition of data monitoring committee (DMC); summary of its role and reporting structure; statement of whether it is independent from the sponsor and competing interests; and reference to where further details about its charter can be found, if not in the protocol. Alternatively, an explanation of why a DMC is not needed	*The protocol has been reviewed by the Design, Analyze, Communicate (DAC) program from Bill & Melinda Gates Foundation by an independent DAC review team (reference #0000000314).*
Data monitoring: interim analysis	#21b	Description of any interim analyses and stopping guidelines, including who will have access to these interim results and make the final decision to terminate the trial	NA*. Programmatic activity from MoH.*
Harms	#22	Plans for collecting, assessing, reporting, and managing solicited and spontaneously reported adverse events and other unintended effects of trial interventions or trial conduct	NA. *The MoH records any adverse events routinely during this programmatic intervention.*
Auditing	#23	Frequency and procedures for auditing trial conduct, if any, and whether the process will be independent from investigators and the sponsor	NA. *This is an evaluation of a programmatic intervention from the MoH.*
**Ethics and dissemination**			
Research ethics approval	#24	Plans for seeking research ethics committee / institutional review board (REC / IRB) approval	16

For peer review only

Protocol amendments	#25	Plans for communicating important protocol modifications (eg, changes to eligibility criteria, outcomes, analyses) to relevant parties (eg, investigators, REC / IRBs, trial participants, trial registries, journals, regulators)	NA. *Protocol amendments will be consensual between all parts involved and informed to the relevant Ethics committees.*
Consent or assent	#26a	Who will obtain informed consent or assent from potential trial participants or authorised surrogates, and how (see Item 32)	10
Consent or assent: ancillary studies	#26b	Additional consent provisions for collection and use of participant data and biological specimens in ancillary studies, if applicable	*There is a clause regarding this issue in the Informed Consents and approved by Ethics committees.*
Confidentiality	#27	How personal information about potential and enrolled participants will be collected, shared, and maintained in order to protect confidentiality before, during, and after the trial	*All data in the database is pseudonymized. A study participant identification link log is available at the investigators' site.*
Declaration of interests	#28	Financial and other competing interests for principal investigators for the overall trial and each study site	16
Data access	#29	Statement of who will have access to the final trial dataset, and disclosure of contractual agreements that limit such access for investigators	16
Ancillary and post trial care	#30	Provisions, if any, for ancillary and post-trial care, and for compensation to those who suffer harm from trial participation	*This is included in the Information sheet to all participants and approved by Ethics committees.*
Dissemination policy: trial results	#31a	Plans for investigators and sponsor to communicate trial results to participants, healthcare professionals, the public, and other relevant groups (eg, via publication, reporting in results databases, or other	16

For peer review only

		data sharing arrangements), including any publication restrictions	
Dissemination policy: authorship	#31b	Authorship eligibility guidelines and any intended use of professional writers	16
Dissemination policy: reproducible research	#31c	Plans, if any, for granting public access to the full protocol, participant-level dataset, and statistical code	16
**Appendices**			
Informed consent materials	#32	Model consent form and other related documentation given to participants and authorised surrogates	*Upon request to investigators*.
Biological specimens	#33	Plans for collection, laboratory evaluation, and storage of biological specimens for genetic or molecular analysis in the current trial and for future use in ancillary studies, if applicable	14-15. *Submitted and approved to Ethics committee.*

For peer review only

BMJ Open

Understanding and maximizing the community impact of seasonal malaria chemoprevention in Burkina Faso (INDIE-SMC): study protocol for a cluster randomized evaluation trial

Journal:	*BMJ Open*
Manuscript ID	bmjopen-2023-081682.R1
Article Type:	Protocol
Date Submitted by the Author:	05-Feb-2024
Complete List of Authors:	Moreno, Marta; London School of Hygiene & Tropical Medicine, Department of Infection Biology<br>Barry, Aissata; Groupe de Recherche Action en Santé<br>Gmeiner, Markus; Radboud University Nijmegen Department of Medical Microbiology<br>Yaro, Jean ; Groupe de Recherche Action en Santé<br>Sermé, Samuel ; Groupe de Recherche Action en Santé<br>Byrne, Isabel; London School of Hygiene & Tropical Medicine, Department of Infection Biology<br>Ramjith, Jordache; Radboud University Nijmegen Department of Medical Microbiology<br>Ouedraogo, Alphonse; Groupe de Recherche Action en Santé<br>Soulama, Issiaka; Groupe de Recherche Action en Santé<br>Grignard, Lynn; London School of Hygiene & Tropical Medicine, Department of Infection Biology<br>Soremekun, Seyi; London School of Hygiene & Tropical Medicine, Department of Infection Biology<br>Koele, S; Radboud University Nijmegen Department of Medical Microbiology<br>ter Heine, R; Radboudumc, Pharmacy<br>Ouedraogo, Amidou; Groupe de Recherche Action en Santé<br>Sawadogo, Jean; Groupe de Recherche Action en Santé<br>Sanogo, Edith; Groupe de Recherche Action en Santé<br>Ouedraogo, Issa; Groupe de Recherche Action en Santé<br>Hien, Denise; Groupe de Recherche Action en Santé<br>Sirima, Sodiomon Bienvenu; Groupe de Recherche Action en Santé<br>Bradley, John; London School of Hygiene & Tropical Medicine, Department of Infection Biology<br>Bousema, Teun; Radboud University Nijmegen Department of Medical Microbiology<br>Drakeley, Chris; London School of Hygiene & Tropical Medicine, Department of Infection Biology<br>Tiono, Alfred B. ; Groupe de Recherche Action en Santé
**Primary Subject Heading**:	Infectious diseases
Secondary Subject Heading:	Epidemiology

Keywords:	Malaria, EPIDEMIOLOGIC STUDIES, Tropical medicine < INFECTIOUS DISEASES

SCHOLARONE™ Manuscripts

BMJ Open

Title: Understanding and maximizing the community impact of seasonal malaria chemoprevention in Burkina Faso (INDIE-SMC): study protocol for a cluster randomized evaluation trial

Authors: Moreno M[1*], Barry A[2,3*], Gmeiner M[4*], Yaro JB[2], Sermé SS[2], Byrne I[1], Ramjith J[4], Ouedraogo A[2], Soulama I[2], Grignard L[1], Soremekun S[1], Koele S[4], ter Heine R[4], Ouedraogo AZ[2], Sawadogo J[2], Sanogo E[2], Ouedraogo IN[2], Hien D[2], Sirima SB[2], Bradley J[1], Bousema T[4], Drakeley C[1*], Tiono AB[2*§]

1 Department of Infection Biology, London School of Hygiene and Tropical Medicine, London, UK

2 Groupe de Recherche Action Santé, Ouagadougou, Burkina Faso

3 Secretariat Permanent pour l'élimination du Paludisme, Ministère de la santé et de l'hygiène publique, Ouagadougou, Burkina Faso

4 Department of Medical Microbiology, Radboud University Medical Center, Nijmegen, The Netherlands

* Contributed equally

[§]Corresponding author:

Alfred B Tiono

Email: a.tiono@gras.bf

Address: Groupe de Recherche Action en Santé (GRAS)

06 BP 10248 Ouagadougou 06, Burkina Faso

Tél: +226 25 35 56 90/73

Email addresses:

MM: Marta.moreno@lshtm.ac.uk

AB: a.barry@gras.bf

MG: markus.gmeiner@radboudumc.nl

JBY: j.yaro@gras.bf

SSS: s.serme@gras.bf

IB: isabel.byrne@lshtm.ac.uk

JR: jordache.ramjith@radboudumc.nl

AO: a.ouedraogo@gras.bf

IS: i.soulama@gras.bf

LG: lynn.grignard@lshtm.ac.uk

For peer review only

SS: seyi.soremekun@lshtm.ac.uk

SK: simon.koele@radboudumc.nl

RH: r.terheine@radboudumc.nl

AZO: oz.amidou@gras.bf

JS: j.sawadogo@gras.bf

ES: e.sanogo@gras.bf

INO: i.ouedraogo@gras.bf

DH: d.hien@gras.bf

SBS: s.sirima@gras.bf

JB: John.Bradley@lshtm.ac.uk

TB: Teun.Bousema@radboudumc.nl

CD: Chris.drakeley@lshtm.ac.uk

ABT: a.tiono@gras.bf

For peer review only

For peer review only - http://bmjopen.bmj.com/site/about/guidelines.xhtml

Abstract

Introduction Seasonal Malaria Chemoprevention (SMC) involves repeated administrations of sulfadoxine-pyrimethamine plus amodiaquine to children below the age of 5 years during the peak transmission season in areas of seasonal malaria transmission. Whilst highly impactful in reducing *Plasmodium falciparum* malaria burden in controlled research settings, the impact of SMC on infection prevalence is moderate in real-life settings. It remains unclear what drives this efficacy decay. Recently, the World Health Organization widened the scope for SMC to target all vulnerable populations. The Ministry of Health (MoH) in Burkina Faso is considering extending SMC to children below 10 years old. We aim to assess the impact of SMC on clinical incidence and parasite prevalence and quantify the human infectious reservoir for malaria in this population.

Methods and analysis We will perform a cluster-randomized trial in Saponé Health District, Burkina Faso, with three study arms comprising 62 clusters of 3 compounds: arm 1 (control): SMC in under 5-year-old children, implemented by the MoH without directly observed treatment (DOT) for the full course of SMC; arm 2 (intervention): SMC in under 5-year –old children, with DOT for the full course of SMC; arm 3 (intervention): SMC in under 10-year-old children, with DOT for the full course of SMC. The primary endpoint is parasite prevalence at the end of the malaria transmission season. Secondary endpoints include the impact of SMC on clinical incidence. Factors affecting SMC uptake, treatment adherence, drug concentrations, parasite resistance markers and transmission of parasites will be determined.

Ethics and dissemination The London School of Hygiene and Tropical Medicine's Ethics Committee (#29193) and the Burkina Faso National Medical Ethics Committee (Deliberation N° 2023-05-104) approved this study. The findings will be presented to the community; disease occurrence data and study outcomes will also be shared with the Burkina Faso MoH. Findings will be published irrespective of their results.

Trial registration number

NCT05878366

Keywords: malaria, seasonal malaria chemoprevention, sulfadoxine-pyrimethamine, amodiaquine, *Plasmodium falciparum*

Strengths and limitations of this study

- Strong engagement with National Malaria Control Program (NMCP) in Burkina Faso to tailor data collection to local needs.
- This study quantifies for the first time the effect of SMC on the human infectious reservoir for malaria.
- The trial uses measurements of drug plasma concentrations as direct measurements of the effective doses of SMC drugs that are achieved.

For peer review only - http://bmjopen.bmj.com/site/about/guidelines.xhtml

- Potential impact on SMC uptake due to presence of study teams is mitigated by a cross-sectional study survey outside of the study area.
- Study conduct is limited to a single study site.

Introduction

a. Background

Efforts to reduce the global incidence of malaria are failing; there were an estimated 247 million cases of malaria worldwide in 2021, up from 230 million in 2015 (1). With the threat that control efforts are further compromised by insecticide resistance and parasite resistance to first-line drugs, there is a clear need for interventions that reduce the clinical burden of malaria and onward parasite transmission (2).

One recent intervention that has the potential to profoundly reduce the incidence of (severe) clinical disease is seasonal malaria chemoprevention (SMC) (3). SMC provides antimalarials to the most vulnerable populations during the peak of malaria transmission season with the aim to reduce disease burden in this high-risk population. To date, SMC has been administered to millions of African children (4, 5). Although most commonly given to children below the age of 5 years, SMC is recommended for all children in age groups that are at high risk of severe malaria (6). The recent World Health Organization (WHO) guidelines of the year 2022 offer increased flexibility in defining the specific number of monthly cycles and the target age group. In Burkina Faso, SMC is currently administered over 4 rounds to all children aged 3-59 months in July, August, September, and October. These SMC rounds cover the peak of the malaria transmission season that follows seasonal rains that typically peak in July-September. Several countries in the Sahel region, including Burkina Faso, are considering extending the target age range of SMC to all children under the age of 10 years.

Whilst SMC is highly efficacious in research settings, reducing morbidity by up to 60% (5, 7), effect sizes may be lower when SMC is rolled out programmatically (5, 8, 9). A cascade in efficacy decay is regularly observed for efficacious interventions; for SMC this efficacy decay may be related to coverage, compliance (at multiple levels), drug absorption and parasite resistance to SMC drugs (**Figure 1).** A large study that assessed plasma levels of SMC drugs in children in Niger reported evidence for incomplete adherence or drug absorption in >80% of children (9). The choice of drug regimen and parasite resistance to drug components may also play important roles. SMC with the conventional drug combination of sulfadoxine-pyrimethamine plus amodiaquine (SPAQ) is not always effective at clearing parasites: up to 46% of children under the age of 5 years may carry parasites at the end of the transmission season (10, 11). Recently, longitudinal studies in Burkina Faso and The Gambia observed that a large proportion of SMC-targeted children either remained *P. falciparum* infected or became re-infected shortly after receiving SMC with SPAQ (11, 12).

Parasite resistance to the SPAQ antimalarial combination may play a role in defining its anti-infection effects. Resistance to SP is strongly associated with the presence and number of point mutations in dihydropteroate synthase (dhps; e.g. mutations 437Gly and 540Glu) and dihydrofolate reductase (dhfr; e.g. 51Ile, 59Arg, and 108Asn) genes (13, 14). The prevalence of these core mutations and the number of additional mutations in dhps and dhfr are rising across Africa. In East-Africa, quintuple

For peer review only - http://bmjopen.bmj.com/site/about/guidelines.xhtml

mutations in dhfr-dhps are common although a single study suggests that SMC with SPAQ retains protective efficacy (15). Compared to resistance markers for SP, the molecular mechanisms underlying AQ resistance are less well understood and molecular markers for AQ have limited predictive value.

AQ is metabolically activated, primarily by Cytochrome P450 isoenzyme 2C8 (CYP2C8), into desethylamodiaquine (DEAQ). The T_{max} for AQ is around 2 days (Interquartile Range (IQR) 1-3 days) and drug concentrations wane rapidly between days 3 and 7. DEAQ concentrations peak ~2 days after AQ and the terminal half-life of DEAQ (~196.4 h) is approximately four-fold longer than that for AQ (16). AQ resistance associates with variant haplotypes in chloroquine transporter (crt; e.g. 72Cys-73Val-74Ile-75Glu-76Thr) and multidrug resistance gene 1 (mdr1; e.g. encoding Tyr at codons 86, 184, and 1246) (17) but better markers may become available.

In addition to imperfect efficacy against (incident) asexual-stage parasites, SPAQ may also be unable to prevent parasite transmission shortly after treatment. Gametocytes typically persist for several weeks after SPAQ (18) with conflicting findings on gametocyte infectivity after SP treatment. *In vitro* and *in vivo* studies have demonstrated considerable gametocyte sterilization by SP where gametocytes are detectable but no longer (as) infectious (19, 20). However, clinical trials in naturally infected gametocyte carriers demonstrated that SPAQ is ineffective in clearing gametocytes and individuals may remain infectious to mosquitoes for several weeks after treatment (21, 22). Adding single low-dose primaquine rapidly prevented transmission shortly after SPAQ (21).

With SMC being deployed at community level, this intervention has the potential to reduce community transmission and thereby reduce the force of infection experienced by the general population. Recent work directly assessing the infectivity of parasite carriers to mosquitoes identified school-age children (aged 5-15 years) as important reservoirs of infection (23-26). This population is less likely to become symptomatic upon infection and supports high parasite densities and transmissible gametocytes (23-26). Younger children are comparatively less important because their infections are more likely to receive treatment that prevents chronic parasite carriage and due to their small body size and high bednet coverage, mosquito exposure can be relatively low (27). In contrast, school-age children are heavily exposed, currently under-targeted with conventional interventions (e.g. have lowest bednet coverage) and carry the highest densities of gametocytes (23, 28). Whilst less at risk of severe clinical disease, also school-age children may suffer from consequences of parasite carriage that are often overlooked, including haemolysis, increased susceptibility to bacterial infections (29) and reduced cognitive function (28); also this age group would thus likely benefit from interventions that reduce this parasite carriage. Extending the target age range for SMC from all children below 5 years of age to all children below 10 years of age will thus increase the population experiencing direct personal benefits from treatment and may also potentially reduce community-wide transmission by covering a large proportion of the human infectious reservoir for malaria. Further extending the age range for SMC to include all children below 15 years of age is currently not considered by NMCPs.

Recent observations lead to questions related to the reasons for the continued high infection rates in the SMC-targeted population and the potential impact of SMC when the age range is extended to populations that are important to fuelling transmission to the wider community.

b. Rationale

For peer review only - http://bmjopen.bmj.com/site/about/guidelines.xhtml

It is currently unclear whether failure of SMC with SPAQ to prevent parasitaemia is associated with i) imperfect adherence; ii) parasite resistance to the drug components, iii) active drug concentrations that are achieved in SMC participants, and/or iv) consistent exposure to reinfection. It is also unclear whether extending the targeted age-range will reduce the infectious reservoir for transmission to a level where community-benefits may be expected. Lastly, factors that determine efficacy decay are currently poorly understood. Quantifying each of these elements should contribute to understanding the effectiveness of SMC in programmatic settings.

c. Objectives

This study aims to collect detailed information on parasite carriage, gametocyte carriage and infectivity in children targeted and untargeted by SMC in Burkina Faso. The impact of expanding SMC to include children up to 10 years of age on reducing the human infectious reservoir and factors driving imperfect anti-parasite or anti-gametocyte effects of SMC will be assessed. The infectious reservoir will be examined using a combination of gametocyte density estimates in the entire population and mosquito membrane feeding assays in a selection of participants.

Our specific aims are to:

i. Compare SMC effectiveness in children aged 3-59 months in preventing clinical malaria and asymptomatic parasite carriage when implemented by the NMCP or when implemented in a research context where all doses are directly observed.
ii. Evaluate the effectiveness of SMC to reduce malaria infection prevalence in 5–9-year-olds.
iii. Quantify the infectious reservoir and the contribution of different age groups to transmission with conventional SMC (<5 years) and extended SMC (<10 years).
iv. Determine the impact of drug resistance (by molecular typing of *dhps*, *dhfr, pfcrt* and *pfmdr1*) and drug absorption on SMC efficacy.
v. Understand social barriers and enablers interfering with SMC efficacy and how SMC uptake is related to health equity with special attention to gender inequalities.
vi. Quantify SMC efficacy decay under programmatic conditions and key drivers of this decay.

Methods and analyses

a. Trial Design:

The study is designed to evaluate SMC effectiveness and consists of three study arms, with the first arm receiving an intervention that is implemented as part of routine control. This study involves an operational evaluation of a modified existing intervention delivered in independent clusters. The project and its implementation are prepared in direct interaction with the Burkina Faso MoH and NMCP to tailor data collection to local needs. There will be three study arms:

Study arm 1 (control): SMC in children under the age of 5 years, implemented by the MoH; the first dose is given under supervision but there is no directly observed treatment (DOT) for the full course of SMC.
Study arm 2 (intervention): SMC in children under the age of 5 years, with DOT for the full course of SMC.
Study arm 3 (intervention): SMC in children under the age of 10 years, with DOT for the full course of SMC.

For peer review only - http://bmjopen.bmj.com/site/about/guidelines.xhtml

SMC consists of SP and AQ administered daily for three days (30). SP is given on the first SMC day only while AQ is given daily over three days. In total 4 rounds of SMC are conducted during the peak malaria transmission season from July to October, separated by approximately 30 days, concurrently with programmatic interventions in study arms 2 and 3.

Each study arm contains 61 to 62 clusters; each cluster consists of 3 compounds where each compound has at least one child aged 3-59 months and one child aged 5-9 years (185 compounds per arm; 555 compounds in total) (**Figure 2**). The total number of enrolees per arm is the primary statistical consideration; cluster size (3 compounds per cluster) was decided upon based on prior experience (ref) and logistical feasibility. The cluster design is taken into consideration in statistical analyses. All age groups will take part in the evaluation of the study to assess the impact of the intervention on the infectious reservoir in the community and compare parasite carriage in targeted and untargeted populations. To detect possible behavioural contamination and differences in SMC uptake or adherence in study arm 1 compared to what would be observed if there would be no study team present, a single cross-sectional survey will take place 7 days after the last (4th) round of SMC outside the study area (2-5 kilometres from the nearest enrolled study compound). SMC coverage is assessed by questionnaire and sample collection to determine plasma levels of SMC drugs and parasite carriage.

This protocol was developed following SPIRIT reporting guidelines (31).

b. Endpoints:

The primary endpoint is parasite prevalence by quantitative Polymerase Chain Reaction (qPCR) at the end of the malaria transmission season in age groups targeted by SMC. This endpoint will be compared between study arms 1 and 2 (in children aged 3-59 months) and arms 2 and 3 (in children aged 5 years-9 years). Additional secondary and exploratory endpoints will be assessed (**Table 1**).

Table 1. Study primary, secondary and exploratory endpoints.

Primary endpoint

- Parasite prevalence by qPCR at the end of the transmission season in age groups targeted by seasonal malaria chemoprevention.

Secondary endpoints

- Parasite prevalence by qPCR at the end of the transmission season in all age groups (between arm comparison)
- Parasite prevalence by microscopy prior to SMC rounds 2, 3 and 4 in SMC-targeted age groups (between arm comparison)
- Rate of re-infection with *P. falciparum* at weeks 3, 4 and 5 after the last round of SMC, assessed in SMC-targeted age groups *(*between arm comparison*)*

For peer review only - http://bmjopen.bmj.com/site/about/guidelines.xhtml

- Gametocyte prevalence by quantitative reverse transcriptase (qRT)-PCR at weeks 3, 4 and 5 after the last round of SMC, assessed in SMC-targeted age groups (between arm comparison)
- Gametocyte prevalence by qRT-PCR at the end of the transmission season in all age groups (between arm comparison)
- Gametocyte prevalence by qRT-PCR at the end of the transmission season in age groups targeted by SMC (comparison between arms 1 and 2 (in children aged 3-59 months) and arms 2 and 3 (in children aged 5-9 years))
- Plasma levels of AQ and DEAQ after the 4th round of SMC in children aged 3 months-9 years (between arm comparison and comparison with control area)
- Incidence of clinical malaria captured during passive-case detection (between arm comparison)

Exploratory endpoints

- Infectivity to mosquitoes, defined as the percentage of infected mosquitoes, in relation to gametocyte density and plasma drug levels of AQ and DEAQ (across arms assessment)
- Size and age-distribution of the infectious reservoir for malaria, defined as the likelihood that a mosquito becomes infected with malaria parasites after feeding on a population member (between arm comparison)
- Prevalence of drug resistance markers in infected children aged 3 months-9 years assessed post each round of SMC (between arm comparison)
- Description of perceived social barriers to SMC uptake
- Quantification of SMC efficacy decay under programmatic conditions

c. Study sites:

The study site is in Saponé Health District (45 km south-west of Ouagadougou) and the study will benefit from historical records on malaria burden in this area and research infrastructure that was established in previous joint research projects (24, 32, 33). Up-to-date census data will be used prior to the study to select eligible compounds for enrolment.

d. Study population:

The study population will be all members of 555 compounds (61 to 62 clusters per study arm; each cluster consisting of 3 compounds) in Saponé Health District, Burkina Faso. Clusters are randomized over 3 study arms. Compound members of all ages will be included in study procedures (e.g. evaluation of the intervention) but only children will be targeted with the intervention. Based on census data, and recent studies in the same study area, we expect an average of 1.5 children aged <5 years and 1.5 children aged 5-9 years per compound (4.5 children aged <5 years and 4.5 children aged 5-9 years per cluster, respectively).

For peer review only - http://bmjopen.bmj.com/site/about/guidelines.xhtml

e. Study duration:

Total duration of the trial is 13 months, from July 2023 to July 2024.

f. Inclusion and exclusion criteria:

The inclusion and exclusion criteria (**Table 2**) for the evaluation trial are broad to ensure generalizability of the collected data and results.

Table 2: Inclusion and exclusion criteria for the evaluation trial and for the qualitative study on SMC acceptability and uptake

**Compounds are eligible for participation in the trial if**	there are at least 1 child aged <5 years and 1 child aged 5-9 years permanent residents of the compound and willing to participate.
	residents are willing to provide repeated blood samples up to a total volume of 16mL during the entire study period (maximum of 4mL for children aged <5 years).
	the eligible individual or caregiver(s) provide informed consent and additional assent is provided from minor participants aged 12-19 years.
**Household members are excluded from participation in the trial in case of**	any (chronic) illness that requires continuous care.
	current participation in malaria vaccine or drug trials or participation in vaccine trials in the last 2 years.
	ineligibility for SPAQ administration according to NMCP guidelines (only for children).
**Community members are eligible for participation in the qualitative study if they are**	a resident of a household participating in the trial.
	caregiver of a child eligible to SMC.
	20 years of age or older.
**Health care workers are eligible for participation in the qualitative study if they are**	serving as a health care worker delivering SMC intervention.
	serving as health staff in the study zone.
**Community members are excluded from participation**	not residing in the household during SMC preceding the qualitative survey.

For peer review only

in the qualitative study if they are

g. Recruitment:

Following community meetings where study objectives and procedures are explained study teams visit individual households to explain study objectives and procedures for a second time - this time to the head of household and all household members of assenting or consenting age. Study information is presented orally and print outs in French and/or a local language are provided. The voluntary nature of study participation and the right to withdraw at any time are emphasised. It is explained that refusal to participate will not affect access to SMC or any other medical treatment. All household members are encouraged to ask questions prior to signing informed consent (aged >19 years) or assent (aged 12-19 years). Forms and participant information sheets are included as online supplemental material 1.

h. Patient and public involvement

One month prior to the study roll-out community meetings were organized to discuss study objectives and procedures. Special emphasis was given to participant-facing activities, including informed consent. Study findings are presented in community meetings and discussed in depth with community-based opinion leaders.

i. Sample size:

Clusters

Evaluable study clusters will comprise 3 compounds and have between 4-5 children below 5 years of age (eligible for conventional SMC in all study arms) and similar numbers of children aged 5-9 years (eligible for extended SMC in arm 3). We assume ~40% parasite prevalence detected by qPCR at the end of the transmission season in children aged 5-9 years without SMC and 16% parasite prevalence in children aged <5 years under programmatic SMC (Collins *et al. submitted*). When assuming a reduction to 25% parasite prevalence in children aged 5-9 years when included in SMC, a sample size of 55 clusters per arm (x3 compounds per cluster and an estimated total 6 children in this age group per cluster) will achieve 87% power to detect this difference at an alpha of 0.05 and a coefficient of variation of 0.3 (Collins *et al. submitted*). This sample size will give over 80% power to detect a reduction of parasite prevalence in directly observed SMC to 7% as compared to 16% under programmatic SMC, in children aged <5 years. The total number of compounds per arm has been increased to 62 to mitigate the effects of non-compliance and drop-outs and for logistical reasons, such entire villages are included. We will thus enrol a total of 185 clusters over 3 arms in a ratio of 1:1:1 with 555 compounds in total. We anticipate that this will include a minimum of 720 children aged <5 years and 720 children aged 5-9 years (based on recent census data). The cluster-design of the intervention and evaluation will be accounted for in the statistical analysis.

Human infectious reservoir assessments

For assessments of the human infectious reservoir, we use a combination of direct measurements of transmission by mosquito feeding assays and imputation of transmissibility based on gametocyte

For peer review only - http://bmjopen.bmj.com/site/about/guidelines.xhtml

density(25). The reason for a partial reliance on imputation is that the intervention study is too large in scope and the time-window for mosquito feeding experiments is too narrow (i.e. mosquito feeding is ideally done within weeks after SMC) to assess a direct impact of SMC on gametocyte infectivity with a conventionally powered comparison between arms. We will thus use a combination of methodologies where we quantify the association between gametocyte density and infectivity in the absence of treatment, demonstrating consistency across settings (23, 24, 34) and impute mosquito transmission from gametocyte density (23, 25). Direct comparisons of the association between gametocyte density and mosquito infection rates in mosquito feeding assays can demonstrate whether imputation is indeed acceptable across populations (23) or whether there are subpopulations with a different association (for example due to gametocyte-sterilizing effects of antimalarials) (35). Based on these insights direct mosquito feeding assays (DMFAs) will be performed on gametocyte positive individuals with and without prior SMC and mosquito infection rates for a given gametocyte density will be compared (35). Since we hypothesize that SPAQ may permanently sterilize gametocytes, any reduced infectivity of gametocytes will remain apparent even after drug levels have waned (i.e. the gametocytes are permanently damaged by drug exposure)(19). We can efficiently assess this possible sterilizing effect of SPAQ by preferentially recruiting children who are gametocyte positive at the start of SMC and invite them for mosquito feeding post SMC. This will be done in children aged 5-9 years only who carry the highest parasite and gametocyte densities; this also avoids phlebotomy from the youngest children. We anticipate a median gametocyte density of ~14 gametocytes/µL (IQR 1.8-44.2) as observed post-treatment(20, 36). Whilst the number of feeding assays will be informed by feasibility (e.g. mosquito husbandry), we provide an indication of study power. When recruiting 100 children who received SPAQ (arm 3) and 100 children who did not (arms 1 and 2) and dissecting 40 mosquitoes per experiment, simulations show we would have >90% power to detect a 70% reduction in infectivity (37).

Outside study area survey

To give a broad indication of the potential influence the presence of the study team may have on the operational delivery of SMC in arm 1, we will perform a single cross-sectional survey outside the study area. The survey is not part of any main comparisons, and an exact sample size justification is complicated. Estimates of adherence are based on questionnaire data and supported by measurements of drug levels in plasma. In arm 2 and 3 (DOT for the full course of SMC), the median level of DEAQ at day 4 following the last dose of AQ is expected to be 550 ng/mL (IQR 350-850) (16). Based on a large assessment of reported adherence and measured AQ/DEAQ plasma levels following SMC in Niger, we anticipate <50% of children show complete adherence in arm 1 and the control village and >20% of the children in these populations may fail to take any dose(9). If we conservatively assume that this results in a reduction of median DEAQ levels to <400ng/mL (IQR 200-700), we can estimate the power to detect this difference when enrolling 120 children in the SMC-targeted age range in the control area. For this, we assume that these DEAQ levels stem from right-skewed (log-normal) distributions; giving median log DEAQ concentrations of 6.31 (IQR 5.86-6.75) for DOT and 5.99 (IQR 5.30-6.55) for non-DOT. If we further assume that the distribution of the logged values is normally distributed, we can estimate the standard deviation through the IQR as IQR/1.349, giving standard deviations of 0.93 and 0.66, respectively. Using a sample size of 120 per group, and a Welch's two sample t-test for testing the differences between the log DEAQ concentrations, we estimate a power of 86.3% to detect these differences. Based on these assumptions, we are confident that sampling 120

For peer review only - http://bmjopen.bmj.com/site/about/guidelines.xhtml

children <5 years of age will allow meaningful assessments. As indicated above, this sample size estimation is not relevant for any of the main comparisons in the study but merely used to ensure informative sampling and avoid exposing an unnecessarily large population to the small discomfort of a single finger prick sample.

j. Study procedures

Seasonal malaria chemoprevention

SMC drug administration uses SPAQ at the conventional dosing (supplemental material 2). This medication is provided by the MoH. SMC is administered by MoH teams in arm 1 (no DOT) and by dedicated research staff in arms 2 and 3 (DOT). Clinical malaria cases will receive conventional first-line treatment with artemether-lumefantrine (AL) according to national treatment guidelines when they attend study clinics.

Scheduled surveys

Scheduled surveys will take place in each study arm and are designed and timed for several outcome measures (**Figure 3**):

1. A survey prior to SMC roll-out (baseline survey) is used to determine parasite carriage and to collect parasite material for genotyping infections and comparing parasite genotypes with parasites detected following SMC.

2. A mid-study survey aims to assess parasite carriage at different time-points following SMC and relate this to time since SMC and SMC plasma levels. These surveys are scheduled 3, 4 or 5 weeks after the last round of SMC. Each compound will participate in one of these surveys; each cluster has 3 compounds and one of these compounds is randomly selected to participate in the first, second or third mid-study survey. These surveys will include all compound members (all age groups) to also allow comparisons of gametocyte carriage and transmission potential between arms.

3. The end of study survey (endline survey) will target all age groups in the study households to determine whether parasite carriage and gametocyte carriage are reduced both in the SMC targeted populations and in the wider community. This survey will also provide information on the primary endpoint (parasite carriage by qPCR at the end of the transmission season). During these surveys, all individuals will have their axillary temperature measured and a finger prick blood sample taken onto a dried blood spot and into an EDTA-coated microtainer for collection of plasma and nucleic acids for later RNA- and DNA-work. In case of measured fever, the same finger prick blood sample will also be used for malaria diagnosis by conventional rapid diagnostic test to guide treatment according to national guidelines.

4. In addition to these surveys, there will be assessments of drug metabolites (shortly after the last round of SMC). Screening for parasite carriage by microscopy in children aged 5-9 years is scheduled on the first day of SMC rounds 2 to 4. Microscopy-positive gametocyte carriers will be prioritized for mosquito feeding assays (DMFA) to assess infectivity. Other microscopy-positive non-gametocyte carriers may be included for DMFA if mosquito numbers allow. DMFA will occur within 21 days after the first dose of SMC.

Passive case detection

For peer review only - http://bmjopen.bmj.com/site/about/guidelines.xhtml

In addition to these scheduled surveys and throughout the study period there is malaria passive case detection in all four health facilities that are accessed by the selected study population. Participants under 10 years will receive ID cards to facilitate identification. Trained clinical personnel are available 24/7. In case of clinical malaria, defined as microscopy-confirmed malaria infection with measured or reported fever, treatment is provided with AL, according to MoH policy. Before treatment, a finger-prick blood sample is taken for parasitological and drug metabolite assessment. Clinical incidence will be expressed per person-month in the target population (<5 years old in arms 1 and 2 and <10 years old in arms 2 and 3).

Adherence to and perceptions about SMC strategy

Selected community members and health workers will be invited for participation in the qualitative part of the study. The qualitative component of this study is designed to understand potential factors that influence SMC uptake and effectiveness, including:

1) SMC coverage – defined as proportion of eligible children reached by the four rounds of SMC;

2) Health workers compliance to MoH guidelines (proportion of children receiving a directly observed treatment for the first dose of SMC drugs as recommended by the treatment guidelines);

3) Caregivers' compliance – defined as the proportion of children receiving the correct dosing regimen (number of tablets and duration);

4) perceptions of the SMC program from caregiver and health system perspectives.

Perceptions will include the perceived benefits and ease of participation of SMC, as well as perceived utility of additional interventions (e.g. long lasting nets, indoor residual spraying, personal protective measures) when adhering to SMC.

For participation in the qualitative study on SMC adherence and perceptions, there are distinct inclusion and exclusion criteria for community members and health workers (**Table 2**).

Throughout the study, data on compliance to SMC treatment guidelines from caregiver and healthcare worker perspectives are collected in arm 1. Whether first dose was directly observed and whether the full treatment course was administered to the child after each round on SMC is documented. After SMC round 4 a cross-sectional survey will be carried out. For this, individual interviews of caregivers of children targeted or not by the current SMC strategy will be conducted. Further, within each of the study arms, between 6-8 individuals will be recruited for focus group discussions.

Key informant interviews (KIIs) and in-depth interviews (IDIs) will be carried out. For the KIIs, participants will be recruited through purposive sampling to include stakeholders within the health system involved in SMC implementation. The IDIs will be conducted among 5-10 key informants per each of the study arms. These individuals will also be purposively selected aiming at including community leaders, professionals, or residents who have first-hand knowledge about the community.

k. Laboratory assessments

Parasitology

For peer review only - http://bmjopen.bmj.com/site/about/guidelines.xhtml

Thick and/or thin blood films for parasite counts will be obtained at the timepoint of the first SMC dose for rounds 2-4, during clinical malaria episodes and during cross-sectional sampling to select study participants for assessment of infectivity by DMFA.

Further, parasite DNA and RNA will be extracted from whole blood samples in EDTA tubes and tested using qPCR that targets varATS and detects all *P. falciparum* parasites (asexual parasites and gametocytes). Parasite prevalence and density will be assessed as intervention outcome measures, as previously done for community interventions (32).

P. falciparum gametocytes will be quantified by quantitative reverse transcriptase PCR targeting female (Ccp4) and male gametocytes (PfMGET) (38, 39). For a subset of infections, multiplicity of infection and the genetic relatedness of infections that are detected in the same study participant over time will be determined (e.g. AMA-1 amplicon deep sequencing). This amplicon sequencing will be performed in a selection of DNA samples that are parasite positive by varATS qPCR and for whom re-infection or infection persistence is suspected.

Assessments of molecular markers of resistance will follow established protocols (14). Nucleic acids will be extracted with commercial kits [Qiagen] and stored at –20°C until use. Three dual-labelled probes were designed to detect three crt genotypes at codons 72–76 (encoding Cys-Val-Met-Asn-Lys [CVMNK], CVIET, and SVMNT). Laboratory isolates *P. falciparum* 3D7, Dd2, and 7G8 will be used as positive controls for the CVMNK, CVIET, and SVMNT haplotypes, respectively. qPCR amplification will be done in a single well to maximise throughput using a thermocycler (BioRad). All samples will further be tested for mdr1, dhfr, and dhps. For this, all three genes will be amplified by nested PCR using previously described methods (Pearce et al, 2003) and polymorphisms identified by direct sequencing of amplified products (BigDye Terminator v3.1 cycle sequencing kits and ABI 3730 sequencer; ThermoFisher Scientific) and data will be analysed using Geneious v10.1.3 (Biomatters, San Diego, CA, USA).

<u>Pharmacology</u>

AQ and DEAQ concentrations will be quantified from plasma samples and filter paper spots. Blood concentrations are measured within 6 months of sample collection using a liquid chromatography tandem mass spectrometry assay (9, 40).

<u>Serology</u>

Serological analysis of these samples will be conducted using bead-based assays to assess:

- presence of biomarkers for correlates of infection and infectivity (i.e. proteins associated with inflammation), or parasite density (HRP2)(41).
- antibody responses to gametocyte and asexual stage proteins in established multiplex platforms(41). Antigens will include but not be restricted too Pfs230, Pfs48/45 for sexual stage proteins to assess the influence on transmission to mosquitoes and PfAMA, PfMSP-1 and Pf ETRAMP 5 to assess the effect of interventions on the development of humoral immunity.

<u>Mosquito feeding assays</u>

For each assessment of infectivity, 2 mL of heparinized venous blood will be drawn from the study participant selected for DMFA. At the insectary, ~70 *Anopheles gambiae s.s.* mosquitoes will be fed on the subjects' blood for 15-20 minutes using a water-jacketed feeder system (32). Mosquitoes will

For peer review only

be dissected in 0.5% mercurochrome on the 7th day after the feeding assay for the prevalence of mosquitoes with oocysts and quantification of oocysts.

l. Randomization

The unit of randomization is the cluster; each cluster comprises of 3 compounds. To account for spatial differences in malaria exposure, distance from health facilities, and treatment seeking behaviour, neighbouring clusters are grouped before randomization. Clusters are randomly allocated to one of the three study arms by a computer-generated algorithm and stratified by village. In this way clusters are spatially 'matched' and are thus likely to experience similar malaria exposure. In total, the randomization resulted in 185 compounds (61 to 62 clusters with 3 compounds each) in each arm.

m. Analyses plan

Primary endpoint:

The primary endpoint on parasite prevalence (by qPCR) at the end of the transmission season in age groups targeted by SMC will be compared in children aged 3-59 months in study arms 1 and 2 and in children aged 5 to 9 years in study arms 2 and 3. Parasite prevalence by qPCR will be used as binary variable with all parasite densities above 100 parasites/mL (0.1 parasite/µL) being classified as parasite positive. Parasite prevalence is compared between arms by logistic regression; models will account for clustering by including compound ID as a random intercept and village as a fixed effect.

Secondary and exploratory endpoints:

A complete statistical analysis plan (SAP) has been developed for this trial and is provided in the online supplemental material 3. All parasite prevalence related endpoints will be compared as described above for the primary endpoint. Gametocyte prevalence related endpoints will be presented as prevalence estimate with 95% confidence interval.

Ethics and dissemination

The study has been reviewed and approved by the London School of Hygiene and Tropical Medicine (LSHTM) (Review number: 29193; Version 2.0, July 2023) and Burkina Faso National Medical Ethics Committees (Deliberation N° 2023-05-104). Data will be stored on secure servers at GRAS and shared with primary project partners as de-identified. A study participant identification link log is available at the investigators' site. Data will be deposited with publications and uploaded to shared data repositories for broader use by the scientific community (e.g ClinEpi DB).

The findings of the study will be shared with the community via the local opinion leaders. The project is followed by a community meeting to present some of the preliminary findings. Disease occurrence data will be shared with the Ministry of Health. The findings will also be shared in peer-reviewed publications irrespective of final results.

Trial status

For peer review only

For peer review only - http://bmjopen.bmj.com/site/about/guidelines.xhtml

This study is registered on ClinicalTrials.gov #NCT05878366.

ACKNOWLEDGMENTS

The authors thank the Ministry of Health and National Malaria Control Program from Burkina Faso and staff at the study sites for support, as well as the research nurses, laboratory and administrative staff involved in the study.

Footnotes

MM, AB and MG are joint first authors. CD and AT are joint senior authors.

AUTHOR CONTRIBUTIONS

ABT, TB, and CD developed and conceived the initial design of the study, secure funding and contributed significantly to writing this protocol. MM, AB, MG, SSS, JB, AO, DH and JBY contributed to study design and protocol development. AB, JBY and ABT coordinated the implementation of the study. AB, JBY, SSS, AO, IS, AZO, ES, INO, SBS, DH run all field operations and provided extensive information on local context and logistics. IS and LG provided drug resistance expertise. DH and JS provided expertise on qualitative methods. IB provided spatial analysis expertise. JR, JB and SS provided statistical expertise. SK and RH provided pharmacological expertise. MM, MG, TB, CD and ABT wrote the first draft of the manuscript. All authors reviewed and approved the final draft of the manuscript.

FUNDING STATEMENT

This work is supported by Bill and Melinda Gates Foundation grant (INV-053846). The funders, Bill and Melinda Gates Foundation, were involved in study design, but had no role in data collection, analysis, interpretation or reporting.

COMPETING INTERESTS

None declared.

REFERENCES

1. Organization WH. World malaria report 2022. 2022. Contract No.: CC BY-NC-SA 3.0 IGO.
2. Das S, Saha B, Hati AK, Roy S. Evidence of Artemisinin-Resistant Plasmodium falciparum Malaria in Eastern India. N Engl J Med. 2018;379(20):1962-4.
3. Partnership A-S. Effectiveness of seasonal malaria chemoprevention at scale in west and central Africa: an observational study. Lancet. 2020;396(10265):1829-40.
4. Ashley EA, Poespoprodjo JR. Treatment and prevention of malaria in children. Lancet Child Adolesc Health. 2020;4(10):775-89.
5. Cairns M, Ceesay SJ, Sagara I, Zongo I, Kessely H, Gamougam K, et al. Effectiveness of seasonal malaria chemoprevention (SMC) treatments when SMC is implemented at scale: Case-control studies in 5 countries. PLoS Med. 2021;18(9):e1003727.
6. Organization WH. Updated WHO recommendations for malaria chemoprevention among children and pregnant women. Geneva, Switzerland; 2022.
7. Cissé B, Ba EH, Sokhna C, JL ND, Gomis JF, Dial Y, et al. Effectiveness of Seasonal Malaria Chemoprevention in Children under Ten Years of Age in Senegal: A Stepped-Wedge Cluster-Randomised Trial. PLoS Med. 2016;13(11):e1002175.

For peer review only - http://bmjopen.bmj.com/site/about/guidelines.xhtml

8. malERA. malERA: An updated research agenda for health systems and policy research in malaria elimination and eradication. PLoS Med. 2017;14(11):e1002454.
9. Ding J, Coldiron ME, Assao B, Guindo O, Blessborn D, Winterberg M, et al. Adherence and Population Pharmacokinetic Properties of Amodiaquine When Used for Seasonal Malaria Chemoprevention in African Children. Clin Pharmacol Ther. 2020;107(5):1179-88.
10. Chandramohan D, Zongo I, Sagara I, Cairns M, Yerbanga RS, Diarra M, et al. Seasonal Malaria Vaccination with or without Seasonal Malaria Chemoprevention. N Engl J Med. 2021;385(11):1005-17.
11. Collins KA, Ouedraogo A, Guelbeogo WM, Soulama I, Ouattara MS, Sombie S, et al. Active Screening and Treatment Significantly Reduces the Infectious Reservoir of Malaria in Burkina Faso: Results from a Randomised Trial. and Treatment Significantly Reduces the Infectious Reservoir of Malaria in Burkina Faso: Results from a Randomised Trial. . Available at SSRN: https://ssrncom/abstract=4585247. 2023.
12. S. Soremekun BC, A. Nyassi, H. Soumare, B. Etoketim, M. Ndiath, J. Bradley, U. D'Alessandro, T. Bousema, A. Erhart, M. Moreno, C. Drakeley. Household-level effects of seasonal malaria chemoprevention in the Gambia. Research Square. 2023.
13. Naidoo I, Roper C. Mapping 'partially resistant', 'fully resistant', and 'super resistant' malaria. Trends Parasitol. 2013;29(10):505-15.
14. Beshir KB, Muwanguzi J, Nader J, Mansukhani R, Traore A, Gamougam K, et al. Prevalence of Plasmodium falciparum haplotypes associated with resistance to sulfadoxine-pyrimethamine and amodiaquine before and after upscaling of seasonal malaria chemoprevention in seven African countries: a genomic surveillance study. Lancet Infect Dis. 2023;23(3):361-70.
15. Nuwa A, Baker K, Bonnington C, Odongo M, Kyagulanyi T, Bwanika JB, et al. A non-randomized controlled trial to assess the protective effect of SMC in the context of high parasite resistance in Uganda. Malar J. 2023;22(1):63.
16. Anyorigiya TA, Castel S, Mauff K, Atuguba F, Ogutu B, Oduro A, et al. Pharmacokinetic profile of amodiaquine and its active metabolite desethylamodiaquine in Ghanaian patients with uncomplicated falciparum malaria. Malar J. 2021;20(1):18.
17. Humphreys GS, Merinopoulos I, Ahmed J, Whitty CJ, Mutabingwa TK, Sutherland CJ, et al. Amodiaquine and artemether-lumefantrine select distinct alleles of the Plasmodium falciparum mdr1 gene in Tanzanian children treated for uncomplicated malaria. Antimicrob Agents Chemother. 2007;51(3):991-7.
18. Bousema JT, Schneider P, Gouagna LC, Drakeley CJ, Tostmann A, Houben R, et al. Moderate Effect of Artemisinin-Based Combination Therapy on Transmission of Plasmodium falciparum. Journal of Infectious Diseases. 2006;193(8):1151-9.
19. Alkema M, Reuling IJ, de Jong GM, Lanke K, Coffeng LE, van Gemert GJ, et al. A Randomized Clinical Trial to Compare Plasmodium falciparum Gametocytemia and Infectivity After Blood-Stage or Mosquito Bite-Induced Controlled Malaria Infection. J Infect Dis. 2021;224(7):1257-65.
20. Dicko A, Roh ME, Diawara H, Mahamar A, Soumare HM, Lanke K, et al. Efficacy and safety of primaquine and methylene blue for prevention of Plasmodium falciparum transmission in Mali: a phase 2, single-blind, randomised controlled trial. The Lancet Infectious Diseases. 2018;18(6):627-39.
21. Kone A, van de Vegte-Bolmer M, Siebelink-Stoter R, van Gemert GJ, Dara A, Niangaly H, et al. Sulfadoxine-pyrimethamine impairs Plasmodium falciparum gametocyte infectivity and Anopheles mosquito survival. Int J Parasitol. 2010;40(10):1221-8.
22. Mahamar A, Smit M, Sanogo K, Sinaba Y, Niambele SM, Sacko A, et al. Artemether-lumefantrine with or without single-dose primaquine and sulfadoxine-pyrimethamine plus amodiaquine with or without single-dose tafenoquine to reduce Plasmodium falciparum transmission: a phase 2 single-blind randomised clinical trial in Ouelessebougou, Mali.

. Lancet Microbe, accepted for publication2024.

For peer review only - http://bmjopen.bmj.com/site/about/guidelines.xhtml

23. Andolina C, Rek JC, Briggs J, Okoth J, Musiime A, Ramjith J, et al. Sources of persistent malaria transmission in a setting with effective malaria control in eastern Uganda: a longitudinal, observational cohort study. Lancet Infect Dis. 2021;21(11):1568-78.
24. Goncalves BP, Kapulu MC, Sawa P, Guelbeogo WM, Tiono AB, Grignard L, et al. Examining the human infectious reservoir for Plasmodium falciparum malaria in areas of differing transmission intensity. Nat Commun. 2017;8(1):1133.
25. Rek J, Blanken SL, Okoth J, Ayo D, Onyige I, Musasizi E, et al. Asymptomatic school-aged children are important drivers of malaria transmission in a high endemicity setting in Uganda. J Infect Dis. 2022.
26. Staedke SG, Maiteki-Sebuguzi C, Rehman AM, Kigozi SP, Gonahasa S, Okiring J, et al. Assessment of community-level effects of intermittent preventive treatment for malaria in schoolchildren in Jinja, Uganda (START-IPT trial): a cluster-randomised trial. Lancet Glob Health. 2018;6(6):e668-e79.
27. Guelbeogo WM, Goncalves BP, Grignard L, Bradley J, Serme SS, Hellewell J, et al. Variation in natural exposure to anopheles mosquitoes and its effects on malaria transmission. Elife. 2018;7.
28. Cohee LM, Opondo C, Clarke SE, Halliday KE, Cano J, Shipper AG, et al. Preventive malaria treatment among school-aged children in sub-Saharan Africa: a systematic review and meta-analyses. Lancet Glob Health. 2020;8(12):e1499-e511.
29. Chen I, Clarke SE, Gosling R, Hamainza B, Killeen G, Magill A, et al. "Asymptomatic" Malaria: A Chronic and Debilitating Infection That Should Be Treated. PLoS Med. 2016;13(1):e1001942.
30. World Health Organisation. Updated WHO recommendations for malaria chemoprevention among children and pregnant women. 2022.
31. Chan AW, Tetzlaff JM, Altman DG, Laupacis A, Gotzsche PC, Krleza-Jeric K, et al. SPIRIT 2013 statement: defining standard protocol items for clinical trials. Ann Intern Med. 2013;158(3):200-7.
32. Collins KA, Ouedraogo A, Guelbeogo WM, Awandu SS, Stone W, Soulama I, et al. Investigating the impact of enhanced community case management and monthly screening and treatment on the transmissibility of malaria infections in Burkina Faso: study protocol for a cluster-randomised trial. BMJ Open. 2019;9(9):e030598.
33. Barry A, Bradley J, Stone W, Guelbeogo MW, Lanke K, Ouedraogo A, et al. Higher gametocyte production and mosquito infectivity in chronic compared to incident Plasmodium falciparum infections. Nat Commun. 2021;12(1):2443.
34. Bradley J, Stone W, Da DF, Morlais I, Dicko A, Cohuet A, et al. Predicting the likelihood and intensity of mosquito infection from sex specific Plasmodium falciparum gametocyte density. Elife. 2018;7.
35. Bradley J, Soumare HM, Mahamar A, Diawara H, Roh M, Delves M, et al. Transmission-blocking Effects of Primaquine and Methylene Blue Suggest Plasmodium falciparum Gametocyte Sterilization Rather Than Effects on Sex Ratio. Clin Infect Dis. 2019;69(8):1436-9.
36. Stone W, Mahamar A, Smit MJ, Sanogo K, Sinaba Y, Niambele SM, et al. Single low-dose tafenoquine combined with dihydroartemisinin-piperaquine to reduce Plasmodium falciparum transmission in Ouelessebougou, Mali: a phase 2, single-blind, randomised clinical trial. Lancet Microbe. 2022;3(5):e336-e47.
37. Ramjith J, Alkema M, Bradley J, Dicko A, Drakeley C, Stone W, et al. Quantifying Reductions in Plasmodium falciparum Infectivity to Mosquitos: A Sample Size Calculator to Inform Clinical Trials on Transmission-Reducing Interventions. Front Immunol. 2022;13:899615.
38. Schneider P, Schoone G, Schallig H, Verhage D, Telgt D, Eling W, et al. Quantification of Plasmodium falciparum gametocytes in differential stages of development by quantitative nucleic acid sequence-based amplification. MolBiochemParasitol. 2004;137(1):35-41.
39. Meerstein-Kessel L, Andolina C, Carrio E, Mahamar A, Sawa P, Diawara H, et al. A multiplex assay for the sensitive detection and quantification of male and female Plasmodium falciparum gametocytes. Malaria Journal. 2018;17(1):441.

For peer review only - http://bmjopen.bmj.com/site/about/guidelines.xhtml

40. Roh ME, Zongo I, Haro A, Huang L, Some AF, Yerbanga RS, et al. Seasonal Malaria Chemoprevention Drug Levels and Drug Resistance Markers in Children With or Without Malaria in Burkina Faso: A Case-Control Study. J Infect Dis. 2023;228(7):926-35.
41. de Jong RM, Alkema M, Oulton T, Dumont E, Teelen K, Nakajima R, et al. The acquisition of humoral immune responses targeting Plasmodium falciparum sexual stages in controlled human malaria infections. Front Immunol. 2022;13:930956.

For peer review only

For peer review only - http://bmjopen.bmj.com/site/about/guidelines.xhtml

Figure Legends

Figure 1. Cascade in efficacy decay in SMC effectiveness. Coverage, compliance, and drug absorption will all influence the actual efficiency that is observed when SMC is implemented under programmatic conditions[28].DOT: Directly observed treatment.

Figure 2. Study design. The study will be implemented in clusters. Each cluster consists of 3 compounds where each compound has at least one child aged 3-59 months and one child aged 5-9 years. All age groups will take part in the evaluation of the study to assess the impact of the intervention on the infectious reservoir in the community and compare parasite carriage in targeted and untargeted populations. Figure was created with BioRender.com.

Figure 3. Surveys and sampling schedule. Cluster randomization was performed prior to the baseline study. PCD: Passive case detection; PK surveys: Pharmacokinetics analysis; DMFAs: direct membrane feeding assays, done up to 21 days from 1st SMC dose. Adherence to and perceptions about SMC strategy will be collected throughout the study in the three study arms. Figure was created with BioRender.com.

For peer review only

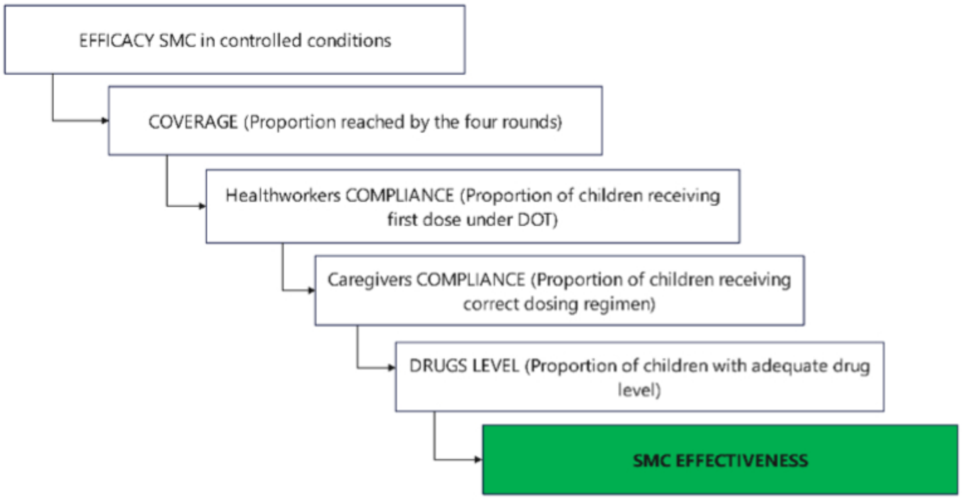

Figure 1. Cascade in efficacy decay in SMC effectiveness. Coverage, compliance, and drug absorption will all influence the actual efficiency that is observed when SMC is implemented under programmatic conditions(28).DOT: Directly observed treatment.

342x185mm (59 x 59 DPI)

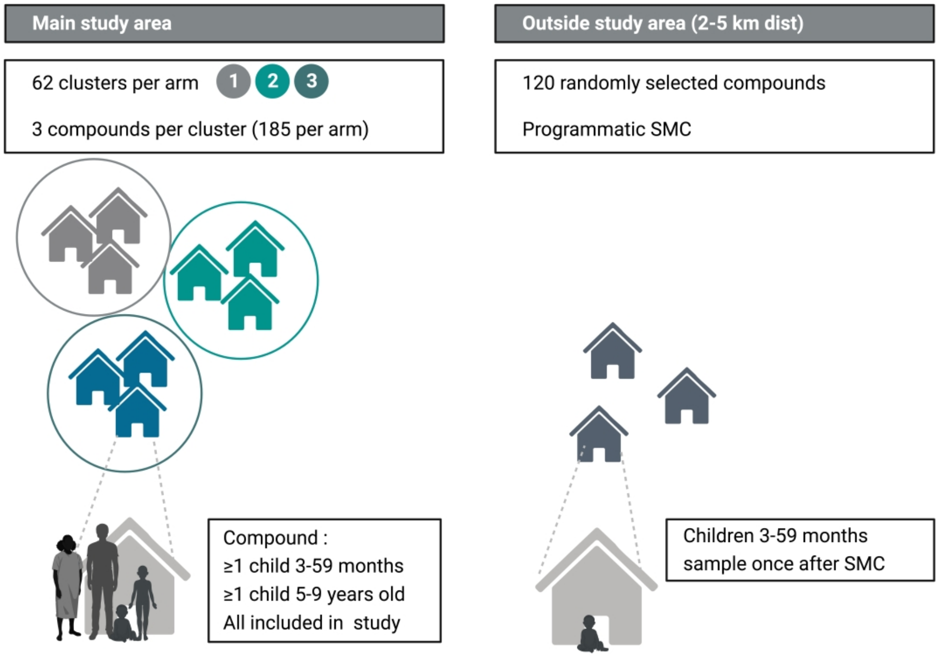

Figure 2. Study design. The study will be implemented in clusters. Each cluster consists of 3 compounds where each compound has at least one child aged 3-59 months and one child aged 5-9 years. All age groups will take part in the evaluation of the study to assess the impact of the intervention on the infectious reservoir in the community and compare parasite carriage in targeted and untargeted populations. Figure was created with BioRender.com.

645x452mm (236 x 236 DPI)

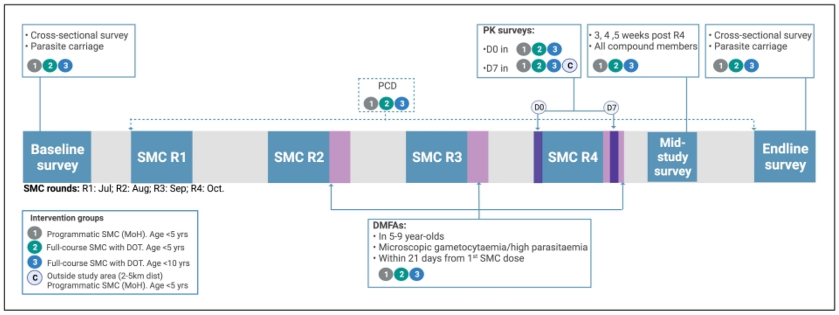

Figure 3. Surveys and sampling schedule. Cluster randomization was performed prior to the baseline study. PCD: Passive case detection; PK surveys: Pharmacokinetics analysis; DMFAs: direct membrane feeding assays, done up to 21 days from 1st SMC dose. Adherence to and perceptions about SMC strategy will be collected throughout the study in the three study arms. Figure was created with BioRender.com.

645x452mm (236 x 236 DPI)

Head of Compound information and Informed Consent Documents

TITLE OF STUDY: INDIE_SMC Understanding and maximizing the community impact of antimalarial treatment

INSTITUTIONS: Groupe de Recherche Action en Santé (GRAS); London School of Hygiene & Tropical Medicine, London, UK (LSHTM); Radboud university medical center, Nijmegen, the Netherlands (RUMC)

PRINCIPAL INVESTIGATORS: Dr. B. Alfred Tiono (GRAS), Prof. Teun Bousema (LSHTM; RUMC), Prof. Chris Drakeley (LSHTM).

SPONSOR DETAILS: London School of Hygiene and Tropical Medicine, London, UK.

Compound ID: _______________

What is the purpose of this study?

Malaria is caused by parasites that are transmitted by mosquito bites and can be cured by early treatment with malaria drugs. When a human has had an infection for some time, the parasites in their body may be picked up by other mosquitoes when they bite, making that mosquito infectious to other humans. This is known as the cycle of transmission – malaria has to be transmitted from human to mosquito to human and so on, to survive. When a person has been infected with malaria a number of times they may be protected against some of the symptoms of the disease. When this protection has developed, the infected individual may not show signs of disease, but the parasite will still be present in the body, and may be capable of infecting mosquitoes.

Seasonal Malaria Chemoprevention (SMC) is one of the tools currently used to combat malaria in children. It involves repeated administrations of sulfadoxine-pyrimethamine plus amodiaquine (SPAQ) to children below the age of 5 years during the rainy season which is the peak transmission period in areas of seasonal malaria transmission. Whilst highly impactful in controlled research settings, the impact of SMC in terms of reducing infection prevalence is limited in real-life settings. We therefore want to do this research to understand what are the factors that are reducing the SMC effects in our population. We would also want to know whether extending SMC to all children below 10 years of age will be beneficial to them and moreover to the wider people in the village.

For this during this research we will compare three groups of population:

1) First group will include children under the age of 5 years receiving the SMC delivered by the local health district as per national guidelines

For peer review only - http://bmjopen.bmj.com/site/about/guidelines.xhtml

2) In the second group children under age of 5 years will receive directly observed treatment for the full (three day) course of SMC.
3) Children in the third group will be under the age of 10 years. They will receive directly observed treatment for the full (three day) course of SMC.

What are the study procedures?
We aim to include 360 compounds from district of Saponé. From each compound, we will select up to 6 individuals including all the children aged below 10 years and over. This selection will be done at random. These selected study participants will be examined for signs of severe disease and other health conditions that may influence the ability to complete the study.
For all people who were selected and want to participate in the study, we will divide compounds in three arms. This division will be done at random (by chance). We will then provide to each child under 10 years an identification card and take pictures that will be attached to this card to help us easily identify them when they come to the health facility. With these cards, study participants have the right to free malaria-related medical care at the health facility. This free care is provided 24 hours per day, 7 days per week for a period of 6-7 months.
The adults will take part to cross sectional surveys on three occasions during which we will ask questions and collect blood sample by finger prick.
The compounds will be visited at least twice every month during the SMC round and after the round to administer study procedures to participants. We will explain in detail the procedures each participant will follow when we discuss the individual informed consent with potential participants.

Voluntary participation
You are free to decide whether you want your compound to be in the study or not. Your decision not to allow your compound to participate to the study will not affect the care your compound members receive at the local health facility any way. Even if you do agree for your compound to enter in the study, you can withdraw your compound from the study at any time without giving any reason.

What discomforts and risks is my compound exposed to?
There is no direct discomfort or risk for your compound to be in the study. You will receive regular home visits over the study period (6-7 months). These visits may disturb your family privacy. However, we will do our best to minimise this and our community health workers will be trained to use discretion to ensure your privacy.

What benefits can I expect from my compound's participation?
You will not receive any payment for your compound being part of the study.

What about the confidentiality of compound data?
The records concerning your compound's participation are to be used only for the purpose of this research project. The names of your compound members will not be used on any report resulting from this study. At the beginning of the study, we will give your compound a study identification number and this number will be used on the study documents. Any information obtained in connection with this study will be kept strictly confidential and under lock and key. Only senior research team members and authorized people such as ethics committee members will have access to your compound's records.

What if I have questions?

For peer review only - http://bmjopen.bmj.com/site/about/guidelines.xhtml

If you have any question concerning this study, do not hesitate to contact the Principal Investigator, Dr Alfred B. Tiono at [redacted] Results from the study will be communicated to your community. In case you want to contact an independent person, not related to the study, about the research study itself, you can contact the Chairman of Ethics Committee, Prof. Fla KOUETA at [redacted]

For peer review only

For peer review only - http://bmjopen.bmj.com/site/about/guidelines.xhtml

Informed consent agreement

I, ____ (NAME OF HOUSEHOLD HEAD), having full capacity to consent to the participation of my compound in the study entitled **" INDIE_SMC Understanding and maximizing the community impact of antimalarial treatment "** conducted by principal investigator Dr. Alfred B. Tiono, acknowledge the implications of voluntary participation, the nature, duration and purpose, methods and means by which it is to be conducted. Harms and risks that can reasonably be expected to be encountered have been explained to me by ____ and are set out in the information document. I have had the opportunity to ask questions about this research and all questions have been answered to my satisfaction. If I have any further questions, I can contact Dr. Alfred B. Tiono (GRAS) at [redacted] I understand that I may at any time during the course of this study revoke my consent and withdraw my compound from the study without prejudice; however, compound members may be asked to undergo further examinations if, in the opinion of the physician, such examinations are necessary for their well-being.

I understand/do not understand the practical implications of this study (circle one)

I approve / disapprove of members of my compound being visited at home during the 6-7 months period of the study. (circle one)

I agree/disagree that my compound could take part in this study (circle one)

Name of Compound Head : ____

Signature : ______________________

Date : ______________________

Fingerprint if unable to sign

****

Name of the Independent Witness: ____

Signature : ___________________________ Date : _____________________

****

Name of person obtaining the consent: ____

Signature : ______________________________ Date : ____________________

For peer review only - http://bmjopen.bmj.com/site/about/guidelines.xhtml

Participant parent/guardian of children in the study area information and Informed Consent Documents

TITLE OF STUDY: INDIE_SMC Understanding and maximizing the community impact of antimalarial treatment

INSTITUTIONS: Groupe de Recherche Action en Santé (GRAS); London School of Hygiene & Tropical Medicine, London, UK (LSHTM); Radboud university medical center, Nijmegen, the Netherlands (RUMC)

PRINCIPAL INVESTIGATORS: Dr. B. Alfred Tiono (GRAS), Prof. Teun Bousema (LSHTM; RUMC), Prof. Chris Drakeley (LSHTM).

SPONSOR DETAILS: London School of Hygiene and Tropical Medicine, London, UK.

Participant ID: ______________

What is the purpose of this study?

Malaria is caused by parasites that are transmitted by mosquito bites and can be cured by early treatment with malaria drugs. When a human has had an infection for some time, the parasites in their body may be picked up by other mosquitoes when they bite, making that mosquito infectious to other humans. This is known as the cycle of transmission – malaria has to be transmitted from human to mosquito to human and so on, to survive. When a person has been infected with malaria a number of times, they may be protected againts some of the symptoms of the disease. When this protection has developed, the infected individual may not show signs of disease, but the parasite will still be present in the body, and may be capable of infecting mosquitoes.

Seasonal Malaria Chemoprevention (SMC) is one of the tools currently used to combat malaria in children. It involves repeated administrations of sulfadoxine-pyrimethamine plus amodiaquine (SPAQ) to children below the age of 5 years during the rainy season which is the peak transmission period in areas of seasonal malaria transmission. Whilst highly impactful in controlled research settings, the impact of SMC in terms of reducing infection prevalence is limited in real-life settings. We therefore want to do this research to understand what are the factors that are hindering the SMC effects in our population. We would also want to know whether extending SMC to all children below 10 years of age will be beneficial to them and moreover to the wider people in the village.

For this during this research we will compare three groups of population:

1) First group will include children under the age of 5 years receiving the SMC delivered by the local health district as per national guidelines.
2) In the second group children under age of 5 years will receive directly observed treatment for the full (three day) course of SMC.

For peer review only - http://bmjopen.bmj.com/site/about/guidelines.xhtml

3) Children in the third group will be under the age of 10 years. They will receive directly observed treatment for the full (three day) course of SMC.

What are the study procedures?
We aim to include 2160 participants (1440 children and 720 adults) in this study.
If you allow your child/ward to be in the study, we will collect a blood sample by finger to assess whether he/she has the germs that cause malaria in his/her blood.
If your child/ward is in the first group, we will again visit him/her during the SMC rounds at four occasions to collect finger prick samples to check whether he/she has germs that cause malaria. During these visits, we will also ask you questions about his/her health status and how the SMC treatment was given. If your child/ward is in the second or third group, we will visit him/her at four occasions during the SMC round to make sure the treatment is correctly taken under supervision and to collect finger prick samples to check whether he/she has germs that cause malaria.

Whatever group your child/ward is in, after the last round of SMC we will collect finger prick samples one week later to assess the level of SMC drugs he/she has taken; this will be repeated at either three, four or five weeks after the round then once more at the study conclusion to check for the presence of germs that cause malaria.
In case your child/ward is found after the second SMC round to have parasites that could be transmitted to mosquitoes, we will collect a venous blood sample. The blood sample is used for further examination of malaria parasites and to determine whether the blood can infect mosquitoes. During this infectivity assessment, your child will not have direct contact with the mosquitoes. A small volume of blood is taken from the venous blood sample and offered to the mosquitoes in a glass dispenser.
During the participation of your child/ward to the study, the total volume of blood we will collect will not exceed 16 mL (approximately 3 teaspoons).

Voluntary participation
You are free to decide whether you want to allow your child/ward to be in the study or not. Your decision not to enrol your child/ward in the study will not affect the care you or your child/ward receive at the local health facility any way. Even if you do agree for your child/ward to be in the study, you can withdraw him/her from the study at any time without giving any reason.

What discomforts and risks is my child/ward exposed to?
Your child/ward may feel a little discomfort when the blood is taken and there may be a small bruise or bleeding on the finger or arm where the blood sample is taken. This is considered benign. We will use sterile equipment to take the blood sample and the small wound that may result from the procedure will be treated appropriately. The volume of blood is too small to affect the health of your child/ward and the blood will be quickly replaced by his/her body.

What benefits can I expect from my child/ward participation?
Your child/ward will receive standard antimalarial treatment and medical care for malaria and other non-chronic diseases free of charge during the study period (6 to 7 months).
Participants have free access to the study health facility during the period of their participation, at the clinician's discretion, antimalarials, antibiotics, and treatment for acute illnesses will be provided free of charge. If necessary, hospital care will be provided in the well-equipped wards in Saponé or in a more specialised hospital.

For peer review only - http://bmjopen.bmj.com/site/about/guidelines.xhtml

You will not get paid for your child/ward to be in the study. We will also not pay you when we visit your child/ward at home. However, you will get compensated whenever we ask you to bring your child to the local health facility for us to collect a blood sample for the mosquito feeding assay we have described to you before. You will receive two thousand CFA francs (2,000 FCFA) to compensate the time you lose for working. You will receive the amount at the end of the visit at the health facility.

What about the confidentiality of my child/ward personal data?
The records concerning your child/ward are to be used only for the purpose of this research project. Your child/ward name will not be used on any report resulting from this study. At the beginning of the study, we will give your child/ward a study identification number and this number will be used on the study documents. Any information obtained in connection with this study will be kept strictly confidential and under lock and key. Only senior members of the research team and authorized people such as ethical committee members will have access to your child/ward's records.

What happens to the samples?
The blood samples will be used to test for the germs that cause malaria and the SMC drug levels. Some of the samples will be stored in the freezer in our laboratories before they can be tested. We request your permission to ship some of your child/ward samples to laboratories outside the country for further tests. These countries include the United Kingdom and the Netherlands and possibly other countries. We will store your child/ward samples in the laboratory with a unique number and not with your child/ward name. Therefore, people not involved in the study will not be able to link the sample to your child/ward. We may share your child/ward test results with researchers at other organisations, but we will not give them your child/ward name, address, or any information that could identify your child/ward.

Most of these tests are conducted for research purposes only and cannot be reported back to you or your child/ward's doctor to guide your care.

We will also ask your permission to use the stored samples for future studies that may not be related to this study. These future studies may help find new ways to prevent malaria. If you agree, your child/ward samples may be stored for up to 10 years in Europe (UK and the Netherlands) and in Burkina Faso. After this study has ended, we will remove any means to link the sample to your child/ward's name. If you do not wish to have your child/ward's sample stored for future tests, your child/ward may still participate in our study. You can also change your mind at any time while the study is ongoing.

What about the study results?
This study is being done to help with scientific research and is not for medical diagnosis; therefore we will not provide individual results to you. If, during the course of the study, the study team learns information about your child/ward's health from the study procedures, the study team may discuss this information and your options with you.
A description of this clinical study will be available on https://clinicaltrials.gov/ registry. This web site will not include information that can identify your child/ward.

What if I have questions?
If you have any question concerning this study or concerning your child/ward's rights as a research study participant, do not hesitate to contact the Principal Investigator, Dr

For peer review only - http://bmjopen.bmj.com/site/about/guidelines.xhtml

Alfred B. Tiono [redacted] Results from the study will be communicated to your community when available.
In case you want to contact an independent person, not related to the study, about the research study itself, you can contact the Chairman of Ethics Committee, Prof. Fla KOUETA at [redacted].

For peer review only

For peer review only - http://bmjopen.bmj.com/site/about/guidelines.xhtml

Informed consent agreement

I, ______________________________________ (NAME PARENT/GUARDIAN), having full capacity to consent to the participation of my child/ward ______________________________ (child/ward name) in the study entitled " **INDIE_SMC Understanding and maximizing the community impact of antimalarial treatment** " conducted by principal investigator Dr. Alfred B. Tiono, acknowledge the implications of voluntary participation, the nature, duration and purpose, methods and means by which it is to be conducted. The harms and risks that can reasonably be expected to be encountered have been explained to me by ____________________________________ and are set out in the informed consent form. I have had the opportunity to ask questions about this research and all questions have been answered to my satisfaction. If I have any further questions, I can contact Dr. Alfred B. Tiono (GRAS) at [redacted] I understand that I may at any time during this study revoke my consent and withdraw my child/ward from the study without prejudice; however, my child/ward may be asked to undergo further examinations if, in the opinion of the physician, such examinations are necessary for his/her well-being.

I understand/do not understand the practical implications of this study (circle one)

I agree/disagree that my child/ward should donate blood during the study for the mosquito feeding experiment (circle one)

I agree/disagree that my child/ward's samples be shipped out of the country for analysis related to the study (circle one)

I agree/disagree that my child/ward's samples be stored for future studies (circle one)

I agree/disagree that my child/ward should take part in this study (circle one)

Name of Parent/Guardian : ____

Signature : ___________________

Date : ___________________

Fingerprint if unable to sign

****

Name of the Independent Witness: ____

Signature : _______________________ Date : __________________

****

Name of person obtaining the consent: ____

Signature : __________________________ Date : _________________

For peer review only - http://bmjopen.bmj.com/site/about/guidelines.xhtml

Adult participant to the cross-sectional surveys information and Informed Consent Documents

TITLE OF STUDY: INDIE_SMC Understanding and maximizing the community impact of antimalarial treatment

INSTITUTIONS: Groupe de Recherche Action en Santé (GRAS); London School of Hygiene & Tropical Medicine, London, UK (LSHTM); Radboud university medical center, Nijmegen, the Netherlands (RUMC)

PRINCIPAL INVESTIGATORS: Dr. B. Alfred Tiono (GRAS), Prof. Teun Bousema (LSHTM; RUMC), Prof. Chris Drakeley (LSHTM).

SPONSOR DETAILS: London School of Hygiene and Tropical Medicine, London, UK.

Participant ID: _____________

What is the purpose of this study?

Malaria is caused by parasites that are transmitted by mosquito bites and can be cured by early treatment with malaria drugs. When a human has had an infection for some time, the parasites in their body may be picked up by other mosquitoes when they bite, making that mosquito infectious to other humans. This is known as the cycle of transmission – malaria has to be transmitted from human to mosquito to human and so on, to survive. When a person has been infected with malaria a number of times, they may be protected against some of the symptoms of the disease. When this protection has developed, the infected individual may not show signs of disease, but the parasite will still be present in the body, and may be capable of infecting mosquitoes.

Seasonal Malaria Chemoprevention (SMC) is one of the tools currently used to combat malaria in children. It involves repeated administrations of sulfadoxine-pyrimethamine plus amodiaquine (SPAQ) to children below the age of 5 years during the rainy season which is the peak transmission period in areas of seasonal malaria transmission. Whilst highly impactful in controlled research settings, the impact of SMC in terms of reducing infection prevalence is limited in real-life settings. We therefore want to do this research to understand what are the factors that are hindering the SMC effects in our population. We would also want to know whether extending SMC to all children below 10 years of age will be beneficial to them and moreover to the wider people in the village.

This is why although you are not taking SMC we want to do assessments on you to see if you indirectly benefited from the intervention.

For peer review only - http://bmjopen.bmj.com/site/about/guidelines.xhtml

What are the study procedures?
We aim to include at least 2160 participants (1440 children and 720 adults) in this study.
If you accept to be in the study, we will collect a blood sample by finger prick to assess whether you have the germs that cause malaria in your blood. The total volume will not exceed 4 mL (1 teaspoon).
Your participation will last for one day.

Voluntary participation
You are free to decide whether you want to be in the study or not. Your decision not to participate to the study will not affect the care you receive at the local health facility any way. Even if you do agree to be in the study, you can withdraw from the study at any time without giving any reason.

What discomforts and risks am I exposed to?
You may feel a little discomfort when the blood is taken and there may be a small bruise or bleeding on the finger or arm where the blood sample is taken. This is considered benign. We will use sterile equipment to take the blood sample and the small wound that may result from the procedure will be treated appropriately. The volume of blood is too small to affect your health and the blood will be quickly replaced by your body.

What benefits can I expect from my participation?
You will receive standard antimalarial treatment and medical care for malaria and other non-chronic diseases free of charge during your participation.
Participants have free access to the study health facility during the period of their participation (one day), at the clinician's discretion, antimalarials, antibiotics, and treatment for acute illnesses will be provided free of charge. If necessary, hospital care will be provided in the well-equipped wards in Saponé or in a more specialised hospital.
You will not get paid to be in the study.

What about the confidentiality of my personal data?
The records concerning you are to be used only for the purpose of this research project. Your name will not be used on any report resulting from this study. At enrolment, we will give you a study identification number and this number will be used on the study documents. Any information obtained in connection with this study will be kept strictly confidential and under lock and key. Only senior members of the research team and authorized people such as ethical committee members will have access to your records.

What happens to the samples?
The blood samples will be used to test for the germs that cause malaria and the SMC drug levels. Some of the samples will be stored in the freezer in our laboratories before they can be tested. We request your permission to ship some of your samples to laboratories outside the country for further tests. These countries include the United Kingdom and the Netherlands and possibly other countries. We will store your samples in the laboratory with a unique number and not with your name. Therefore, people not involved in the study will not be able to link the sample to you. We may share your test results with researchers at other organisations, but we will not give them your name, address, or any information that could identify you.

For peer review only - http://bmjopen.bmj.com/site/about/guidelines.xhtml

Most of these tests are conducted for research purposes only and cannot be reported back to you or your doctor to guide your care.

We will also ask your permission to use the stored samples for future studies that may not be related to this study. These future studies may help find new ways to prevent malaria. If you agree, your samples may be stored for up to 10 years in Europe (UK and the Netherlands) and in Burkina Faso. After this study has ended, we will remove any means to link the sample to your name. If you do not wish to have your sample stored for future tests, you may still participate in our study. You can also change your mind at any time while the study is ongoing.

What about the study Results?
This study is being done to help with scientific research and is not for medical diagnosis; therefore we will not provide individual results to you. If, during the course of the study, the study team learns information about your health from the study procedures, the study team may discuss this information and your options with you.
A description of this clinical study will be available on https://clinicaltrials.gov/ registry. This web site will not include information that can identify you.

What if I have questions?
If you have any question concerning this study or concerning your rights as a research study participant, do not hesitate to contact the Principal Investigator, Dr Alfred B. Tiono at [redacted] Results from the study will be communicated to your community when available.
In case you want to contact an independent person, not related to the study, about the research study itself, you can contact the Chairman of Ethics Committee, Prof. Fla KOUETA at [redacted]

For peer review only - http://bmjopen.bmj.com/site/about/guidelines.xhtml

Informed consent agreement

I, ______________________________ (NAME OF THE PARTICIPANT), having full capacity to consent for my participation in the study entitled **" INDIE_SMC Understanding and maximizing the community impact of antimalarial treatment "** conducted by principal investigator Dr. Alfred B. Tiono, acknowledge the implications of voluntary participation, the nature, duration and purpose; methods and means by which it is to be conducted. The harms and risks that can reasonably be expected to be encountered have been explained to me by ______________________________ and are set out in the informed consent form. I have had the opportunity to ask questions about this research and all questions have been answered to my satisfaction. If I have any further questions, I can contact Dr. Alfred B. Tiono (GRAS) at [redacted]. I understand that I may at any time during this study revoke my consent and withdraw from the study without prejudice; however, I may be asked to undergo further examinations if, in the opinion of the physician, such examinations are necessary for my well-being.

I understand/do not understand the practical implications of this study (circle one)

I agree/disagree to donate blood sample during the study (circle one)

I agree/disagree that my samples be shipped out of the country for analysis related to the study (circle one)

I agree/disagree that my samples be stored for future studies (circle one)

I agree/disagree to take part in this study (circle one)

Name of Participant: ______________________________

Signature : ____________________

Date : ____________________

Fingerprint if unable to sign

****

Name of the Independent Witness: ______________________________

Signature : ____________________ Date : ____________________

****

Name of person obtaining the consent: ______________________________

Signature : ____________________ Date : ____________________

For peer review only - http://bmjopen.bmj.com/site/about/guidelines.xhtml

Parent/Guardian of Minor participant to the cross-sectional surveys information and Informed Consent Documents

TITLE OF STUDY: INDIE_SMC Understanding and maximizing the community impact of antimalarial treatment

INSTITUTIONS: Groupe de Recherche Action en Santé (GRAS); London School of Hygiene & Tropical Medicine, London, UK (LSHTM); Radboud university medical center, Nijmegen, the Netherlands (RUMC)

PRINCIPAL INVESTIGATORS: Dr. B. Alfred Tiono (GRAS), Prof. Teun Bousema (LSHTM; RUMC), Prof. Chris Drakeley (LSHTM).

SPONSOR DETAILS: London School of Hygiene and Tropical Medicine, London, UK.

Participant ID: ______________

What is the purpose of this study?

Malaria is caused by parasites that are transmitted by mosquito bites and can be cured by early treatment with malaria drugs. When a human has had an infection for some time, the parasites in their body may be picked up by other mosquitoes when they bite, making that mosquito infectious to other humans. This is known as the cycle of transmission – malaria has to be transmitted from human to mosquito to human and so on, to survive. When a person has been infected with malaria a number of times, they may be protected against some of the symptoms of the disease. When this protection has developed, the infected individual may not show signs of disease, but the parasite will still be present in the body, and may be capable of infecting mosquitoes.

Seasonal Malaria Chemoprevention (SMC) is one of the tools currently used to combat malaria in children. It involves repeated administrations of sulfadoxine-pyrimethamine plus amodiaquine (SPAQ) to children below the age of 5 years during the rainy season which is the peak transmission period in areas of seasonal malaria transmission. Whilst highly impactful in controlled research settings, the impact of SMC in terms of reducing infection prevalence is limited in real-life settings. We therefore want to do this research to understand what are the factors that are hindering the SMC effects in our population. We would also want to know whether extending SMC to all children below 10 years of age will be beneficial to them and moreover to the wider people in the village.

This is why although you are not taking SMC we want to do assessments on you to see if you indirectly benefited from the intervention.

For peer review only - http://bmjopen.bmj.com/site/about/guidelines.xhtml

What are the study procedures?
We aim to include at least 2160 participants (1440 children and 720 adults) in this study.
If you accept your child/ward to be in the study, we will collect a blood sample by finger prick to assess whether your child/ward has the germs that cause malaria in your blood. The total volume will not exceed 4 mL (1 teaspoon).
Your child/ward's participation will last for one day.

Voluntary participation
You are free to decide whether you want your child/ward to be in the study or not. Your decision not to allow your child/ward to participate to the study will not affect the care you/ your child/ward receive at the local health facility any way. Even if you do agree for your child/ward to be in the study, you can withdraw him/her from the study at any time without giving any reason.

What discomforts and risks your child/ward is exposed to?
You child/ward may feel a little discomfort when the blood is taken and there may be a small bruise or bleeding on the finger or arm where the blood sample is taken. This is considered benign. We will use sterile equipment to take the blood sample and the small wound that may result from the procedure will be treated appropriately. The volume of blood is too small to affect your child/ward health and the blood will be quickly replaced by his/her body.

What benefits can I expect from my child/ward participation?
Your child/ward will receive standard antimalarial treatment and medical care for malaria and other non-chronic diseases free of charge during your participation.
Participants have free access to the study health facility during the period of their participation (one day), at the clinician's discretion, antimalarials, antibiotics, and treatment for acute illnesses will be provided free of charge. If necessary, hospital care will be provided in the well-equipped wards in Saponé or in a more specialised hospital. You will not get paid for your child/ward to be in the study.

What about the confidentiality of my child/ward personal data?
The records concerning your child/ward are to be used only for the purpose of this research project. Your child/ward name will not be used on any report resulting from this study. At enrolment, we will give your child/ward a study identification number and this number will be used on the study documents. Any information obtained in connection with this study will be kept strictly confidential and under lock and key. Only senior members of the research team and authorized people such as ethical committee members will have access to your records.

What happens to the samples?
The blood samples will be used to test for the germs that cause malaria and the SMC drug levels. Some of the samples will be stored in the freezer in our laboratories before they can be tested. We request your permission to ship some of your samples to laboratories outside the country for further tests. These countries include the United Kingdom and the Netherlands and possibly other countries. We will store your child/ward samples in the laboratory with a unique number and not with your child/ward name. Therefore, people not involved in the study will not be able to link the sample to your

For peer review only - http://bmjopen.bmj.com/site/about/guidelines.xhtml

child/ward. We may share your child/ward test results with researchers at other organisations, but we will not give them your child/ward name, address, or any information that could identify your child/ward.

Most of these tests are conducted for research purposes only and cannot be reported back to you or your child/ward doctor to guide your care.

We will also ask your permission to use the stored samples for future studies that may not be related to this study. These future studies may help find new ways to prevent malaria. If you agree, your samples may be stored for up to 10 years in Europe (UK and the Netherlands) and in Burkina Faso. After this study has ended, we will remove any means to link the sample to your child/ward name. If you do not wish to have your child/ward sample stored for future tests, you may still allow your child/ward to participate in our study. You can also change your mind at any time while the study is ongoing.

What about the study Results?

This study is being done to help with scientific research and is not for medical diagnosis; therefore we will not provide individual results to you. If, during the course of the study, the study team learns information about your child/ward health from the study procedures, the study team may discuss this information and your options with you.
A description of this clinical study will be available on https://clinicaltrials.gov/ registry. This web site will not include information that can identify your child/ward.

What if I have questions?

If you have any question concerning this study or concerning your child/ward rights as a research study participant, do not hesitate to contact the Principal Investigator, Dr Alfred B. Tiono at [redacted] Results from the study will be communicated to your community when available.
In case you want to contact an independent person, not related to the study, about the research study itself, you can contact the Chairman of Ethics Committee, Prof. Fla KOUETA [redacted]

For peer review only - http://bmjopen.bmj.com/site/about/guidelines.xhtml

Informed consent agreement

I, ____ (NAME OF THE PARENT/GUARDIAN), having full capacity to consent for my child/ward's____ participation in the study entitled " **INDIE_SMC Understanding and maximizing the community impact of antimalarial treatment** " conducted by principal investigator Dr. Alfred B. Tiono, acknowledge the implications of voluntary participation, the nature, duration and purpose; methods and means by which it is to be conducted. The harms and risks that can reasonably be expected to be encountered have been explained to me by ____ and are set out in the informed consent form. I have had the opportunity to ask questions about this research and all questions have been answered to my satisfaction. If I have any further questions, I can contact Dr. Alfred B. Tiono (GRAS) at [redacted] I understand that I may at any time during this study revoke my consent and withdraw my child/ward from the study without prejudice; however, I may be asked that my child/ward undergoes further examinations if, in the opinion of the physician, such examinations are necessary for his/her well-being.

I understand/do not understand the practical implications of this study (circle one)

I agree/disagree for my child/ward to donate blood sample during the study (circle one)

I agree/disagree that my child/ward samples be shipped out of the country for analysis related to the study (circle one)

I agree/disagree that my child/ward samples be stored for future studies (circle one)

I agree/disagree for my child/ward to take part in this study (circle one)

Name of Parent/Guardian: ____

Signature : _______________________

Date : _______________________

Fingerprint if unable to sign

****

Name of the Independent Witness: ____

Signature : ____________________________ Date : ______________________

****

Name of person obtaining the consent: ____

Signature : ________________________________ Date : _____________________

For peer review only - http://bmjopen.bmj.com/site/about/guidelines.xhtml

Supplementary data

Title: Understanding and maximizing the community impact of seasonal malaria chemoprevention in Burkina Faso (INDIE-SMC): study protocol for a cluster randomized evaluation trial

Authors: Moreno M[1*], Barry A[2,3*], Gmeiner M[4*], Yaro JB[2], Sermé SS[2], Byrne I[1], Ramjith J[4], Ouedraogo A[2], Soulama I[2], Grignard L[1], Soremekun S[1], Koele S[4], ter Heine R[4], Ouedraogo AZ[2], Sawadogo J[2], Sanogo E[2], Ouedraogo IN[2], Hien D[2], Sirima SB[2], Bradley J[1], Bousema T[4], Drakeley C[1*], Tiono AB[2*§]

1 Department of Infection Biology, London School of Hygiene and Tropical Medicine, London, UK

2 Groupe de Recherche Action Santé, Ouagadougou, Burkina Faso

3 Secretariat Permanent pour l'élimination du Paludisme, Ministère de la Santé et de l'hygiène publique, Ouagadougou, Burkina Faso

4 Department of Medical Microbiology, Radboud University Medical Centre, Nijmegen, The Netherlands

Table S1. SPAQ dosing according to Burkina Faso MoH. *NA: not applicable.*

Age	SMC Drugs	Day 1	Day 2	Day 3
**3-11 months**	SP : 262.5 mg S, fixed dose fixe with P	1 Tablet	*NA*	*NA*
	AQ : 76.5 mg	1 Tablet	1 Tablet	1 Tablet
**12-59 months**	SP : 525 mg S	1 Tablet	*NA*	*NA*
	AQ : 153 mg	1 Tablet	1 Tablet	1 Tablet
**5-9 years**	SP : 525 mg S	1 Tablet	*NA*	*NA*
	SP : 262.5 mg S	1 Tablet	*NA*	*NA*
	AQ : 153 mg	1 Tablet	1 Tablet	1 Tablet
	AQ : 76.5 mg	1 Tablet	1 Tablet	1 Tablet

For peer review only

Statistical Analysis Plan

Understanding and maximizing the community impact of antimalarial treatment (INDIE-SMC)

Version 1.0

Date: 30 October 2023

For peer review only - http://bmjopen.bmj.com/site/about/guidelines.xhtml

Table of Contents

1 Study details 3
2 Study design 4
2.1 Introduction 4
2.2 Study rationale 4
2.3 Design & intervention 5
3 Randomization 6
4 Study objectives 6
5 Study endpoints 7
5.1 Primary endpoint 7
5.2 Secondary endpoint 7
5.3 Exploratory endpoints 8
6 Study population 8
7 Sample size considerations 8
8 Data entry 10
9. Coding 10
10 Statistical methods 11
10.1 Demographics 11
10.2 Study flow 11
10.3 Effectiveness of SMC and SMC efficacy decay 11
10.4 Human infectious reservoir 12
10.5 Drug resistance 12
10.6 Social barriers and enablers 12
10.7 Analysing data and reporting results 13
11 Conduct of analyses 16
11.1 Programming 16
12 Reporting 16
13 Document history 16
14 Signature page 17
15 References 18

For peer review only - http://bmjopen.bmj.com/site/about/guidelines.xhtml

1 Study details

Protocol Title
Understanding and maximizing the community impact of antimalarial treatment (INDIE-SMC)

Study code
INDIE-SMC

Trial Register Identifier
NCT05878366

SAP Authors
Markus Gmeiner, Marta Moreno, Teun Bousema, John Bradley, Chris Drakeley

Investigator
Alfred B. Tiono

Groupe de Recherche Action Santé (GRAS), Burkina Faso

Sponsor (representative)
Chris Drakeley

London School of Hygiene and Tropical Medicine (LSHTM), United Kingdom

For peer review only

For peer review only - http://bmjopen.bmj.com/site/about/guidelines.xhtml

Table 1: Abbreviations

Abbreviation	Full form
AQ	Amodiaquine
CRF	Case Report Form
DEAQ	Desethylamodiaquine
DOT	Directly Observed Treatment
FGD	Focus Group Discussion
GRAS	Groupe de Recherche Action Santé
IDI	In-Depth Interview
IQR	Interquartile Range
KII	Key Informant Interview
LLIN	Long Lasting Insecticide Net
LSHTM	London School of Hygiene and Tropical Medicine
MoH	Ministry of Health
ODK	Open Data Kit
qRT PCR	Quantitative Real-Time Polymerase Chain Reaction
SAP	Statistical Analysis Plan
SMC	Seasonal Malaria Chemoprevention
SPAQ	Sulfadoxine-Pyrimethamine plus Amodiaquine

2 Study design

2.1 Introduction

The Statistical Analysis Plan (SAP) describes the statistical analyses of data obtained in the INDIE-SMC study. It is a controlled document and may be updated until the final analysis is performed.

2.2 Study rationale

Seasonal Malaria Chemoprevention (SMC) currently involves repeated administrations of sulfadoxine-pyrimethamine plus amodiaquine (SPAQ) to children below the age of 5 years during the peak transmission season in areas of seasonal malaria transmission. Whilst highly impactful in reducing the burden of *P. falciparum* malaria in controlled research

For peer review only - http://bmjopen.bmj.com/site/about/guidelines.xhtml

settings, the impact of SMC in terms of reducing infection prevalence is limited in real-life settings.

It is currently unclear whether failure of SMC with SPAQ to prevent malaria parasitaemia is associated with

i) imperfect adherence;

ii) parasite resistance to the drug components;

iii) active drug concentrations that are achieved in SMC participants;

iv) consistent exposure to reinfection.

It is also unclear whether extending the age-range targeted by SMC will reduce the infectious reservoir for transmission to a level where community-benefits may be expected. It is further currently unclear what factors determine (cascades in) efficacy decay and whether SMC coverage and uptake is equitable or affected by, for example, gender of children in the target age group. Quantifying each of these elements should contribute to understanding the effectiveness of SMC in programmatic settings.

2.3 Design & intervention

The study is designed to evaluate SMC effectiveness and consists of three interventional study arms, with the first arm receiving an intervention that is implemented as part of routine control.

Study arm 1:	SMC in children under the age of 5 years, implemented by the Ministry of Health (MoH) without directly observed treatment for the full course of SMC
Study arm 2:	SMC in children under the age of 5 years, with directly observed treatment for the full course of SMC
Study arm 3:	SMC in children under the age of 10 years, with directly observed treatment for the full course of SMC

SMC consists of SP and AQ administered daily for three days. SP is given on the first SMC day only while AQ is given daily over three days. In total 4 rounds of SMC will be conducted during the peak malaria transmission season from July to October, separated by approx. 30 days.

Each study arm contains 62 clusters; each cluster consists of 3 compounds where each compound has at least one child aged 3-59 months and one child aged 5-9 years. (186 compounds per arm; 558 compounds in total). Differences in SMC coverage and efficacy will be determined in the SMC-targeted age groups. In contrast, all age groups will take part in the evaluation of the study to assess the impact of the intervention on the infectious reservoir in the community and compare parasite carriage in targeted and untargeted populations. To detect possible behavioural contamination and differences in SMC uptake or adherence in study arm 1 compared to what would be observed if there would be no

For peer review only - http://bmjopen.bmj.com/site/about/guidelines.xhtml

study team present, a single cross-sectional survey 7 days after the last (4th) round of SMC coverage is assessed outside the study area (2-5 kilometres from the nearest enrolled study compound) by questionnaire and samples to determine plasma levels of SMC drugs and parasite carriage by qPCR. Plasma levels of SMCR drugs are used as the most definitive evidence of differences in SMC uptake/adherence.

Scheduled surveys and scheduled or unscheduled sampling timepoints will take place in each arm; these surveys are designed and timed for several outcome measures and include:

i) a survey prior to first SMC roll-out (performed in all compounds).

ii) mid-study surveys 3, 4 and 5 weeks after the last SMC roll out (each compound will participate in one of these surveys).

iii) end-of-study survey up to 10 weeks after last SMC roll out (performed in all compounds).

iv) sampling for drug metabolite assessment shortly after the last SMC round (sample collection in all compounds in the study arms and outside-study control area).

v) sampling for transmission assay on first day of SMC round 2 to 4 (sampling in all compounds; selection from individuals aged 5-9 years).

vi) passive case detection in all four health facilities that are accessed by the study population throughout the study period (from enrolment until the end of follow-up).

3 Randomization

The unit of randomization will be the cluster; each cluster comprises of 3 compounds. Clusters are randomly allocated to one of the three study arms by a computer generated algorithm and stratified by village. Stratification by village was to account for spatial differences in malaria exposure, distance from health facilities, and treatment seeking behavior. In total, 185 clusters were randomized.

4 Study objectives

The specific study objectives are to:

i) compare SMC effectiveness in children aged 3-59 months as implemented by the national malaria control program and SMC implemented in a research context where all doses are directly observed.

ii) Evaluate the effectiveness of SMC to reduce malaria infection prevalence in 5-9 year olds

For peer review only - http://bmjopen.bmj.com/site/about/guidelines.xhtml

iii) quantify the infectious reservoir and the contribution of different age groups to transmission with conventional SMC (<5 years) and extended SMC (<10 years)

iv) determine the impact of drug resistance (by molecular typing of *dhps*, *dhfr, pfcrt* and *pfmdr1*) and drug absorption on SMC efficacy

v) understand social barriers and enablers interfering with SMC efficacy and how SMC uptake is related to health equity with special attention to gender inequalities.

vi) quantify SMC efficacy decay under programmatic conditions and key drivers of this decay.

5 Study endpoints

5.1 Primary endpoint

- Parasite prevalence by quantitative PCR (qPCR) at the end of the transmission season in age groups targeted by seasonal malaria chemoprevention. [end-of-study survey].

This endpoint will be compared between arms 1 and 2 (in children aged 3-59 months) and arms 2 and arm 3 (in children aged 5 years-9 years). [end-of-study survey].

5.2 Secondary endpoint

- Parasite prevalence by qPCR at the end of the transmission season in all age groups (between arm comparison). [end-of-study survey].
- Parasite prevalence by qPCR at the end of the transmission season compared between arms 1 and combined arms 2 + 3. [end-of-study survey].
- Parasite prevalence by microscopy prior to SMC rounds 2, 3 and 4 in SMC-targeted age groups (between arm comparison). [at SMC-delivery].
- Rate of re-infection with *P. falciparum* at weeks 3, 4 and 5 after the last round of SMC, assessed in SMC-targeted age groups (between arm comparison). [mid-study surveys].
- Gametocyte prevalence by qRT-PCR at weeks 3, 4 and 5 after the last round of SMC, assessed in SMC-targeted age groups (between arm comparison). [mid-study surveys].
- Gametocyte prevalence by qRT-PCR at the end of the transmission season in all age groups (between arm comparison). [end-of-study survey].
- Gametocyte prevalence by qRT-PCR at the end of the transmission season in age groups targeted by SMC (comparison between arm1 and 2 (in children aged 3-59 months) and arms 2 and arm 3 (in children aged 5 years-9 years). [end-of-study survey].
- Plasma levels of AQ and desethylamodiaquine (DEAQ) after the 4th round of SMC in children aged 3 months-9 years (between arm comparison) [post-SMC round 4].
- Clinical malaria incidence captured during passive-case detection (between arm comparison) [[post-SMC round 4]

For peer review only - http://bmjopen.bmj.com/site/about/guidelines.xhtml

5.3 Exploratory endpoints

- Infectivity to mosquitoes, defined as the percentage of infected mosquitoes, in relation to gametocyte density and plasma drug levels of AQ and DEAQ (across arms assessment) [during and shortly after SMC rounds 2-4].
- Size and age-distribution of the infectious reservoir for malaria, defined as the likelihood that a mosquito becomes infected with malaria parasites after feeding on a population member (between arm comparison) [during the study].
- Prevalence of drug resistance markers in infected children aged 3 months-9 years assessed post each round of SMC (between arm comparison) [during the entire study].
- Description of perceived social barriers to SMC uptake [during the entire study].
- Quantification of SMC efficacy decay under programmatic conditions [during the entire study].

6 Study population

The study population will be all members of 555 compounds (185 clusters of 3 compounds) in Saponé Health District (Burkina Faso). Clusters are randomized over 3 study arms. Compound members of all ages will be included in study procedures (e.g. evaluation of the intervention) but only children will be targeted with the intervention. Based on census data, and recent studies in the same study area, an average of 1.5 children aged <5 years and 1.5 child 5-9 years per compound is expected (4.5 and 4.5 per cluster, respectively).

In addition, 120 randomly selected compounds outside of the study area are included for a single survey after the last round of SMC.

7 Sample size considerations

Evaluable study clusters will comprise 3 compounds and have between 4-5 children below 5 years of age (eligible for standard SMC) and similar numbers of children aged 5-10 years (eligible for SMC in study arm 3). We assume ~40% parasite prevalence detected by PCR at the end of the transmission season in children aged 5-10 without SMC and 16% parasite prevalence in children aged <5 years under programmatic SMC (based on recently completed work in the same area Collins, Ouedraogo et al. in prep). When assuming a reduction to 25% parasite prevalence in children aged 5-10 years when included in SMC, a sample size of 55 clusters per arm (x3 compounds per cluster and an estimated total 6 children in this age group per cluster) will achieve 87% power to detect this difference at an alpha of 0.05 and a coefficient of variation of 0.3 (data from INDIE 1a; LSHTM EC #14724; Centre National de Recherche et de Formation sur le Paludisme institutional review board (Deliberation N° 2018/000002/MS/SG/CNRFP/CIB; Burkina Faso national medical ethics committee (Deliberation N° 2018-01-010)). This sample size will also allow the detection of a reduction of parasite prevalence in directly observed SMC to 7% as

For peer review only - http://bmjopen.bmj.com/site/about/guidelines.xhtml

compared to 16% under programmatic SMC, in children under 5 years of age. The total number of compounds per arm has been increased to 62 to mitigate the effects of non-compliance and drop-outs and also for logistical reasons, such whole villages are included. We will thus enrol a total of 186 clusters over 3 arms in a ratio of 1:1:1 with 558 compounds in total. We anticipate that this will include a minimum of 720 children below 5 years of age (2 per compound, based on recent census data) and 720 children aged 5-9 years (1 per compound as per inclusion criterion).

For assessments of the human infectious reservoir, the intervention study is too large in scope (i.e. 558 compounds, compared to 180 in the previous INDIE project) and the time-window for transmission assays is too narrow (i.e. transmission assays are ideally done within weeks after SCM administration) to assess a direct impact of SMC on gametocyte infectivity with a conventionally powered comparison between arms. Thus a combination of methodologies (quantification of the association between gametocyte density and infectivity in the absence of treatment; imputed mosquito transmission from gametocyte density) are used (i, ii) It was further shown that direct comparisons of the association between gametocyte density and mosquito infection rates in mosquito feeding assays can demonstrate whether imputation is indeed acceptable across populations (i*)* or whether there are subpopulations with a different association (for example due to gametocyte-sterilizing effects of antimalarials (iii)). These insights will be used to perform mosquito feeding assays on gametocyte positive individuals with and without prior SMC and compare mosquito infection rates for a given gametocyte density (iii). Since the hypothesis is that SPAQ will permanently sterilize gametocytes, any reduced infectivity of gametocytes will remain apparent even after drug levels have waned (i.e. the gametocytes are permanently damaged by drug exposure) (iv). A possible sterilizing effect of SPAQ can be assessed by preferentially recruiting children who are gametocyte positive at the start of SMC and invite them for mosquito feeding post SMC. This will be done in children aged 5-9 years only who carry the highest parasite densities and highest gametocyte prevalence/density (i); this also avoids phlebotomy from the youngest children. A median gametocyte density of ~14 gametocytes/µL (IQR 1.8-44.2) as previously observed post-SMC (v, vi) is anticipated. Whilst the exact number of experiments that can be conducted will be dictated by mosquito husbandry, simulations indicate that when recruiting 100 children who received SPAQ (arm 3) and 100 children who did not (arms 1 & 2) and dissecting 40 mosquitoes per experiment, we would have >90% power to detect a 70% reduction in infectivity (vii).

An exact sample size justification for the single cross-sectional survey in the area outside the study-targeted population is complicated; this survey is performed to give a broad indication of the potential influence the presence of our study team may have on the operational delivery of SMC in arm 1. It is not part of any main comparisons. Estimates of adherence are based on questionnaire data, supported by measurements of DEAQ drug levels in plasma. In arm 2 and 3 (Directly Observed Treatment (DOT); by design full adherence), the median level of DEAQ at day 4 following the last dose of AQ is expected to be 550 ng/mL (IQR 350-850)[14]. Based on a large assessment of reported adherence and measured AQ/DEAQ plasma levels following SMC in Niger (viii), we anticipate that <50% of children show complete adherence in arms 1 and the control village and >20% of the children in these populations may fail to take any dose. If we conservatively assume that

For peer review only - http://bmjopen.bmj.com/site/about/guidelines.xhtml

this results in a reduction of median DEAQ levels to <400ng/mL (IQR 200-700), we can estimate the power to detect this difference when enrolling 120 children in the SMC-targeted age range in the control area. For this, we assume that these DEAQ levels stem from right-skewed (log-normal) distributions; giving median log DEAQ concentrations of 6.31 (IQR 5.86-6.75) for DOT and 5.99 (IQR 5.30-6.55) for non-DOT. If it is further assumed that the distribution of the logged values is normally distributed, we can estimate the standard deviation through the IQR as IQR/1.349, giving standard deviations of 0.93 and 0.66, respectively. Using a sample size of 120 per group, and a Welch's two sample t-test for testing the differences between the log DEAQ concentrations, we estimate a power of 86.3% to detect these differences. Based on these assumptions, we are confident that sampling 120 children <5 years of age will allow meaningful assessments. As indicated above, this sample size estimation is not relevant for any of the main comparisons in the study but merely used to ensure informative sampling and avoid exposing an unnecessarily large population to the small discomfort of a single finger prick sample. We will thus aim to determine plasma levels of at least 120 children <5 years in arm 1;120 children <5 years from arms 2 and 3 combined and 120 children from the control area.

Drug plasma levels are also relevant for other comparisons (e.g. the impact on transmission efficiency in mosquito feeding assays, the role in explaining clinical malaria episodes and asymptomatic parasite carriage after SMC. For these comparisons, samples may be selected in a case-control manner.

8 Data entry

All study data from the baseline survey up until the end-of-study survey are entered into an electronic data capture system (ODK) by the investigators and delegated personnel, either directly (in which case these are themselves considered Source data) and/or from a designated primary Source, e.g. paper Case Report Forms (CRF), Blood Smear read out results.

9. Coding

Data coding for not standardized data is not applied.

All data in the database is pseudonymized. A study participant identification link log is available at the investigators' site.

For peer review only - http://bmjopen.bmj.com/site/about/guidelines.xhtml

10 Statistical methods

10.1 Demographics

Baseline characteristics (age, gender) will be tabulated. Categorical variables will be presented as count and percentage. Numerical variables will be summarized as median, minimum and maximum. GPS data and maps will be used to present location of compounds and clusters.

10.2 Study flow

A study flow chart (e.g. CONSORT flow chart) will be used to present number of volunteers approached for enrolment, enrolled, withdrawn and followed until completion of the study.

10.3 Effectiveness of SMC and SMC efficacy decay

Assessing effectiveness of SMC requires collection of data on:

- *Coverage (proportion of children reached by the four SMC rounds)*
- *Health Care worker's compliance (proportion of children receiving first dose of SMC under DOT)*
- *Caregiver's compliance (proportion of children receiving correct SMC regimen)*
- *Drug levels (proportion of children with adequate drug level)*

<u>SMC treatment coverage</u>

SMC coverage will be assessed through a combination of direct observation of treatment and pharmacological assessments. In arm 1 Directly Observed Treatment (DOT) is applied only on the first day of each SMC round. In arm 2 and 3 DOT is applied for the full three-day course of SMC. DOT includes observation of drug administration and a 15 minutes post-administration observation period. In arms 2 and 3, coverage will be expressed as percentage of the target population receiving at least one dose of SMC, at least two doses of SMC and the full 3-day course of SMC.

The adherence for all study participants will be evaluated based on observed amodiaquine (AQ) and desethylamodiayiune (DEAQ) plasma concentrations 4 days after the last dose of SMC should have been taken (day 7 since the start of the SMC round), as previously described by Ding J. et al. (2020 (viii)).

For peer review only - http://bmjopen.bmj.com/site/about/guidelines.xhtml

Qualitative data on compliance

See section 10.6

Biomarkers:

Serological analysis of blood samples will be conducted using bead based assays to assess presence of biomarkers for correlates of infection (i.e. proteins associated with inflammation or parasite density (HRP2)). These biomarkers, assessed at individual level will be related to parasite positivity, transmission efficiency etc. as part of exploratory analyses.

10.4 Human infectious reservoir

The human infectious reservoir will be assessed by a combination of direct measurements of transmissibility (assessed in a selection of parasite-positive children aged 5-9 years) and modeling/transmission imputation as described in detail in section 10.7 endpoint 12.

10.5 Drug resistance

Nucleic acids will be extracted with commercial kits [Qiagen] from blood samples collected on filter paper. Three dual-labelled probes were designed to detect three crt genotypes at codons 72–76 (encoding Cys-Val-Met-Asn-Lys [CVMNK], CVIET, and SVMNT).

10.6 Social barriers and enablers

The qualitative component of the study is designed to understand potential factors that influence SMC uptake and effectiveness.
Throughout the study, data on compliance to SMC treatment guidelines from caregiver and healthcare worker perspectives are collected. Whether first dose was directly observed and whether the full treatment course was administered to the child after each round on SMC is documented. After SMC round 4 a cross-sectional survey will be carried out. For this, individual interviews of caregivers of children targeted or not by the current SMC strategy will be conducted.

Further, within each of the study arms, between 6-8 individuals will be recruited for focus group discussions (FGDs). Community-based field workers will be asked to purposively select individuals for the FGD. Preference will be given for eligible participants that are active in community life and events, and therefore would have had greater exposure to views and experiences of other community members. The selection will pay much attention to gender balance. The FGD will be closed to outside observers, limited only to consenting participants and the fieldworkers conducting the session. Two field workers will conduct the FGD. One field worker will lead the discussion and the other will take notes. The discussion will be recorded for transcription. A semi-structured discussion guide will be used to guide the

For peer review only - http://bmjopen.bmj.com/site/about/guidelines.xhtml

discussion. The discussion will last approximately 60 minutes. The recording of the discussion will be used to create a discussion transcript for data analysis.

Key informant interviews (KIIs) and in-depth interviews (IDIs) will be carried out. For the KIIs, participants will be recruited through purposive sampling to include stakeholders within the health system involved in SMC implementation.
The IDIs will be conducted among 5-10 key informants per each of the study arms. These individuals will also be purposively selected using community-based field workers. The aim is to include community leaders, professionals, or residents who have first-hand knowledge about the community. The interviewer will use a semi-structured interview guide to guide the discussion that will last approximately 60 minutes. The interview will be recorded for transcription. The recording of the interview will be used to create a discussion transcript for data analysis.

10.7 Analysing data and reporting results

When analysing data based on age, participants' age at time of the first round of SMC is used. If a child received SMC because he/she was <5 years at the time of SMC but turns 5 prior to the last survey, the age at the moment of SMC round 1 is used since this dictated the intervention.

Primary endpoint

1. *Parasite prevalence by quantitative PCR (qPCR) at the end of the transmission season in age groups targeted by seasonal malaria chemoprevention. [end-of-study survey].*

This endpoint will be compared between arms 1 and 2 (in children aged 3-59 months) and arms 2 and arm 3 (in children aged 5 years--9 years). Parasite prevalence by qPCR will be used as binary variable with all parasite densities above 100 parasites/mL (0.1 parasite/μL) being classified as parasite positive. Parasite prevalence is compared between arms by logistic regression; models will account for clustering by including cluster as a random intercept and village as a fixed effect.

Secondary endpoints

2. *Parasite prevalence by qPCR at the end of the transmission season in all age groups (between arm comparison). [end-of-study survey].*

This outcome will be compared between arms as described above for outcome 1.

3. *Parasite prevalence by qPCR at the end of the transmission season compared between arms 1 and combined arms 2 + 3. [end-of-study survey].*

This outcome will be compared between arms as described above for outcome 1.

For peer review only - http://bmjopen.bmj.com/site/about/guidelines.xhtml

4. *Parasite prevalence by microscopy prior to SMC rounds 2, 3 and 4 in SMC-targeted age groups (between arm comparison). [at SMC-delivery].*

This outcome will be compared between arms as described above for outcome 1.

5. *Rate of re-infection with P. falciparum at weeks 3, 4 and 5 after the last round of SMC, assessed in SMC-targeted age groups (between arm comparison). [mid-study surveys].*

This outcome will be presented as prevalence estimates with 95% confidence intervals for each time-point. It will be compared between arms as described above for outcome 1.

6. *Gametocyte prevalence by qRT-PCR at weeks 3, 4 and 5 after the last round of SMC, assessed in SMC-targeted age groups (between arm comparison). [mid-study surveys].*

This outcome will be presented as prevalence estimates with 95% confidence intervals for each time-point and study arm separately. It will be compared between arms as described above for outcome 1.

7. *Gametocyte prevalence by qRT-PCR at the end of the transmission season in all age groups (between arm comparison). [end-of-study survey].*

This outcome will be presented as prevalence estimate with 95% confidence interval for each study arm separately. It will be compared between arms as described above for outcome 1.

8. *Gametocyte prevalence by qRT-PCR at the end of the transmission season in age groups targeted by SMC (comparison between arm1 and 2 (in children aged 3-59 months) and arms 2 and arm 3 (in children aged 5 years-9 years). [end-of-study survey].*

This outcome will be presented as prevalence estimates with 95% confidence intervals for each time-point and study arm separately. It will be compared between arms as described above for outcome 1.

9. *Plasma levels of AQ and desethylamodiaquine (DEAQ) after the 4th round of SMC in children aged 3 months-9 years (between arm comparison) [post-SMC round 4].*

A published non-linear mixed-effects pharmacokinetic model will be evaluated for the ability to describe AQ and DEAQ pharmacokinetics in the study population (viii). All quantifiable plasma concentrations available will be used for model evaluation using gold-standard methods. Visual predictive checks and goodness-of-fit plots will be made to assess model performance for participants in which dosing history is observed through DOT. For each study participant 2000 full concentration-time profiles will be simulated, considering participant age and weight, assuming full treatment adherence. Model-predicted concentrations at the PK sampling time will be determined for each simulation. The optimal cut-off percentile discriminating between adherence and non-adherence was previously determined to be the 20th percentile of the individual simulated concentration-time profiles (71% sensitivity, 80% sensitivity) (viii).

For peer review only - http://bmjopen.bmj.com/site/about/guidelines.xhtml

Participants with observed plasma concentrations below the individual cut-off percentile will be scored as non-adherent. Coverage will be expressed as percentage of participants scored as non-adherent per treatment arm. Additional analyses will include the percentage of participants with/without any measurable AQ or DEAQ plasma concentrations.

10. *Incidence of clinical malaria captured during passive-case detection (between arm comparison) [[post-SMC round 4]*

Passive case detection data will be analysed by Poisson regression. The outcome will be the number of cases in each compound. Comparisons will be made between arms 1 and 2 in children aged 3-59 months and between arms 2 and 3 in children aged 5-9 years. There will be a random effect for compound and an offset of the number of children.

Exploratory endpoints

11. *Infectivity to mosquitoes, defined as the percentage of infected mosquitoes, in relation to gametocyte density and plasma drug levels of AQ and DEAQ (across arms assessment) [during and shortly after SMC rounds 2-4].*

The association between proportion of infected mosquitoes and gametocyte density will determined using generalized linear models assuming a binomial distribution with a log-link for participants with and without detectable AQ/DEAQ plasma concentrations. In addition, infectivity will be compared between individuals with/without AQ/DEAQ plasma levels using a previously published statistical model (ix). In case of low infectivity, the nonparametric van Elteren's test, an extension of the Wilcoxon rank sum test that compares groups in a stratified manner will be used (iii)where we compare infectivity between groups after stratifying in gametocyte density categories. Infectivity is then compared within different bins to test for differences between arms, accounting for gametocyte density. Additional analyses will take into consideration AQ/DEAQ concentrations at continuous scale (rather than simple detectability).

12. *Size and age-distribution of the infectious reservoir for malaria, defined as the likelihood that a mosquito becomes infected with malaria parasites after feeding on a population member (between arm comparison) [during the study].*

The size and shape of the infectious reservoir will be estimated using a combination of directly observed mosquito infection events and imputed transmissibility (i, ii). This imputed transmissibility will use gametocyte density (measured for all relevant timepoints) as starting point. The association between proportion of infected mosquitoes and gametocyte density will determined using generalized linear models assuming a binomial distribution with a log-link. If drug levels do not influence infectivity (see 8), a single modelled association will be used. If drug levels do have a statistically significant impact on transmission potential, all estimates of the infectious reservoir within one month of SMC will take study arm into account. For the infectious reservoir at the final end of study survey, no impact of SMC drugs on transmission efficiency is assumed. The

For peer review only - http://bmjopen.bmj.com/site/about/guidelines.xhtml

contribution of different age groups (younger than 5 years, 5–15 years, 16 years and older) to the infectious reservoir will be estimated using all data (including parasite-negative observations where zero transmission is assumed)

13. *Prevalence of drug resistance markers in infected children aged 3 months-9 years assessed post each round of SMC (between arm comparison) [during the entire study].*
14. *Description of perceived social barriers to SMC uptake [during the entire study].*
15. *Quantification of SMC efficacy decay under programmatic conditions [during the entire study].*

For peer review only

11 Conduct of analyses

11.1 Programming

Analysis will be performed using STATA software and R. Pharmacokinetic model evaluation and adherence simulations will be performed using NONMEM 7.5 with Pirana 2.9.9 as interface.

12 Reporting

Ethics committees at LSHTM and in Burkina Faso will receive annual reports on study progress. Final results will be uploaded to clinicaltrials.gov upon publishing of these results in a peer-reviewed journal.

13 Document history

Version	Author	Description of change	Date
1.0	Markus Gmeiner	New document	30 OCT 2023

For peer review only - http://bmjopen.bmj.com/site/about/guidelines.xhtml

14 Signature page

Date:	
Name (*Principal Investigator*):	*Alfred B. Tiono*
Signature:	

Date:	
Name (*Sponsor Representative*):	*Chris Drakeley*
Signature:	

For peer review only

For peer review only - http://bmjopen.bmj.com/site/about/guidelines.xhtml

15 References

[i] Andolina, C., Rek, J. C., Briggs, J., Okoth, J., Musiime, A., Ramjith, J., Teyssier, N., Conrad, M., Nankabirwa, J. I., Lanke, K., Rodriguez-Barraquer, I., Meerstein-Kessel, L., Arinaitwe, E., Olwoch, P., Rosenthal, P. J., Kamya, M. R., Dorsey, G., Greenhouse, B., Drakeley, C., Staedke, S. G., … Bousema, T. (2021). Sources of persistent malaria transmission in a setting with effective malaria control in eastern Uganda: a longitudinal, observational cohort study. *The Lancet. Infectious diseases*, *21*(11), 1568–1578. https://doi.org/10.1016/S1473-3099(21)00072-4

[ii] Rek, J., Blanken, S. L., Okoth, J., Ayo, D., Onyige, I., Musasizi, E., Ramjith, J., Andolina, C., Lanke, K., Arinaitwe, E., Olwoch, P., Collins, K. A., Kamya, M. R., Dorsey, G., Drakeley, C., Staedke, S. G., Bousema, T., & Conrad, M. D. (2022). Asymptomatic School-Aged Children Are Important Drivers of Malaria Transmission in a High Endemicity Setting in Uganda. *The Journal of infectious diseases*, *226*(4), 708–713. https://doi.org/10.1093/infdis/jiac169

[iii] Bradley, J., Soumaré, H. M., Mahamar, A., Diawara, H., Roh, M., Delves, M., Drakeley, C., Churcher, T. S., Dicko, A., Gosling, R., & Bousema, T. (2019). Transmission-blocking Effects of Primaquine and Methylene Blue Suggest Plasmodium falciparum Gametocyte Sterilization Rather Than Effects on Sex Ratio. *Clinical infectious diseases : an official publication of the Infectious Diseases Society of America*, *69*(8), 1436–1439. https://doi.org/10.1093/cid/ciz134

[iv] Alkema, M., Reuling, I. J., de Jong, G. M., Lanke, K., Coffeng, L. E., van Gemert, G. J., van de Vegte-Bolmer, M., de Mast, Q., van Crevel, R., Ivinson, K., Ockenhouse, C. F., McCarthy, J. S., Sauerwein, R., Collins, K. A., & Bousema, T. (2021). A Randomized Clinical Trial to Compare Plasmodium falciparum Gametocytemia and Infectivity After Blood-Stage or Mosquito Bite-Induced Controlled Malaria Infection. *The Journal of infectious diseases*, *224*(7), 1257–1265. https://doi.org/10.1093/infdis/jiaa157

[v] Dicko, A., Roh, M. E., Diawara, H., Mahamar, A., Soumare, H. M., Lanke, K., Bradley, J., Sanogo, K., Kone, D. T., Diarra, K., Keita, S., Issiaka, D., Traore, S. F., McCulloch, C., Stone, W. J. R., Hwang, J., Müller, O., Brown, J. M., Srinivasan, V., Drakeley, C., … Bousema, T. (2018). Efficacy and safety of primaquine and methylene blue for prevention of Plasmodium falciparum transmission in Mali: a phase 2, single-blind, randomised controlled trial. *The Lancet. Infectious diseases*, *18*(6), 627–639. https://doi.org/10.1016/S1473-3099(18)30044-6

[vi] Stone, W., Mahamar, A., Smit, M. J., Sanogo, K., Sinaba, Y., Niambele, S. M., Sacko, A., Keita, S., Dicko, O. M., Diallo, M., Maguiraga, S. O., Samake, S., Attaher, O., Lanke, K., Ter Heine, R., Bradley, J., McCall, M. B. B., Issiaka, D., Traore, S. F., Bousema, T., … Dicko, A. (2022). Single low-dose tafenoquine combined with dihydroartemisinin-piperaquine to reduce Plasmodium falciparum transmission in Ouelessebougou, Mali: a phase 2, single-blind, randomised clinical trial. *The Lancet. Microbe*, *3*(5), e336–e347. https://doi.org/10.1016/S2666-5247(21)00356-6

[vii] Ramjith, J., Alkema, M., Bradley, J., Dicko, A., Drakeley, C., Stone, W., & Bousema, T. (2022). Quantifying Reductions in *Plasmodium falciparum* Infectivity to Mosquitos: A Sample Size Calculator to Inform Clinical Trials on Transmission-Reducing Interventions. *Frontiers in immunology*, *13*, 899615. https://doi.org/10.3389/fimmu.2022.899615

[viii] Ding, J., Coldiron, M. E., Assao, B., Guindo, O., Blessborn, D., Winterberg, M., Grais, R. F., Koscalova, A., Langendorf, C., & Tarning, J. (2020). Adherence and Population Pharmacokinetic Properties of

For peer review only - http://bmjopen.bmj.com/site/about/guidelines.xhtml

Amodiaquine When Used for Seasonal Malaria Chemoprevention in African Children. *Clinical pharmacology and therapeutics*, *107*(5), 1179–1188. https://doi.org/10.1002/cpt.1707

[ix] Bradley, J., Stone, W., Da, D. F., Morlais, I., Dicko, A., Cohuet, A., Guelbeogo, W. M., Mahamar, A., Nsango, S., Soumaré, H. M., Diawara, H., Lanke, K., Graumans, W., Siebelink-Stoter, R., van de Vegte-Bolmer, M., Chen, I., Tiono, A., Gonçalves, B. P., Gosling, R., Sauerwein, R. W., … Bousema, T. (2018). Predicting the likelihood and intensity of mosquito infection from sex specific *Plasmodium falciparum* gametocyte density. *eLife*, *7*, e34463. https://doi.org/10.7554/eLife.34463

For peer review only

For peer review only - http://bmjopen.bmj.com/site/about/guidelines.xhtml
